# Intramolecular Al/P Frustrated Lewis Pairs Based on an Indoline Backbone—Two Reaction Sites for Small Molecule Activation and Hydrodefluorination

**DOI:** 10.1021/acs.inorgchem.6c01812

**Published:** 2026-06-17

**Authors:** Sanjay Biswas, Siad Wolff, Beatrice Cula, Christian Limberg

**Affiliations:** Institut für Chemie, 9373 Humboldt-Universität zu Berlin, Brook-Taylor-Straße 2, 12489 Berlin, Germany

## Abstract

The synthesis of compounds of the type Ph_2_P­(Ind)­AlX_2_ (with Ind = indoline and X = Cl, I, Me, and C_6_F_5_) is reported. They can be accessed starting from the 7-diphenylphosphino indoline via deprotonation and subsequent salt metathesis or via in situ deprotonation using precursors with basic ligands. While in the case of X = Me, X-ray crystallographic and solution studies confirmed that the connectivity and configuration are as envisaged, the latter was only true in the case of X = C_6_F_5_ for the solid state. In solution, ligand scrambling was observed, as was for the representatives with X = I and Cl, which unlike the organometallic derivatives were found to have a dimeric structure. The dimers in solution are partly forming the scrambled compounds [Ph_2_P­(Ind)]_2_AlX. However, upon contact with donors (D), all compounds were found to react as Ph_2_P­(Ind)­AlX_2_, forming Ph_2_P­(Ind)­AlX_2_(D). The reactivities of the representatives with X = Me and C_6_F_5_ were investigated, showing that diazo compound Ph_2_CN_2_ can be activated in the Al/P reaction space, while CO_2_ reacts with the aluminum-amide unit to give carbamates. The Al–N units can also be used to perform the dehydrofluorination of fluorocyclohexane. Subsequent investigations showed that achieving this reaction with alanes requires a fine balance and cooperation between a sufficiently Lewis-acidic aluminum center and a basic ligand.

## Graphical abstract

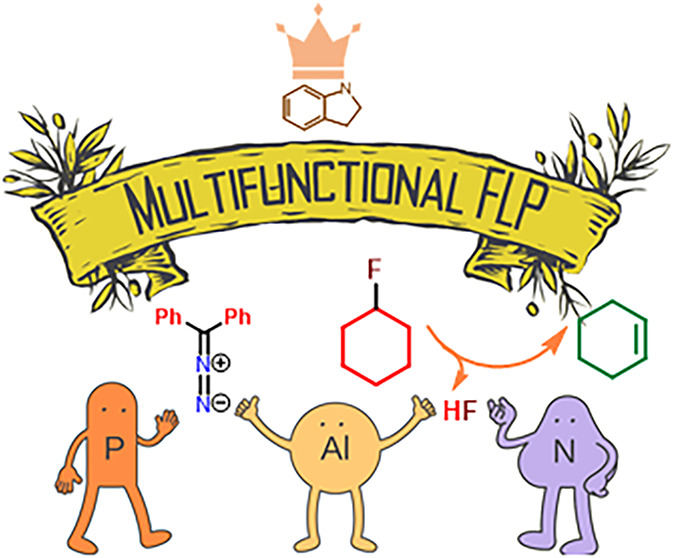

## Introduction

When electron pair donors and acceptors are combined in such a way that, due to steric demands or separating spacer units, no stable dative bonds can form, so-called “frustrated Lewis pairs” (FLPs) are generated. Over the past 20 years, the diversity of FLPs has steadily increased through the use of a wide range of donors and acceptors,^1^ with boranes still being the most common Lewis acids, frequently combined with nitrogen- or phosphorus-based Lewis bases.^2^ Systems based on aluminum as the electrophilic center are much rarer,^3^ although aluminum has a higher intrinsic Lewis acidity,^4^ which in principle should be beneficial for FLP-type reactivity. However, exactly this acidity poses a severe synthetic challenge for accessing *intramolecular* systems, which we are interested in, envisaging steering of the reactivity via the distance and thus the linkers used.^5^ Moreover, we are targeting fluorinated organoaluminum entities, known for being rather strong and hard Lewis acids,^6^ which pose even more problems in the preparation but may allow new type of substrate activations.^7^ In Al-based FLP-chemistry intramolecular systems are rather rare and until recently the known examples only spanned a quite narrow distance range, featuring geminal, vicinal or *peri* arrangements.^8^ Larger spatial separations were unknown before our recent work, utilizing xanthene and biphenylene backbones to construct intramolecular Al/P FLPs (Figure 1).^9^ With the xanthene spacer FLPs were found to be capable of THF-ring opening,^9a,9b^ while the biphenylene system only coordinated THF,^9c^ thus confirming that the reactivity is distance-dependent. All systems investigated were capable of binding CO_2_, and the reversibility of the reaction was determined by the residues at the Al site. The biphenylene system was also able to activate allene.

In all this work, the lability of the Al–C bond repeatedly posed synthetic challenges in constructing the desired FLPs. To ensure that the aluminum atom is firmly anchored to the chosen backbone, we thus contemplated its attachment via heteroatoms. This strategy has so far been pursued only rarely in the literature, as a slight decrease in Lewis acidity must be expected. However, a few examples have been reported for which FLP activity was nonetheless observed (Figure 1).^8d,8f,10^ Particularly in the case of aluminum, the Lewis acidity should remain sufficiently high even upon coordination of a donor atom – especially when fluorinated substituents are bound – so that the advantages of more easily controllable synthetic chemistry clearly outweigh the loss of a small degree of Lewis acidity. We report here about the findings we made pursuing this approach.

**Figure 1:**
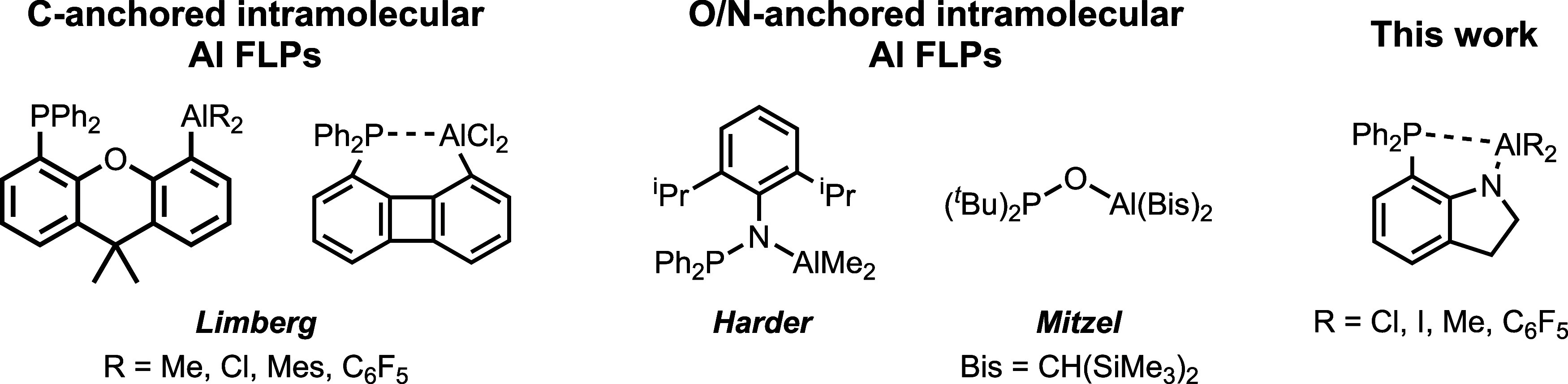
Examples of intramolecular Al based FLPs.

## Results and Discussion

For orienting studies with the background outlined above a backbone based on indoline was chosen, which should lead to a distance between acid and base site of approximately 2.5 Å.

### Synthesis

Starting from indoline a diphenyl phosphine function can be introduced, after protection of the N–H group with (Boc)_2_O, through lithiation with ^*t*^BuLi and subsequent treatment with Ph_2_P­(OPh). Deprotection can then be performed through CF_3_COOH treatment followed by workup to yield Ph_2_P­(Ind)H (Scheme 1).

**Scheme 1:**
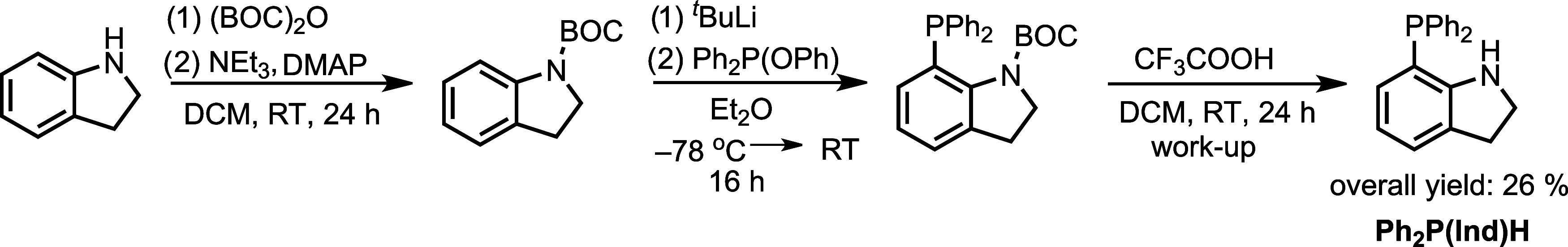
Synthetic Routes to Diphenylphosphine Indoline, **Ph**_**2**_ **P­(Ind)­H**

To elucidate viable metalation pathways starting from Ph_2_P­(Ind)­H, the implementation of aluminum halide moieties {AlX_2_} (X = Cl, I) was pursued (Scheme 2). The synthesis of Ph_2_P­(Ind)­AlCl_2_, **1**, was accomplished via deprotonation of the N–H functionality using EtAlCl_2_. In contrast, the preparation of Ph_2_P­(Ind)­AlI_2_, **2**, required prior deprotonation of the proligand with benzyl potassium to afford Ph_2_P­(Ind)­K, which was subsequently subjected to a salt metathesis reaction with AlI_3_. However, while elemental analysis supported, that the isolated materials corresponded to the targeted compounds with the molar composition Ph_2_P­(Ind)­AlX_2_, NMR spectroscopy as well as single-crystal X-ray diffraction analysis indicated that **1** and **2** are dimers both in the solid state and in solution, with connectivities differing from those expected (see below for details).

**Scheme 2:**
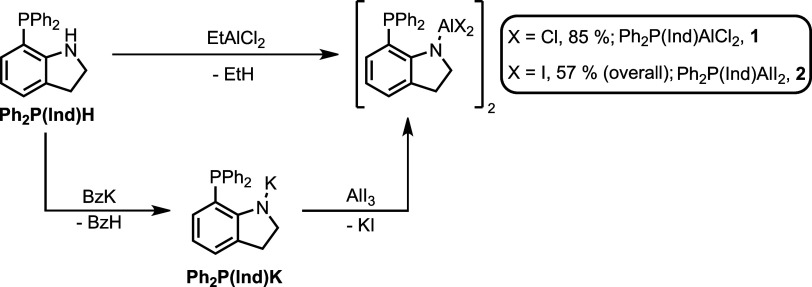
Synthesis of Ph_2_P­(Ind)­AlCl_2_ (**1**) and Ph_2_P­(Ind)­AlI_2_ (**2**)

Building on the established procedures for **1** and **2**, the next objective was the introduction of organoalane functionalities (Scheme 3). Following the deprotonation strategy with alane precursors equipped with sufficiently basic ligands, reaction of Ph_2_P­(Ind)­H and AlMe_3_ cleanly afforded Ph_2_P­(Ind)­AlMe_2_, **3**, accompanied by the elimination of methane. Analogously, Ph_2_P­(Ind)­Al­(C_6_F_5_)_2_, **4**, was obtained upon treatment with MeAl­(C_6_F_5_)_2_. Alternatively, compound **4** can also be synthesized via salt metathesis of Ph_2_P­(Ind)K with (C_6_F_5_)_2_AlCl.

**Scheme 3:**
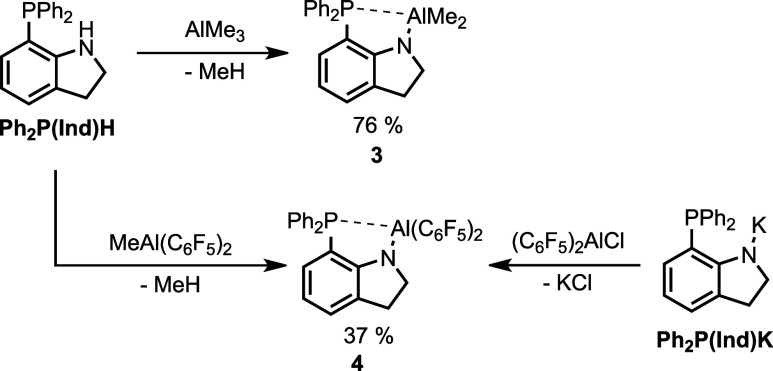
Synthetic Routes to FLPs Ph_2_P­(Ind)­AlMe_2_ (**3**) and Ph_2_P­(Ind)­Al­(C_6_F_5_)_2_ (**4**)

### Structural Investigations

Single-crystals of **1** and **2**·3­(C_6_H_6_) were grown upon concentrating corresponding solutions in aromatic solvents. A single-crystal X-ray structure analysis revealed that the structural configuration of the compounds is more complex than suggested by the formula (Figure 2). They arrange to dinuclear entities, where one aluminum center (Al1) adopts an octahedral coordination sphere, formed by two halide ions two phosphine donors and two N atoms, whereas the second aluminum center (Al2) is surrounded tetrahedrally by two amide and two halide ligands. For **1**, the P···Al distances are 2.5974(8) Å (Al1–P1) and 2.5890(8) Å (Al1–P2). In comparison, slightly shorter P···Al distances are observed for **2**, namely 2.574(3) Å (Al1–P1) and 2.547(3) Å (Al1–P2). In **1** the Al–N bond distances connected with the octahedral aluminum center are relatively longer (2.107(2) Å (Al1–N1) and 2.081(2) Å (Al1–N2)) than those involving the tetrahedral aluminum center 1.907(1) Å (Al2–N1 or Al1–N2). This elongation can be attributed to increased electronic saturation at the octahedral aluminum center arising from phosphine donation, combined with a more crowded coordination sphere. A similar trend in Al–N bond distances is also observed for **2**.

**Figure 2:**
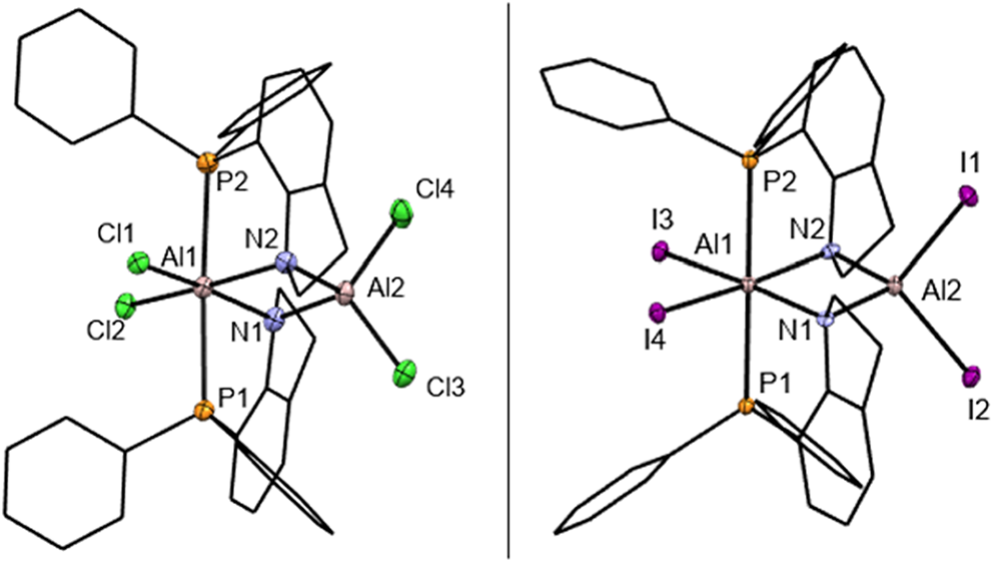
Molecular structures of **1** (left) and **2**·3­(C_6_H_6_) (right). H atoms and cocrystallized solvent molecules are omitted for clarity; thermal ellipsoids are drawn at the 50% probability level. Selected bond lengths [Å] and angles [°]: (for **1**) Al1–N1 2.107(2), Al1–N2 2.081(1), Al2–N1 1.907(1), Al2–N2 1.909(2), Al1–Cl1 2.2260(8), Al1–Cl2 2.2339(7), Al2–Cl3 2.1172(7), Al2–Cl4 2.1137(7); (for **2**) Al1–N1 2.088(5), Al1–N2 2.119(5), Al2–N1 1.922(6), Al2–N2 1.922(5), Al1–I3 2.683(2), Al1–I4 2.672(2), Al2–I1 2.512(2), Al2–I2 2.527(2).

It was possible to crystallize **3** by the layering method using hexane/dichloromethane (3:1) whereas the C_6_F_5_ analogue **4** was crystallized by slow evaporation from dichloromethane. The molecular structures were subsequently determined by X-ray diffraction and in these cases the constitutions and configurations were as expected (Figure 3). Both compounds are monomeric and the P···Al distance of **4** (d­(Al1–P1) = 2.4572(4) Å) is clearly shorter compared to that in **3** (d­(Al1–P1) = 2.515(2) Å), indicating an attractive interaction that as expected is more pronounced in case of the fluorinated residue. Similarly, also the Al–N bond slightly contracts when replacing the Me substituents with C_6_F_5_ groups (**3**: d­(Al1–N1) = 1.879(4) Å; **4:** d­(Al1–N1) = 1.8387(8) Å).

**Figure 3:**
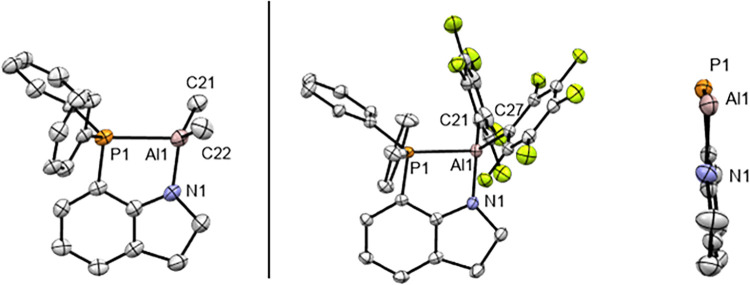
Molecular structures of **3** (left) and **4** (middle) and a side view exemplarily shown for **3** (right). H atoms are omitted for clarity; for the side view substituents of the Al and P centers were also omitted; thermal ellipsoids are drawn at the 50% probability level. Selected bond lengths [Å] and angles [°]: (for **3**) P1–C11 1.789(4), Al1–N1 1.879(4), Al1–C22 1.972(6), C22–Al1–N1 115.1(2), C21–Al1–N1 114.8(2); (for **4**) P1–C11 1.7870(8), Al2–N1 1.8387(8), Al2–C27 1.992(1), Al2–C21 1.986(1), C21–Al2–N1 115.61(4), C27–Al2–N1 116.70(4).

### Solution Behavior

The determined molecular structures of **1** and **2** indicate that the geminal methylene protons of the indoline backbone experience different chemical environments due to the asymmetric dimerization. This is also reflected in the ^1^H NMR spectra of both compounds in noncoordinating solvents, indicating that the dimeric structure is retained upon dissolution. Furthermore, the NMR data revealed the presence of additional species in solution, denoted as **1′** and **2′** (Scheme 4). Based on DOSY and variable-temperature NMR studies, both **1′** and **2′** could be assigned to the corresponding monomeric forms of Ph_2_P­(Ind)­AlX_2_ (see Supporting Information (SI) for details), indicating that compounds **1** and **2** exist in a monomer–dimer equilibrium in noncoordinating solvents. Upon addition of Et_3_PO, the NMR spectra of both compounds displayed only a single set of signals, indicating that coordination of the donor induces dissociation of the dimers to form the corresponding adducts Ph_2_P­(Ind)­AlX_2_(Et_3_PO). In contrast, treatment of **1** with THF instead of Et_3_PO resulted in NMR spectra that displayed two species in solution. Concentration of the THF solution afforded single crystals suitable for X-ray diffraction analysis. The molecular structure revealed the crystalline product to be the scrambling product [Ph_2_P­(Ind)]_2_AlCl­(THF), 1*-THF. These findings indicate that **1** is susceptible to ligand exchange reactions under certain conditions. Although the corresponding scrambling product was not isolated for complex **2**, the iodide derivative is likely similarly prone to dismutation reactions.

**Scheme 4:**
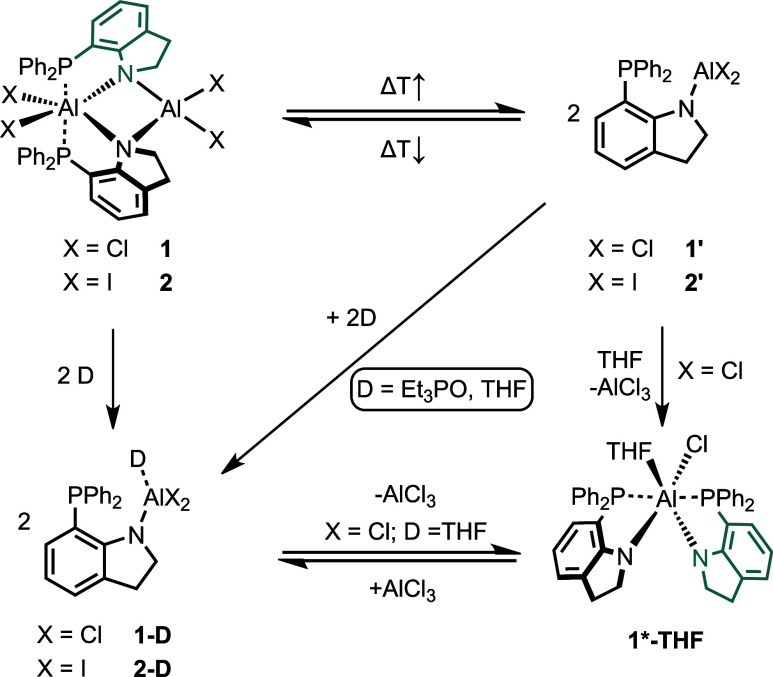
Ligand Scrambling and Monomer/Dimer Equilibrium of Compounds **1** and **2**

While **3** did not show any signs of ligand scrambling in solution, compound **4** does undergo dismutation similar to its halide derivatives, forming the scrambling product [Ph_2_P­(Ind)]_2_Al­(C_6_F_5_), which could be crystallized and identified via X-ray diffraction (see SI for details). The fact that compounds **1**, **2** and **4** are all prone to ligand exchange reactions indicates that the central aluminum center exhibits a strongly ionic bonding character. In contrast, for compound **3** the covalent character of the Al–CH_3_ bonds appears to be sufficiently high to prevent such exchange processes.

These results are somewhat surprising, as the indoline backbone was initially chosen to achieve firm anchoring of the aluminum center via the heteroatom. However, the higher ionic character of the Al–N bond compared to an Al–C bond appears to favor more labile coordination, considering that we had not observed similar ligand exchange phenomena during previous studies using xanthene or biphenylene linkers.^9^ Nevertheless, indoline-based aluminum FLPs can be readily accessed via straightforward synthetic procedures; therefore, we also investigated their potential for the activation of small molecules.

### Properties and Reactivity

Ph_2_P­(Ind)­AlR_2_ with R = Me and C_6_F_5_ are fluorescent, both in solution and in the solid state upon UV excitation (see SI). Roesky and co-workers have observed fluorescence for aluminum complexes of the phosphine-functionalized diamine ligand N,N-bis­(2-(diphenyl-phosphino)­phenyl)­ethane-1,2-diamine and a closer inspection revealed that the photophysical processes likely arise from ligand centered (LC) transitions, which are facilitated and influenced upon coordination of a hard metal by the ligand.^11^ A similar situation can be assumed to be responsible here.

The Gutmann–Beckett acceptor number (AN) was ascertained through the interaction with the neutral compound Et_3_PO. An AN of 57 was determined for **3**, which slightly increased upon introduction of the fluorinated phenyl rings, yielding an AN of 60 for **4**. Notably, the AN of **3** is only slightly lower than the one of AlMe_3_ (AN = 61), indicating only a minor loss of Lewis acidity upon coordination to the heteroatom. In contrast, for **4** the difference compared to Al­(C_6_F_5_)_3_ (AN = 78) is significantly more pronounced.

Having accessed this new type of intramolecular Al/P FLPs their reactivity was examined. While H_2_, olefins and acetylenes did not react, Ph_2_CN_2_ was found to be spanned by the FLPs as 1,1 adducts leading to the formation of Ph_2_P­(Ind)­AlX_2_(Ph_2_CN_2_) (X = Me, **3-CNN**; X = C_6_F_5_, **4-CNN**; see Scheme 5).

**Scheme 5:**
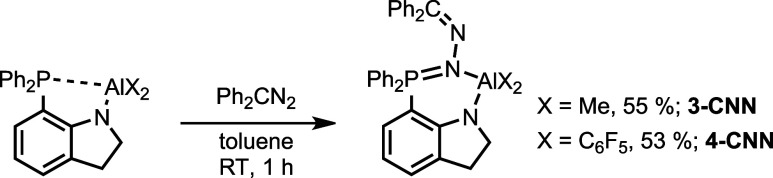
Activation of Diphenyl Diazomethane by FLPs Ph_2_P­(Ind)­AlMe_2_ (**3**) and Ph_2_P­(Ind)­Al­(C_6_F_5_)_2_ (**4**)

Upon mixing, the solution retained its bright yellow color; however, the previously observed fluorescence disappeared completely. Suitable crystals for X-ray analysis were obtained from a saturated benzene solution. The molecular structure revealed that the CNN fragment is bound between the Al/P site via the terminal nitrogen atom (Figure 4). This binding mode is surprising, as the LUMO of such a heterocumulene core would be expected to be primarily centered on the central nitrogen or carbon atom. A pronounced bending of the heterocumulene unit is observed (C–N–N = 117.5(2)° for **3-CNN** and C–N–N = 117.4(2)° for **4-CNN**), indicating significant activation of the substrate. The ^31^P NMR spectrum shows a substantial downfield shift (δ­(^31^P) – 19.39 to 35.29 ppm for **3-CNN**) of the resonance for the phosphine moiety upon substrate coordination, indicating a phosphorus center with the formal oxidation state +V. Furthermore, the N-atom of the indoline backbone displays sp^3^-like character causing the aluminum center to be displaced significantly from the indoline plane (torsion angle 42.5°). This structural change might also account for the loss of fluorescence.

**Figure 4:**
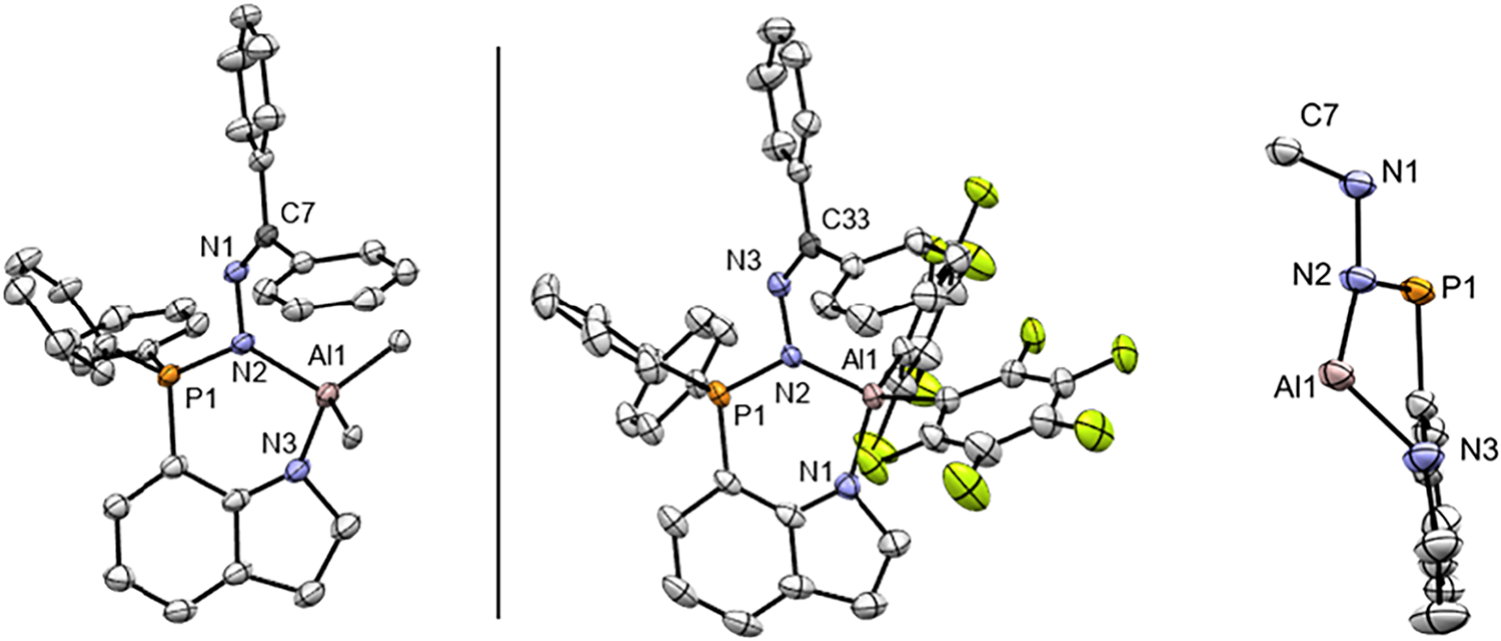
Molecular structures of **3-CNN** (left), **4-CNN** (middle) and a side view exemplarily shown for **3-CNN** (right). H atoms are omitted for clarity; for the side view substituents of the Al and P centers were also omitted. Selected bond lengths [Å] and angles [°]: (for **3-CNN**) P1–N2 1.638(2), Al1–N2 1.927(2), Al1–N3 1.891(3), N1–N2 1.434(3), C7–N1 1.288(3), P1–N2–Al1 123.6(1), C7–N1–N2 117.5(2); (for **4-CNN**) P1–N2 1.646(2), Al1–N2 1.896(2), Al1–N1 1.841(3), N2–N3 1.454(4), C33–N3 1.291(3), P1–N2–Al1 118.8(1), C33–N3–N2 117.4(2).

Notably, Uhl and co-workers reported activation of (Me_3_Si)­HCN_2_ by a P/Al FLP system, exhibiting the same coordination mode as observed in our system.^12^ In contrast, Stephan et al. described the activation of Ph_2_CN_2_ by an oxygen-bridged B/P FLP.^10e^ In their system, a 1,2-addition of the FLP across the CNN fragment was observed, with the terminal nitrogen atom coordinating to the phosphorus center, while the Lewis-acidic boron site binds the central nitrogen atom. The distinct binding mode of the B/P FLP compared to the present system may arise either from geometric constraints imposed by the indoline backbone or from the higher Lewis acidity of the aluminum center.

A benchmark reaction for Al/P FLPs is the one with CO_2_, which was also employed here as a substrate. Exposure of compounds **3** and **4** toward a CO_2_ atmosphere led to decoloration of the reaction mixture and the loss of fluorescence, indicating that both FLPs react with the substrate (Scheme 6). However, X-ray analysis of crystals grown from a saturated benzene solution revealed that CO_2_ did not get trapped in the Al/P reaction space but reacted with the aluminum amide entity via insertion and subsequent dimerization to form [Ph_2_P­(Ind-CO_2_)­AlX_2_]_2_ (X = Me, **3-CO**_**2**_; X = C_6_F_5_, **4-CO**_**2**_; see Figure 5).

**Scheme 6:**
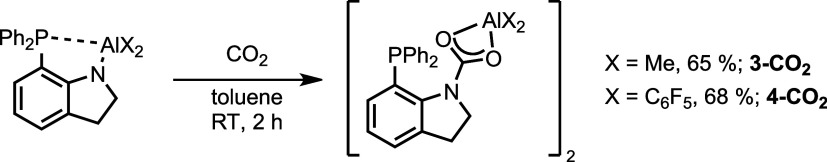
CO_2_ Insertion by Ph_2_P­(Ind)­AlMe_2_ (**3**) and Ph_2_P­(Ind)­Al­(C_6_F_5_)_2_ (**4**)

**Figure 5:**
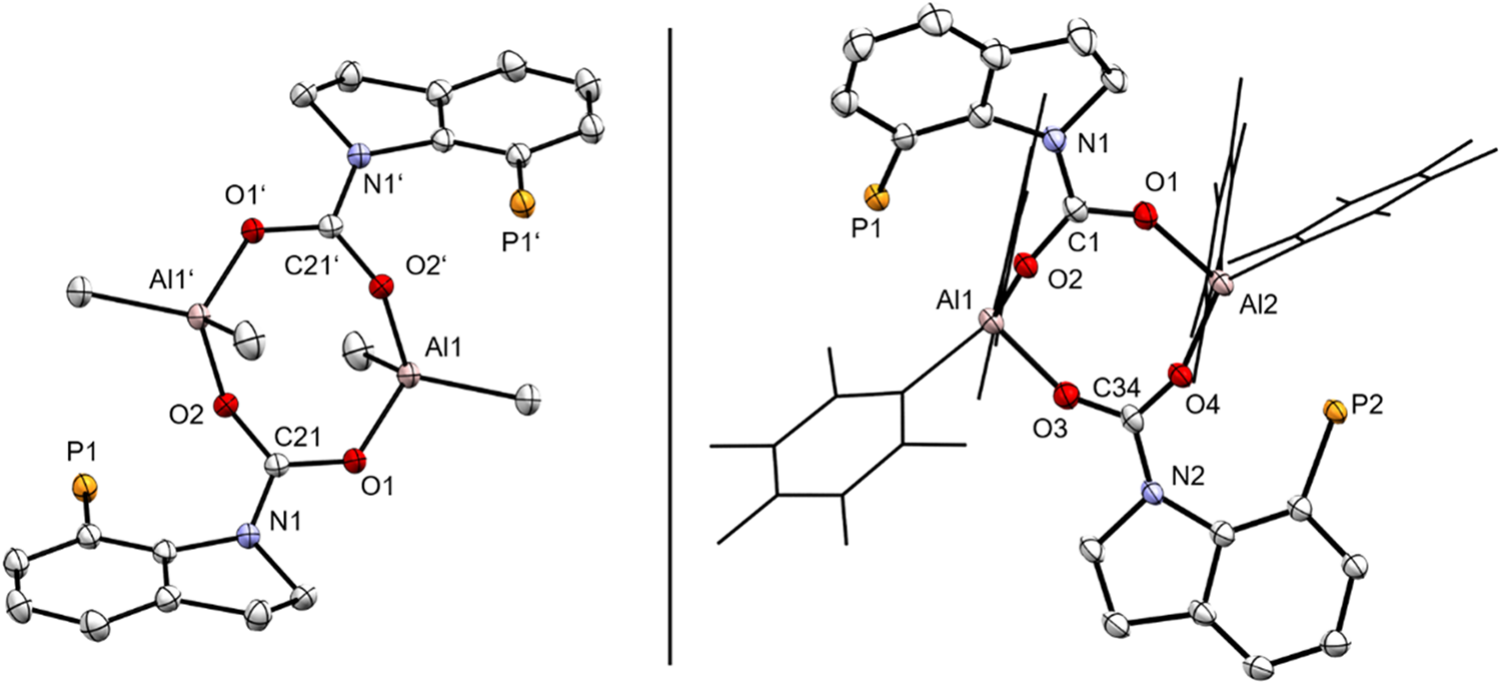
Molecular structures of **3-CO**_**2**_ (left) and **4-CO**_**2**_·7­(C_6_H_6_) (right). H atoms, cocrystallized solvent molecules and PPh_2_ substituents are omitted for clarity (C_6_F_5_) substituents are shown in the wireframe representation; thermal ellipsoids are drawn at the 50% probability level. Selected bond lengths [Å] and angles [°]: (**3-CO**_**2**_) N1–C21 1.335(2), C21–O1 1.227(2), Al1–O1 1.833(1), Al1–O2 1.820(1), O1–C21–O2 122.4(1); (**4-CO**_**2**_) N1–C1 1.335(3), C1–O1 1.281(3), Al1–O2 1.785(2), Al1–O3 1.817(1), O1–C1–O2 123.1(2).

The carbamide bond parameters (C–N length, C–O length, O–C–O angle) in **3-CO**_**2**_ and **4-CO**_**2**_ are nearly identical. However, the Al–O bond distances in **4-CO**_**2**_ [1.785(2) Å (Al1–O2) and 1.817(1) Å (Al1–O3)] are slightly shorter than those observed for **3-CO**_**2**_ [1.833(1) Å (Al1–O1) and 1.820(1) Å (Al1–O2)]. This shortening may be attributed to the higher Lewis acidity of **4**. Notably, neither **3-CO**_**2**_ nor **4-CO**_**2**_ exhibits any Al–P interaction, highlighting that the high oxophilicity of aluminum favors saturation of its coordination sphere through dimerization via the carbamate units.

The results so far indicate that reported compounds Ph_2_P­(Ind)­AlR_2_ feature two reactive sites: The Al···P FLP contact and the aluminum amide function. The latter certainly arises from the nucleophilicity of the amide unit as well as the high ionic character of the Al–N bond which is also reflected by ligand exchange behavior. In this regard, the reported compounds can be considered as multifunctional FLPs. Having established the nature of these reactive sites, we subsequently evaluated the viability of our FLPs toward more challenging substrates featuring C–F bonds.

### Dehydrofluorination of Fluorocyclohexane

An advantage of aluminum-based acid sites is the high affinity to fluorine, and solid aluminum halide compounds have been shown to mediate dehydrofluorinations (DHFs).^13^ Notably, C–F activations by FLPs with other acid sites than Al have been reported.^14^ Hence the behavior of **3** and **4** toward hydrofluorocarbons (HFCs) was tested, using fluorocyclohexane as a model substrate (Scheme 7).

**Scheme 7:**
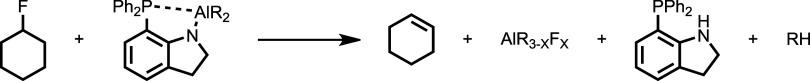
DHF of Fluorocyclohexane Mediated by **3** and **4** (R = Me, C_6_F_5_)

In both cases, quantitative conversion of the substrate to cyclohexene was observed, while the formally eliminated HF was detected in the form of protonated indoline and organoaluminum fluoride species. Additionally, the formation of methane (for **3**) or C_6_F_5_H (for **4**) was observed, although the latter was only detected in trace amounts. Notably, the reaction times required for complete conversion of the substrate differ significantly: for **3**, full conversion was only achieved after 6 h, whereas the reaction time was reduced to 30 min when **4** was used instead. Hence, both FLP compounds can mediate the elimination of HF from HFCs, whereas the increased Lewis acidity of **4** significantly promotes the reactivity.

To elucidate which reactive site of the FLPs initiates the DHF reaction, the nonphosphinated indoline alane (Ind)­AlMe_2_ was tested as a suitable reagent. Indeed, mixing this compound with fluorocyclohexane also resulted in DHF, proving that the phosphine center is not involved in the activation of the C–F bond. Hence, the DHF reaction is not a result of a classical FLP-type activation but is rather just caused by the aluminum center itself in cooperation with its attached ligands. Further test reactions showed that even simple AlMe_3_ is capable of promoting the DHF of fluorocyclohexane. However, our study also revealed that not every type of alane compound is suitable for this transformation: Al­(OtBu)_3_ and Al­(NMe_2_)_3_ did not show any reactivity toward fluorocyclohexane, even after several days.

These results therefore suggest that the DHF reaction requires an Al–X unit with a sufficiently Brønsted-basic ligand X (X = carbanion, amide). Following an initial interaction between the C–F bond and the Lewis acidic Al center, ligand X can then perform the deprotonation step, while the fluoride ion is abstracted by the aluminum center. Recently, Crimmin and co-workers have shown that quite simple bases such as KO^t^Bu and KHMDS are capable of mediating the DHF of activated fluorocarbons.^15^ Although the study covers a broad substrate scope of industrially relevant HFCs, limitations were observed for less activated substrates such as 1-fluorohexane. At this point, aluminum compounds with their high affinity for fluorine may offer a promising alternative: Appropriately designed alane species that combine both a strongly Lewis acidic center and as well as strong Brønsted-basic sites within a single reactive unit might enable the DHF of less activated fluorocarbons that remain challenging for simple base systems.

## Conclusions and Outlook

In summary, we have shown the synthesis of Al/P FLPs with an indoline spacer unit. While the alane functions can be attached to the heteroatom via very straightforward synthetic procedures, the ionic character of the Al–N bond renders the compounds highly prone to ligand exchange reactions. Reactivity studies revealed that the indoline backbone offers two reactive sites, namely the Al···P FLP reaction space and the aluminum amide function, giving rise to multifunctional FLP structures. Which site is triggered during an activation process depends on the nature of the investigated substrate. In future work, we will expand the substrate scope in order to gain further insight into the feasibility of both reactive sites in the field of small molecule activation.

Finally, we have shown that simple alane compounds can be used for the DHF of HFCs in the homogeneous phase. The ability of alane compounds to mediate HF elimination from the substrate depends on both the Lewis acidity of the aluminum center and the Brønsted basicity of its ligands. While simple AlMe_3_ already fulfills both requirements, the DHF capability could potentially be enhanced by designing asymmetric alane compounds of the type AlR_2_^F^X, which combine strongly Brønsted-basic ligands X (e.g., amides or alkyl groups) with fluorinated substituents R^F^ to ensure high Lewis acidity at the central aluminum site. Currently we are designing such aluminum compounds and investigate them across a broader substrate scope.

## Experimental Section

### General Remarks

All experiments were carried out in a dry argon or nitrogen atmosphere using an MBraun glovebox, GS Glovebox Systemtechnik glovebox and/or standard Schlenk techniques. Solvents were purified employing an MBraun Solvent Purification System SPS. Elemental analyses were performed with a HEKA Euro 3000EA elemental analyzer. NMR Spectra were recorded on Bruker NMR spectrometers (Avance II 300 MHz, Avance NEO 300 MHz, Avance 400 MHz, Avance III 500 MHz, Avance 600 MHz). Chemical shifts are referenced to the signal of residual protonated solvent. ATR-infrared (IR) spectra were recorded with a Bruker α FTIR spectrometer and GC-MS were recorded using a *Varian* MS4000 (136).

All materials were obtained from commercial vendors as ACS reagent-grade or better and used as received, if not stated otherwise. N-Boc-indoline,^16^ Al­(C_6_F_5_)_3_·0.5 Tol,^17^ (C_6_F_5_)_2_AlCl,^18^ diphenyldiazomethane^19^ and benzyl potassium^20^ were prepared as described in the literature. 7-diphenylphosphino *N*-Boc indoline^21^ was prepared by modified synthetic procedure described in the literature. A 0.5 M stock solution of MeAl­(C_6_F_5_)_2_ was prepared by stirring a suspension of trimethylaluminum (2 M in toluene, 221 μL, 442 μmol, 1.00 equiv) and Al­(C_6_F_5_)_3_·0.5 Tol (507 mg, 884 μmol, 2.00 equiv) in 2.2 mL toluene for 2 h.

**Caution!** The aluminum compounds bearing pentafluorophenyl substituents prepared in this manuscript are potentially shock and thermally sensitive due to the potential formation of aluminum fluorides and benzyne intermediates. Appropriate care should be taken.

### Synthesis of 7-diphenylphosphino *N*-Boc-indoline

*N*-Boc-indoline (2.00 g, 9.12 mmol, 1.00 equiv) was dissolved in 10 mL dry Et_2_O and cooled to −78 °C. *t-*Butyllithium (1.7 M in pentane, 6.43 mL, 10.94 mmol, 1.20 equiv) was added dropwise and subsequently stirred at −78 °C for 1 h. A solution of phenoxydiphenylphosphine (3.05 g, 10.94 mmol, 1.20 equiv) in 10 mL of dry Et_2_O was added and stirred for another hour at −78 °C. The cooling bath was removed and the yellowish reaction mixture was stirred at room temperature for 16 h. Excessive *t*-BuLi was quenched with 20 mL of saturated NH_4_Cl solution and the mixture was extracted with DCM (3 × 20 mL). The combined organic phases were dried over Na_2_SO_4_ and concentrated. The crude product was recrystallized from hexane (∼30 mL). This yielded the product as off-white solid (1.58 g, 3.92 mmol, 42%). Crystals suitable for X-ray diffraction analysis were grown via slow evaporation of a dichloromethane solution. ^1^H NMR (300 MHz, CDCl_3_, 298 K): δ [ppm] = 7.42–7.37 (m, 4H, *o*-PPh_2_), 7.30–7.27 (m, 6H, *m*-PPh_2_ and *p*-PPh_2_), 7.12 (dd, ^*3*^ *J*_*HH*_ = 7.3 Hz, ^*4*^ *J*_*HH*_ = 1.3 Hz, H_a_), 6.89 (t, ^*3*^ *J*_*HH*_ = 7.5 Hz, 1H, H_b_), 6.77 (ddd, ^*3*^ *J*_*HH*_ = 7.9 Hz, ^*3*^ *J*_*HH*_ = 5.1 Hz, ^*4*^ *J*_*HP*_ = 1.3 Hz 1H, H_c_), 3.94 (t, ^*3*^ *J*_*HH*_ = 7.9 Hz, 2H, H_e_), 3.02 (t, ^*3*^ *J*_*HH*_ = 7.9 Hz, 2H, H_d_), 1.33 (s, 9H, ^*t*^Bu). ^13^C­{^1^H} NMR (75 MHz, CDCl_3_, 298 K): δ [ppm] = 152.81 (d, ^4^ *J*CP = 3 Hz, C=O), 145.2 (d, ^2^ *J*CP = 15 Hz, ind-*C*-NCH2), 138.59 (d, ^1^ *J*CP = 13.5 Hz, PhP-*C*-C_5_H_5_), 134.22 (d, ^2^ *J*CP = 21.75 Hz, *C*-H_o_), 133.92 (d, ^2^ *J*CP = 3 Hz, *C*-H_a_), 133 (d, ^3^ *J*CP = 4.5 Hz, ind-*C*-CH_2_CH_2_), 128.45 (s, *C*- H_m_/ H_p_), 128.17 (s, H_m_/ H_p_), 126.85 (s, *C*-H_c_), 124.19 (s, *C*-H_b_), 123.82 (s, *C*-PPh_2_), 80.60 (s, C­(CH_3_)_3_), 49.76 (s, *C*-H_e_), 29.29 (s, *C*-H_d_), 28.28 (s, CH_3_). ^31^P­{^1^H} NMR (121 MHz, CDCl_3_, 298 K): δ [ppm] = −8.74 (s). Anal. Calcd. for C_25_H_26_NO_2_P: C, 74.42; H, 6.50; N, 3.47. Found: C, 74.47; H, 6.41; N, 3.47.

### Synthesis of 7-diphenylphosphino Indoline, Ph_2_P­(Ind)­H

7-diphenylphosphino *N*-Boc-indoline (1.00 g, 2.48 mmol) was dissolved in 10 mL of DCM. After addition of CF_3_COOH (6 mL) the reaction mixture was stirred at room temperature for 24 h. Then saturated aqueous NaHCO_3_ solution was added until no more gas evolution was observed. Subsequently, the mixture was extracted with CH_2_Cl_2_ (3 × 20 mL). The organic phase was dried over Na_2_SO_4_ and the solvent was removed under vacuum. The crude sticky product was recrystallized from hexane to give the pure desired compound (547 mg, 1.8 mmol, 72%) as white powder. Crystals suitable for X-ray diffraction analysis were grown via slow evaporation of a dichloromethane solution. ^1^H NMR (300 MHz, C_6_D_6_, 298 K): δ [ppm] = 7.52–7.45 (m, 4H, *o*-PPh_2_), 7.10–7.01 (m, 6H, *m*-PPh_2_ and *p*-PPh_2_), 6.98–6.94 (m, 2H, H_a_, H_c_), 6.62 (t, ^*3*^ *J*_*HH*_ = 7.4 Hz, 1H, H_b_), 3.94 (s, 1H, NH), 2.90 (t, ^*3*^ *J*_*HH*_ = 8.4 Hz, 2H, H_e_), 2.60 (t, ^*3*^ *J*_*HH*_ = 8.4 Hz, 2H, H_d_). ^13^C­{^1^H} NMR (75 MHz, C_6_D_6_, 298 K): δ [ppm] 156.08 (d, ^2^ *J*CP = 18.4 Hz, ind-*C*-NCH2), 136.95 (d, ^1^ *J*CP = 10.2 Hz, PhP-*C*-C_5_H_5_), 134.01 (d, ^2^ *J*CP = 19.1 Hz, *C*-H_o_), 132.47 (d, ^2^ *J*CP = 6.6 Hz, *C*-H_a_), 128.90 (s, ind-*C*-CH_2_CH_2_), 128.81 (s, *C*-H_m_/ H_p_), 128.79 (s, *C*- H_m_/ H_p_), 125.49 (s, *C*-H_c_), 118.81 (d, ^3^ *J*CP = 3.4 Hz, *C*-H_b_), 114.37 (d, ^1^ *J*CP = 9.6 Hz, *C*-PPh_2_), 46.96 (s, *C*-H_e_), 29.72 (d, ^4^ *J*CP = 2.21 Hz, *C*-H_d_). ^31^P­{^1^H} NMR (121 MHz, C_6_D_6_, 298 K): δ [ppm] = −17.75 (s). Anal. Calcd. for: C_20_H_18_NP: C, 79.19; H, 5.98; N, 4.62. Found: C, 79.01; H, 5.83; N, 4.61.

### Synthesis of Ph_2_P­(Ind)­K

Benzyl potassium (944 mg, 7.25 mmol, 1.10 equiv) was added at room temperature to a solution of Ph_2_P­(Ind)H (2.00 g, 6.59 mmol, 1.00 equiv) in toluene (15 mL). The yellow solution was stirred for 2 h at room temperature. The yellow precipitate was washed with hexane and dried under high *vacuo* for 4 h to almost quantitatively yield the bright yellow compound Ph_2_P­(Ind)K (1.8 g, 5.27 mmol, 80%). Crystals suitable for X-ray diffraction analysis were grown via slow evaporation of a toluene solution. ^1^H NMR (300 MHz, THF-d_8_, 298 K): δ [ppm] = 7.35–7.30 (m, 4H, *o*-PPh_2_), 7.27–7.20 (m, 6H, *m*-PPh_2_ and *p*-PPh_2_), 6.46 (d, ^*3*^ *J*_*HH*_ = 7.4 Hz, 1H, H_a_), 5.88 (t, ^*3*^ *J*_*HH*_ = 7.1 Hz, 1H, H_c_), 5.37 (t, ^*3*^ *J*_*HH*_ = 7.1 Hz, 1H, H_b_), 3.65 (t, ^*3*^ *J*_*HH*_ = 8.8 Hz, 2H, H_e_), 2.76 (t, ^*3*^ *J*_*HH*_ = 8.8 Hz, 2H, H_d_). ^13^C­{^1^H} NMR (75 MHz, THF-d_8_, 298 K): δ [ppm] 140.84 (d, ^2^ *J*CP = 9.75 Hz, ind-*C*-NCH_2_), 134.96 (d, ^1^ *J*CP = 18 Hz, PhP-*C*-C_5_H_5_), 132.49 (s, *C*-H_o_), 131.73 (d, ^2^ *J*CP = 5.25 Hz, *C*-H_a_), 129.84 (s, ind-*C*-CH_2_CH_2_), 128.87 (s, *C*- H_m_/ H_p_), 128.38 (s, *C*- H_m_/ H_p_), 126.20 (s, *C*-H_c_), 123.26 (s, *C*-H_b_), 105.58 (s, *C*-PPh_2_), 55.69 (s, *C*-H_e_), 32.75 (s, *C*-H_d_). ^31^P­{^1^H} NMR (121 MHz, THF-d_8_, 298 K): δ [ppm] = −17.29 (s). Anal. Calcd. for [C_20_H_17_KNP]_n_: C, 70.36; H, 5.02; N, 4.10. Found: C, 68.39; H, 4.94; N, 3.51.

### Synthesis of (Ind)­AlMe_2_

Indoline (1.00 g, 8.39 mmol, 1.00 equiv) was dissolved in 10 mL of dry toluene and AlMe_3_ (4.19 mL, 2.00 M in toluene, 8.39 mmol, 1.00 equiv) was added slowly. After stirring for 2 h at room temperature the solvent was removed *in vacuo* and the residue was washed with 5 mL of dry hexane. The obtained colorless solid material was dried in high *vacuo* for 2 h to yield (Ind)­AlMe_2_ (980 mg, 5.59 mmol, 66%). Crystals suitable for X-ray diffraction analysis were obtained by recrystallization from a boiling hexane solution. **Note:** This compound undergoes *cis/trans* isomerism in solution (see SI for more information). ^1^H NMR (*trans* isomer, 300 MHz, C_6_D_6_, 298 K): δ [ppm] = 7.24 (d, 1 H, H_Ar_), 7.02–6.99 (m, 2 H, H_Ar_), 6.90–6.84 (m, overlapping with signal of *cis* isomer, 1 H, H_Ar_), 3.41 (t, ^*3*^ *J*_*HH*_ = 7.7 Hz, 2 H, N*CH*_*2*_CH_2_), 2.54 (t, overlapping with signal of *cis* isomer, 2 H, NCH_2_ *CH*_*2*_), −0.51 (s, 6 H, AlMe_2_). ^1^H NMR (*cis* isomer, 300 MHz, C_6_D_6_, 298 K): δ [ppm] = 7.44 (d, 1 H, H_Ar_), 7.08–7.04 (m, 2 H, H_Ar_), 6.90–6.84 (m, overlapping with signal of *cis* isomer, 1H, H_Ar_), 3.32 (t, ^*3*^ *J*_*HH*_ = 7.7 Hz, 2 H, N*CH*_*2*_CH_2_), 2.54 (t, overlapping with signal of *cis* isomer, 2 H, NCH_2_ *CH*_*2*_), −0.37 (s, 3 H, AlMe_2_), −0.57 (s, 3 H, AlMe_2_). Anal. Calcd. for [C_10_H_14_AlN]_2_: C, 68.55; H, 8.05; N, 7.99. Found: C, 67.27; H, 7.77; N, 7.88.

### Synthesis of [Ph_2_P­(Ind)­AlCl_2_]_2_, 1

EtAlCl_2_ (1.00 mL, 1.00 mmol, 1 M in hexane, 1.00 equiv) was added at room temperature to a suspension of Ph_2_P­(Ind)H (303 mg, 1.00 mmol, 1.00 equiv), in DCM (3 mL). After refluxing the bright yellow solution for 1 h the volume was reduced to half. Storing at −30 °C afforded [Ph_2_P­(Ind)­AlCl_2_]_2_, **1**, as a colorless crystalline material. Further concentration and storage at −30 °C afforded another batch of crystals (combined yield: 340 mg, 0.85 mmol, 85%). Single-crystals suitable for X-ray diffraction analysis were grown via slow evaporation of the volatiles of a DCM/toluene mixture. **Note:** This compound features a monomer–dimer equilibrium in solution (see SI for more information). ^1^H NMR (600 MHz, CDCl_3_, 298 K): δ [ppm] = 7.93 (t, 4 H, H_PPh2_), 7.81 (t, 4 H, H_PPh2_), 7.50–7.10 (overlapping multiplets of **1** and **1** ^**’**^, 18 H, H_Ar/PPh2_), 3.63 (m, 2 H, N*CH*_*2*_CH_2_), 2.97 (m, 2 H, N*CH*_*2*_CH_2_), 2.81 (m, 2 H, NCH_*2*_ *CH*_*2*_), 2.66 (m, 2 H, NCH_*2*_ *CH*_*2*_). ^31^P­{^1^H} NMR (243 MHz, CDCl_3_, 298 K): δ [ppm] = −27.00 (bs). Anal. Calcd. for: [C_40_H_34_Al_2_Cl_4_N_2_P_2_]_2_·(C_7_H_8_)_2_·CH_2_Cl_2_: C, 61.02; H, 4.64; N, 3.00. Found: C, 60.75; H, 4.64; N, 2.83.

### Synthesis of [Ph_2_P­(Ind)]_2_AlCl­(THF), 1*-THF

Compound **1** (30 mg, 38 μmmol) was dissolved in THF (1 mL). After stirring for 1 min, the solution was concentrated to approximately one-third of its original volume. The resulting mixture was left standing overnight, affording yellow crystals of 1*-THF. The crystals were collected by filtration, washed with hexane, and dried under reduced pressure (11 mg, 15 μmol, 40%). The isolated crystalline material was suitable for X-ray diffraction analysis. ^1^H NMR (500 MHz, THF-*d*_*8*_, 298 K): δ [ppm] = 7.40 (m, 4 H, *o*-PPh_2_), 7.29 (m, 1 H, H_Ar_), 7.20 (m, 1 H, H_Ar_), 7.07 (m, 6 H, *m*-PPh_2_), 6.72 (m, 2 H, *p*-PPh_2_), 6.15 (m, 1 H, H_Ar_), 3.48 (t, ^*3*^ *J*_*HH*_ = 9.0 Hz, 2 H, N*CH*_*2*_CH_2_), 2.56 (t, ^*3*^ *J*_*HH*_ = 9.0 Hz, 2 H, NCH_*2*_ *CH*_*2*_). ^13^C­{^1^H} NMR (101 MHz, C_6_D_6_, 298 K): δ [ppm] = 167.47 (br, ind-*C*-NCH_2_), 135.75 (b, PhP-*C*-PPh_2_), 134.32 (br,*C*_*o*_-PPh_2_), 132.85­(*s*, *C*_*o*_-ind), 132.72­(s, ind-*C*-CH_2_CH_2_), 129.40 (s, *C*_*m*_ */C*_*p*_-PPh_2_), 129.31 (br, *C*_*m*_ */C*_*p*_-PPh_2_), 128.89 (br, *C*_*m*_-ind), 126.63 (s, *C*_*p*_-ind), 114.01 (br, *C*-PPh_2_), 50.01 (s, N*CH*_*2*_), 31.45 (br, N*CH*_*2*_CH_2_). ^31^P­{^1^H} NMR (202 MHz, THF-*d*_*8*_, 298 K): δ [ppm] = −29.74 (s). Anal. Calcd. for C_40_H_34_Al_2_I_4_N_2_P_2_·(C_4_H_8_O)_2_: C, 70.70; H, 6.62; N, 3.17. Found: C, 68.68; H, 6.47; N, 2.97.

### Synthesis of [Ph_2_P­(Ind)­AlI_2_]_2_, 2

AlI_3_ (236 mg, 0.58 mmol, 1.00 equiv) and Ph_2_P­(Ind)K (200 mg, 0.58 mmol, 1.00 equiv) were suspended in toluene (10 mL) and stirred for 16 h at room temperature. After filtration and further extraction of the solid residue with toluene (5 mL) the combined organic phases were concentrated in vacuo to obtain a bright yellow oil. The residue was extracted with a DCM/hexane mixture. Concentration in *vacuo* led to the precipitation of a yellow solid (245 mg, 0.42 mmol, 72%) which was filtered off and dried under reduced pressure. Recrystallization via slow evaporation of a DCM/toluene mixture yielded [Ph_2_(Ind)­AlI_2_]_2_, **2**, as a colorless crystalline material. Crystals suitable for X-ray diffraction analysis were grown from a concentrated toluene solution. **Note:** This compound monomer–dimer equilibrium in solution (see SI for more information). ^1^H NMR (500 MHz, DCM-*d*_*2*_, 298 K): δ [ppm] = 8.39 (t, 4 H, H_PPh2_), 7.78 (t, 4 H, H_PPh2_), 7.64–7.24 (overlapping multiplets of **1** and **1** ^**’**^, 18 H, H_Ar/PPh2_), 4.13 (m, 2 H, N*CH*_*2*_CH_2_), 3.35 (m, 2 H, N*CH*_*2*_CH_2_), 2.99 (m, 2 H, NCH_*2*_ *CH*_*2*_), 2.84 (m, 2 H, NCH_*2*_ *CH*_*2*_). ^31^P­{^1^H} NMR (202 MHz, C_6_D_6_, 298 K): δ [ppm] = −35.18 (bs). Anal. Calcd. for C_40_H_34_Al_2_I_4_N_2_P_2_: C, 41.20; H, 2.94; N, 2.40. Found: C, 41.20; H, 2.81; N, 2.09.

### Synthesis of Ph_2_P­(Ind)­AlMe_2_, 3

Ph_2_P­(Ind)H (1.00 g, 3.29 mmol, 1.00 equiv) was dissolved in toluene (10 mL) and AlMe_3_ (1.64 mL, 2.00 M in toluene, 3.29 mmol, 1.00 equiv) was added slowly. The reaction mixture immediately turned lime green and was stirred for 2 h at room temperature. The solvent was removed *in vacuo*. The residue was washed with 5 mL of hexane and dried under high *vacuo* for 1 h to yield Ph_2_P­(Ind)­AlMe_2_, **3**, (895 mg, 2.49 mmol, 76%) as a lime green compound. Crystals suitable for X-ray diffraction analysis were grown by layering a concentrated DCM solution with a 3-fold excess of hexane. ^1^H NMR (300 MHz, C_6_D_6_, 298 K): δ [ppm] = 7.43–7.36 (m, 4H, *o*-PPh_2_), 6.96–6.91 (m, 6H, *m*-PPh_2_ and *p*-PPh_2_), 6.90–6.83 (m, 2H, H_a_, H_c_), 6.34 (ddd, ^*3*^ *J*_*HH*_ = 8.1 Hz, ^*3*^ *J*_*HH*_ = 6.8 Hz, ^*4*^ *J*_*HP*_ = 1.5 Hz 1H, H_b_), 3.62 (t, ^*3*^ *J*_*HH*_ = 8.8 Hz, 2H, H_e_), 2.88 (t, ^*3*^ *J*_*HH*_ = 8.8 Hz, 2H, H_d_), −0.20 (d, ^*3*^ *J*_*HP*_ = 4.1 Hz, 6 H, AlMe_2_). ^13^C­{^1^H} NMR (75 MHz, C_6_D_6_, 298 K): δ [ppm] = 168.47 (d, ^2^ *J*CP = 14.25 Hz, ind-*C*-NCH2), 133.31 (d, ^1^ *J*CP = 7.5 Hz, PhP-*C*-C_5_H_5_), 131.47 (d, ^2^ *J*CP = 11.25 Hz, *C*-H_o_), 130.74 (*C*- H_a_), 130.46 (s, ind-*C*-CH_2_CH_2_), 129.62 (s, *C*- H_m_/ H_p_), 129.14 (s, *C*- H_m_/ H_p_), 129.06 (s, *C*-H_c_), 114.54 (d, ^3^ *J*CP = 3.75 Hz, *C*-PPh_2_), 98.65 (d, ^1^ *J*CP = 25.75 Hz, *C*-H_b_), 48.42 (d, ^4^ *J*CP = 2.21 Hz, *C*-H_e_), 31.84 (d, ^4^ *J*CP = 2.25 Hz, *C*-H_d_), −9.97 (d, ^*2*^ *J*_*CP*_ = 11.25 Hz, AlMe_2_). ^31^P­{^1^H} NMR (121 MHz, C_6_D_6_, 298 K): δ [ppm] = −19.39 (s). Anal. Calcd. for C_22_H_23_AlNP: C, 73.53; H, 6.45; N, 3.90. Found: C, 72.99; H, 6.55; N, 3.92.

### Synthesis of Ph_2_P­(Ind)­Al­(C_6_F_5_)_2_, 4

*Method A*: Ph_2_P­(Ind)K (177 g, 0.52 mmol, 1.00 equiv) and (C_6_F_5_)_2_AlCl (205 mg, 0.52 mmol, 1.00 equiv) were suspended in toluene (5 mL) and stirred overnight. After removal of the solvent the residue was extracted with toluene (3x 5 mL). The combined organic phases were concentrated to about 2 mL and stored for crystallization overnight. The bright yellow crystalline material was filtered off, washed with hexane and dried under reduced pressure to afford Ph_2_P­(Ind)­Al­(C_6_F_5_)_2_, **4** (124 mg, 0.19 mmol, 37%). *Method B*: MeAl­(C_6_F_5_)_2_ (0.2 mL, 0.5 M in toluene, 0.01 mmol, 1.00 equiv) was added to a toluene solution of Ph_2_P­(Ind)H (30 mg, 0.1 mmol, 1.00 equiv) and the mixture was refluxed for 2 days. After removal of the solvent the bright yellow residue was washed with small portions of hexane, dried under reduced pressure and was used without any further purification. Crystals suitable for X-ray diffraction analysis were grown via slow evaporation of a benzene solution. **Note:** This compound undergoes ligand scrambling in solution (see SI for more information). ^1^H NMR (400 MHz, C_6_D_6_, 298 K): δ [ppm] = 7.26 (m, 4 H, H_PPh2_), 7.08–6.75 (overlapping multiplets of **4** and **4** ^**’**^, 22 H, H_Ar/PPh2_), 6.72 (m, 1 H, H_Ar_), 6.36 (m, 1 H, H_Ar_), 3.89 (m, 2 H, N*CH*_*2*_CH_2_), 2.95 (overlapping multiplets of **4** and **4** ^**’**^, 2 H, N*CH*_*2*_CH_2_). ^31^P­{^1^H} NMR (162 MHz, C_6_D_6_, 298 K): δ [ppm] = −19.61 (bs). ^19^F NMR (376 MHz, C_6_D_6_, 298 K): δ [ppm] = −121.06 (m, 4 F, *o*-F), −152.27 (t, ^*3*^ *J*_*FF*_ = 19.7 Hz, 2 F, *p*-F), −161.10 (m, 4 F, *m*-F). Anal. Calcd. for C_32_H_17_AlF_10_NP: C, 57.93; H, 2.58; N, 2.11. Found: C, 58.90; H, 2.69; N, 2.14.

### Synthesis of Ph_2_P­(Ind)­Al­(Me)_2_(Ph_2_CN_2_), 3-CNN

Ph_2_P­(Ind)­AlMe_2_ (100 mg, 0.28 mmol, 1.00 equiv) was dissolved in toluene (5 mL). A solution of diphenyldiazomethane (54 mg, 0.28 mmol, 1.00 equiv) in 3 mL toluene was added slowly and stirred for 1 h at room temperature. The solvent was removed *in vacuo*. The residue was washed with 5 mL of hexane and dried under high *vacuo* for 2 h to yield Ph_2_P­(Ind)­Al­(Me)_2_(Ph_2_CN_2_), **3-CNN**, (85 mg, 0.15 mmol, 55%) as a yellow powder. Crystals suitable for X-ray diffraction analysis were grown from concentrated benzene solution. ^1^H NMR (300 MHz, C_6_D_6_, 298 K): δ [ppm] = 7.88–7.80 (m, 4H, *o*-PPh_2_), 7.48–7.46 (m, 2H, *p*-PPh_2_), 7.25–7.18 (m, 4H, *m*-PPh_2_), 7.02–6.93 (m, 10H, *C*-PPh_2_), 6.84–6.82 (m, 1H, H_a_), 6.65–6.58 (m, 1H, H_c_), 6.22–6.16 (m, 1H, H_b_), 3.66 (t, ^*3*^ *J*_*HH*_ = 8.9 Hz, 2H, H_e_), 2.61 (t, ^*3*^ *J*_*HH*_ = 8.8 Hz, 2H, H_d_), −0.69 (br, 6 H, AlMe_2_). ^13^C­{^1^H} NMR (75 MHz, C_6_D_6_, 298 K): δ [ppm] = 165.01 (d, ^*4*^ *J*_*CP*_ = 5.25 Hz, *ipso* carbon attached to C=N), 162.6 (d, ^2^ *J*_*CP*_ = 22.5 Hz, ind-*C*-NCH_2_), 140.52 (br, PhP-*C*-C_5_H_5_), 134.92 (d, ^2^ *J*_*CP*_ = 7.5 Hz, *C*-H_o_), 134.12 (d, ^2^ *J*_*CP*_ = 9 Hz, *C*- H_a_), 132.39 (d, ^3^ *J*_*CP*_ = 9 Hz, ind-*C*-CH_2_CH_2_), 131.15 (s, *C*- H_m_/ H_p_), 130.05 (s, *C*- H_m_/ H_p_), 129.90 (s, *C*-H_c_), 129.56 (s, *C*-PPh_2_), 129.23 (s, *C*_*o/m/p‑CPh2*_), 128.81 (s, *C*_*o/m/p −CPh2*_) 128.75 (s, *C*_*o/m/p −CPh2*_), 112.61 (d, ^1^ *J*CP = 12.75 Hz, *C*-H_b_), 89.84­(s, N = CPh_2_), 50.01 (s, *C*-H_e_), 29.27 (s, *C*-H_d_), −9.62 (br, AlMe_2_). ^31^P­{^1^H} NMR (121 MHz, C_6_D_6_, 298 K): δ [ppm] = 35.29. Anal. Calcd. for C_35_H_33_AlN_3_P: C, 73.93; H, 6.01; N, 7.59. Found: C, 75.02; H, 5.90; N, 6.86. Due to instability of the activated azo substrate the values always deviated somewhat more than usually acceptable.

### Synthesis of Ph_2_P­(Ind)­Al­(C_6_F_5_)_2_(Ph_2_CN_2_), 4-CNN

Compound **4** was generated in situ by mixing Ph_2_P­(Ind)H (50 mg, 0.15 mmol, 1.00 equiv) and MeAl­(C_6_F_5_)_2_ (0.3 mL, 0.5 M in toluene, 0.15 mmol, 1.00 equiv) in toluene (2 mL). After refluxing for 2 days a solution of diphenyldiazomethane (29 mg, 0.15 mmol, 1.00 equiv) in 3 mL toluene was added slowly and the mixture was stirred for an additional hour at room temperature. After removal of the solvent the oily residue was extracted with hexane (2 mL) and stirred overnight. This caused precipitation of Ph_2_P­(Ind)­Al­(C_6_F_5_)_2_(Ph_2_CN_2_), **4-CNN** (68 mg, 0.08 mmol, 53%) as a colorless solid which was washed with hexane and dried under reduced pressure. Crystals suitable for X-ray diffraction analysis were grown by layering a concentrated benzene solution with hexane. ^1^H NMR (400 MHz, C_6_D_6_, 298 K): δ [ppm] = 7.72 (m, 4H, *o*-PPh_2_), 7.22 (m, 2H, *p*-PPh_2_), 6.93–6.76 (m, 16 H, *m*-PPh_2_/H_Ar_), 6.29 (m, 1H, H_Ar_), 3.46 (t, ^*3*^ *J*_*HH*_ = 8.9 Hz, 2H, H_e_), 2.63 (t, ^*3*^ *J*_*HH*_ = 8.8 Hz, 2H, H_d_). ^31^P­{^1^H} NMR (162 MHz, C_6_D_6_, 298 K): δ [ppm] = 38.98 (s). ^19^F NMR (376 MHz, C_6_D_6_, 298 K): δ [ppm] = −120.13 (m, 4 F, *o*-F), −155.64 (t, ^*3*^ *J*_*FF*_ = = 19.7 Hz, 2 F, *p*-F), −162.78 (m, 4 F, *m*-F). Anal. Calcd. for: C_45_H_27_AlF_10_N_3_P: C, 63.02; H, 3.17; N, 4.90. Found: C, 63.68; H, 3.75; N, 3.18. Due to instability of the activated azo substrate the values always deviated somewhat more than usually acceptable.

### Synthesis of Ph_2_P­(Ind-CO_2_)­Al­(Me)_2_, 3-CO_2_

A benzene solution (1 mL) of compound **3** (60 mg, 0.17 mmol) was subjected to three cycles of freeze–pump–thaw degassing. CO_2_ was added (approximately 2 bar) and the mixture was stirred for 2 h causing a complete discoloration of the solution. After removal of the solvent the oily residue was taken up in hexane (1 mL) and stirred overnight. This caused precipitation of [Ph_2_P­(Ind-CO_2_)­Al­(Me)]_2_, **3-CO**_**2**_ (44 mg, 0.11 mmol, 65%) as a colorless solid which was washed with hexane and dried under reduced pressure. Crystals suitable for X-ray diffraction analysis were grown from a concentrated benzene solution. ^1^H NMR (500 MHz, C_6_D_6_, 298 K): δ [ppm] = 7.49 (m, 4H, *o*-PPh_2_), 7.08–7.02 (m, 6H, *m*-PPh_2_ and *p*-PPh_2_), 6.97 (m, 1H, ^Ar^H), 6.70–6.66 (m, 2H, ^Ar^H), 3.61 (t, ^*3*^ *J*_*HH*_ = 7.4 Hz, 2H, CH_2_), 2.18 (br, 2H, CH_2_), −0.20 (br, 6 H, AlMe_2_). ^13^C­{^1^H} NMR (125 MHz, C_6_D_6_, 298 K): δ [ppm] = 155.84 (br, ind-*C*-NCH2), 143.3 (d, ^1^ *J*CP = 18.75 Hz, PhP-*C*-C_5_H_5_), 137.52 (d, ^2^ *J*CP = 18.75 Hz, *C*-H_o_), 134.79 (d, ^2^ *J*CP = 28.75 Hz, *C*-H_a_), 132.62 (d, ^*3*^ *J*_*CP*_ = 6.25 Hz, ind-*C*-CH_2_CH_2_), 130.60 (s, *C*- H_m_/ H_p_), 129.35 (s, *C*- H_m_/ H_p_), 128.77 (d, ^*1*^ *J*_*CP*_ = 8.75 Hz, *C*-PPh_2_), 125.84 (s, *C*-H_c_), 124.45 (s, *C*-H_b_), 51.25 (*C*-H_e_), 28.82 (*C*-H_d_), −8.73 (br, Al*Me*_*2*_). ^31^P­{^1^H} NMR (202 MHz, C_6_D_6_, 298 K): δ [ppm] = −7.56 (s). Anal. Calcd. for [C_23_H_24_AlNO_2_P]_2_: C, 68.31; H, 5.98; N, 3.46. Found: C, 68.77; H, 5.83; N, 3.41.

### Synthesis of Ph_2_P­(Ind)­Al­(C_6_F_5_)_2_CO_2_, 4-CO_2_

Compound **4** was generated in situ by mixing Ph_2_P­(Ind)H (50 mg, 0.15 mmol) and MeAl­(C_6_F_5_)_2_ (0.3 mL, 0.5 M in toluene, 0.15 mmol) in toluene (2 mL). After refluxing for 2 days the mixture was subjected to three cycles of freeze–pump–thaw degassing. CO_2_ was added (approximately 2 bar) and the mixture was stirred for 2 h causing a complete discoloration of the solution. After removal of the solvent the oily residue dissolved in hexane (2 mL) and stirred overnight. This caused precipitation of [Ph_2_P­(Ind-CO_2_)­Al­(C_6_F_5_)_2_]_2_, **4-CO**_**2**_ (72 mg, 0.10 mmol, 68%) as a colorless solid which was washed with hexane and dried under reduced pressure. Crystals suitable for X-ray diffraction analysis were grown by slow evaporation of a benzene/hexane mixture. **Note:** This compound undergoes ligand scrambling in solution (see SI for more information). ^1^H NMR (400 MHz, C_6_D_6_, 298 K): δ [ppm] = 7.30–6.50 (overlapping multiplets of **4-CO**_**2**_ and **4’-CO**_**2**_, 13 H, PPh_2_/H_Ar_), 3.81 (t, ^*3*^ *J*_*HH*_ = 7.4 Hz, 2 H, CH_2_), 2.39 (t, ^*3*^ *J*_*HH*_ = 7.4 Hz, 2 H, CH_2_). ^31^P­{^1^H} NMR (162 MHz, CD_2_Cl_2_, 298 K): δ [ppm] = −6.42 (q). ^19^F­{^1^H} NMR (376 MHz, CD_2_Cl_2_, 298 K): δ [ppm] = −123.18 (m, 4 F, *o*-C_6_ *F*_5_), 153.40 (t, *J* = 19.6 Hz, 1F, *o*-C_6_ *F*_5_), −161.5 (m, 4F, *p*-C_6_ *F*_5_). Anal. Calcd. For [C_33_H_18_AlF_10_NO_2_P]_2_: C, 55.95; H, 2.56; N, 1.98. Found: C, 55.21; H, 2.38; N, 1.99.

## Supplementary Material



## Notes

NMR spectra for all compounds (including supporting discussions), acceptor numbers for all alane compounds, additional experimental details, and crystallographic data (PDF)
