# Supplementary material for: Intramolecular Al/P Frustrated Lewis Pairs Based on an Indoline Backbone—Two Reaction Sites for Small Molecule Activation and Hydrodefluorination
Source: Inorg Chem. 2026 Jun 17;65(26):14926–35. doi: 10.1021/acs.inorgchem.6c01812 (PMC13343510; doi:10.1021/acs.inorgchem.6c01812)
Supplement: Supplementary file 1 [file ic6c01812_si_001.pdf]

Supporting Information

Intramolecular Al/P Frustrated Lewis-pairs based on an Indoline backbone – Two Reaction Sites for Small molecule Activation and Hydrodefluorination

Sanjay Biswas, Siad Wolff, Beatrice Cula, Christian Limberg*

Institut für Chemie, Humboldt-Universität zu Berlin, Brook-Taylor-Straße 2, 12489 Berlin.

Email: christian.limberg@chemie.hu-berlin.de

Table of content

1. General Remarks 2

2. Experimental Section 3

2.1. Synthesis of 7-diphenylphosphino *N*-Boc-indoline....................3

2.2. Synthesis of 7-diphenylphosphino indoline, $Ph_2P(Ind)H$ 6

2.3. Synthesis of $Ph_2P(Ind)K$....................9

2.4. Synthesis of $(Ind)AlMe_2$ 12

2.5. Synthesis of $[Ph_2P(Ind)AlCl_2]_2$, 1 15

Isolation of $[Ph_2P(Ind)]_2AlCl(THF)$, 1'-THF **21**

2.6. Synthesis of $[Ph_2P(Ind)AlI_2]_2$, 2....................23

2.7. Synthesis of $Ph_2P(Ind)AlMe_2$, 3....................29

2.8. Synthesis of $Ph_2P(Ind)Al(C_6F_5)_2$, 4 32

2.9. Synthesis of $Ph_2P(Ind)Al(Me)_2(Ph_2CN_2)$, 3-CNN 36

2.10 Synthesis of $Ph_2P(Ind)Al(C_6F_5)_2(Ph_2CN_2)$, 4-CNN 39

2.11. Synthesis of $Ph_2P(Ind\text{-}CO_2)Al(Me)_2$, 3-$CO_2$....................42

2.12. Synthesis of $Ph_2P(Ind)Al(C_6F_5)_2CO_2$, 4-$CO_2$....................45

3. Fluorescence of 3 48

4. Acceptor Number by Gutmann-Beckett....................50

5. Dehydrodefluorination (DHF) reaction studies 53

5.1. DHF mediated by 3 53

5.2. DHF mediated by 4 56

5.3. DHF mediated by $(Ind)AlMe_2$....................58

5.4. DHF mediated by $AlMe_3$ 59

6. Crystallographic Data 62

7. References....................71

1. General Remarks

All experiments were carried out in a dry argon or nitrogen atmosphere using an MBraun glovebox, GS Glovebox Systemtechnik glovebox and/or standard Schlenk techniques. Solvents were purified employing an MBraun Solvent Purification System SPS. Elemental analyses were performed with a HEKA Euro 3000EA elemental analyser. NMR Spectra were recorded on Bruker NMR spectrometers (Avance II 300 MHz, Avance NEO 300 MHz, Avance 400 MHz, Avance III 500 MHz, Avance 600 MHz). Chemical shifts are referenced to the signal of residual protonated solvent. ATR-infrared (IR) spectra were recorded with a Bruker alpha FTIR spectrometer and GC-MS were recorded using a *Varian* MS4000 (136).

All materials were obtained from commercial vendors as ACS reagent-grade or better and used as received, if not stated otherwise. N-Boc-indoline,[1] $Al(C_6F_5)_3$•0.5 Tol[2], $(C_6F_5)_2AlCl$[3], diphenyldiazomethane[4] and benzyl potassium[5] were prepared as described in the literature. 7-diphenylphosphino *N*-Boc indoline[6] was prepared by modified synthetic procedure described in the literature. A 0.5 M stock solution of $MeAl(C_6F_5)_2$ was prepared by stirring a suspension of trimethylaluminum (2 M in toluene, 221 µL, 442 µmol, 1.00 eq) and $Al(C_6F_5)_3$•0.5 Tol (507 mg, 884 µmol, 2.00 eq) in 2.2 mL toluene for 2 hours.

Caution! The aluminium compounds bearing pentafluorophenyl substituents prepared in this manuscript are potentially shock and thermally sensitive due to the potential formation of aluminium fluorides and benzyne intermediates. Appropriate care should be taken.

2. Experimental Section

2.1. Synthesis of 7-diphenylphosphino *N*-Boc-indoline

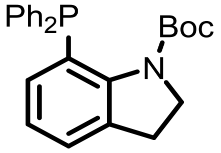

N-Boc-indoline (2.00 g, 9.12 mmol, 1.00 eq) was dissolved in 10 mL dry Et_2O and cooled to –78 °C. *t*-Butyllithium (1.7 M in pentane, 6.43 mL, 10.94 mmol, 1.20 eq) was added dropwise and subsequently stirred at –78°C for 1 hour. A solution of phenoxydiphenylphosphine (3.05 g, 10.94 mmol, 1.20 eq) in 10 mL of dry Et_2O was added and stirred for another hour at –78°C. The cooling bath was removed and the yellowish reaction mixture was stirred at room temperature for 24 hours. Excessive *t*-BuLi was quenched with 20 mL of saturated NH_4Cl solution and the mixture was extracted with DCM (3 × 20 mL). The combined organic phases were dried over Na_2SO_4 and concentrated. The crude product was recrystallized from hexane (~30 mL). This yielded the product as off-white solid (1.58 g, 3.92 mmol, 42%).

Crystals suitable for X-ray diffraction analysis were grown via slow evaporation of a dichloromethane solution.

^{1}H NMR (300 MHz, $CDCl_3$, 298 K): δ [ppm] = 7.42-7.37 (m, 4H, *o*-PPh_2), 7.30-7.27 (m, 6H, *m*-PPh_2 and *p*-PPh_2), 7.12 (dd, $^3J_{HH}$ = 7.3 Hz, $^4J_{HH}$ = 1.3 Hz, H_a), 6.89 (t, $^3J_{HH}$ = 7.5 Hz, 1H, H_b), 6.77 (ddd, $^3J_{HH}$ = 7.9 Hz, $^3J_{HH}$ = 5.1 Hz, $^4J_{HP}$ = 1.3 Hz 1H, H_c), 3.94 (t, $^3J_{HH}$ = 7.9 Hz, 2H, H_e), 3.02 (t, $^3J_{HH}$ = 7.9 Hz, 2H, H_d), 1.33 (s, 9H, ^{t}Bu).

^{13}C{^{1}H} NMR (75 MHz, $CDCl_3$, 298 K): δ [ppm] = 152.81 (d, $^4J_{CP}$ = 3 Hz, C=O), 145.2 (d, $^2J_{CP}$ = 15 Hz, ind-*C*-NCH2), 138.59 (d, $^1J_{CP}$ = 13.5 Hz, PhP-*C*-C_5H_5), 134.22 (d, $^2J_{CP}$ = 21.75 Hz, *C*-H_o), 133.92 (d, $^2J_{CP}$ = 3 Hz, *C*-H_a), 133 (d, $^3J_{CP}$ = 4.5 Hz, ind-*C*-CH_2CH_2), 128.45 (s, *C*- H_m/ H_p), 128.17 (s, H_m/ H_p), 126.85 (s, *C*-H_c), 124.19 (s, *C*-H_b), 123.82 (s, *C*-PPh_2), 80.60 (s, $C(CH_3)_3$), 49.76 (s, *C*-H_e), 29.29 (s, *C*-H_d), 28.28 (s, CH_3).

^{31}P{^{1}H} NMR (121 MHz, $CDCl_3$, 298 K): δ [ppm] = –8.74 (s).

Elemental analysis: $C_{25}H_{26}NO_2P$ Calculated: C 74.42% H 6.50% N 3.47%

Found: C 74.47% H 6.41% N 3.47%

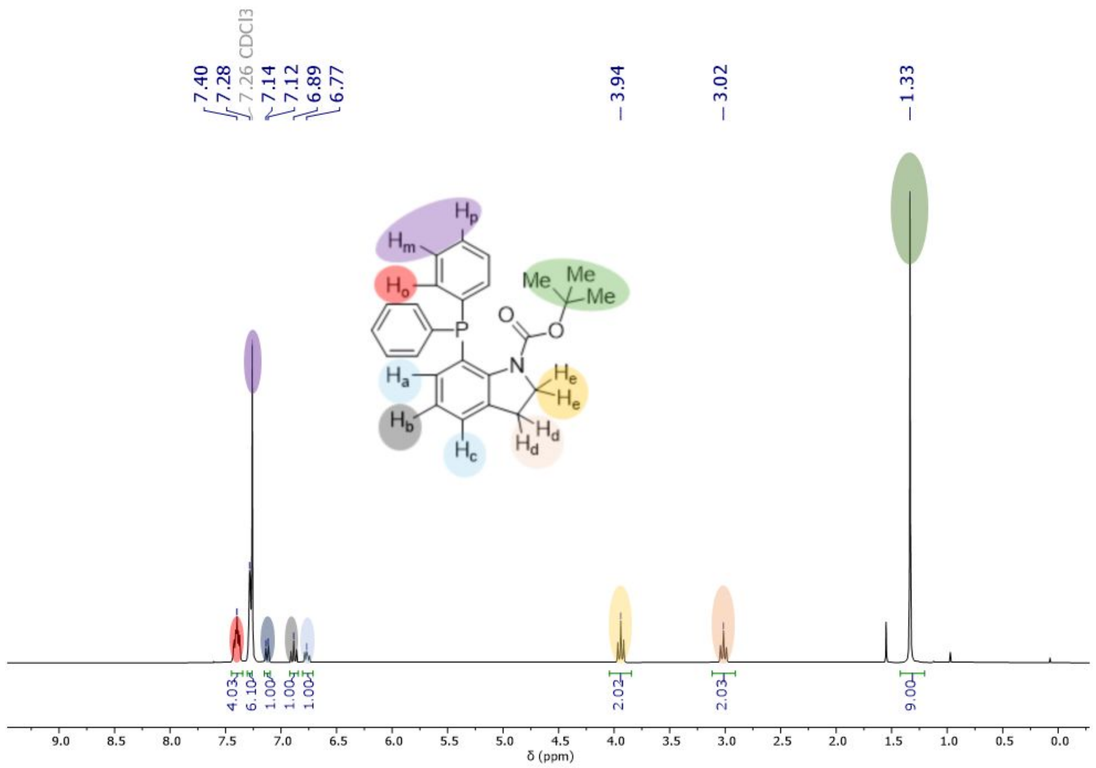

Figure S1. ^{1}H NMR spectrum of **7-diphenylphosphino N-Boc-indoline** (300 MHz, $CDCl_3$, 298 K).

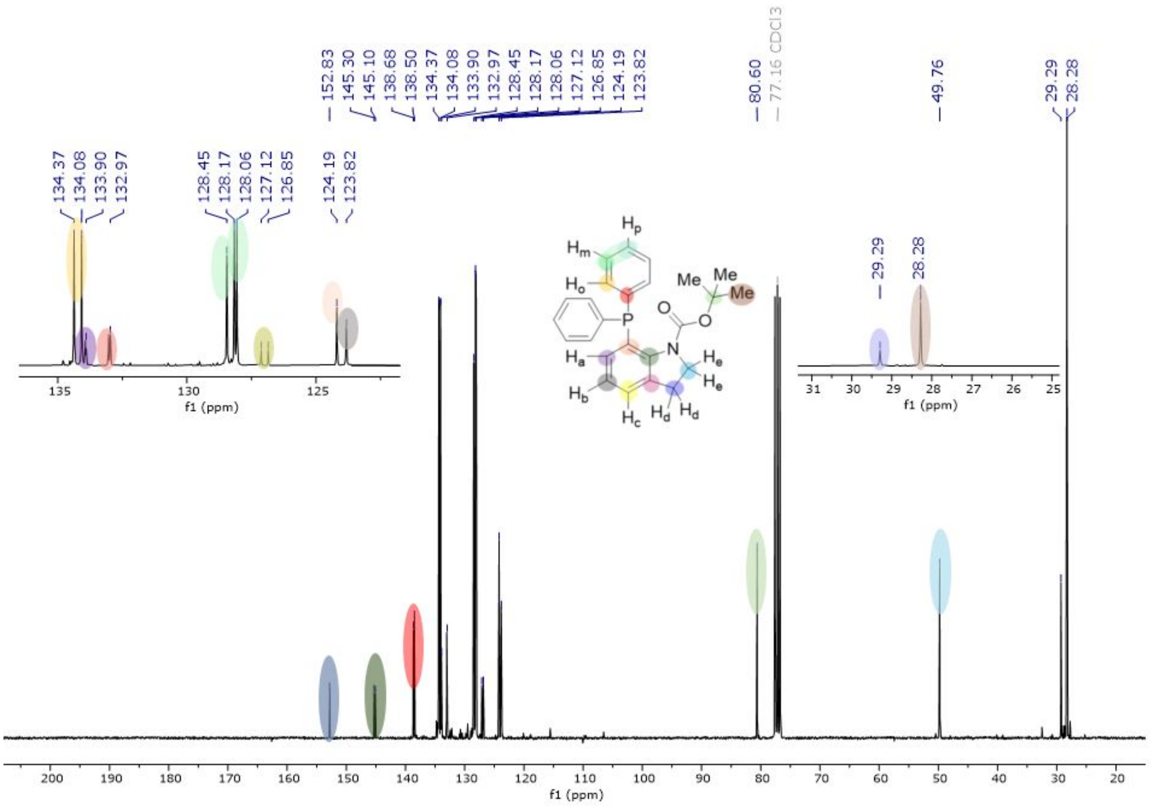

Figure S2. $^{13}C\{^{1}H\}$ NMR spectrum of **7-diphenylphosphino N-Boc-indoline** (75 MHz, $CDCl_3$, 298 K).

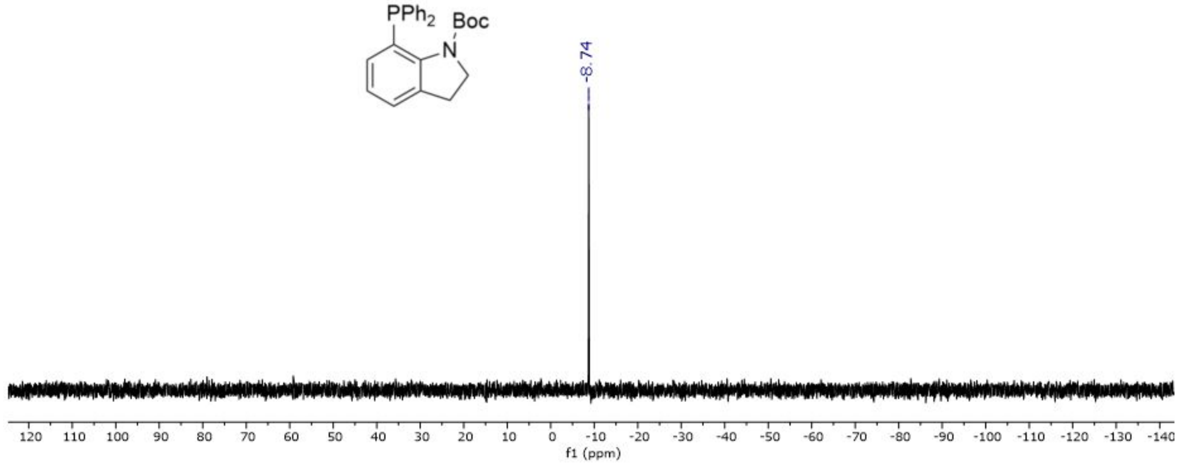

Figure S3. $^{31}P\{^1H\}$ NMR spectrum of **7-diphenylphosphino *N*-Boc-indoline** (121 MHz, $CDCl_3$, 298 K).

2.2. Synthesis of 7-diphenylphosphino indoline, $Ph_2P(Ind)H$

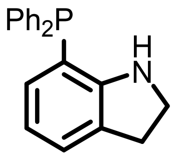

7-diphenylphosphino *N*-Boc-indoline (1.00 g, 2.48 mmol) was dissolved in 10 mL of DCM. After addition of CF_3COOH (6 mL) the reaction mixture was stirred at room temperature for 24 hours. Then saturated aqueous $NaHCO_3$ solution was added until no more gas evolution was observed. Subsequently, the mixture was extracted with CH_2Cl_2 (3 × 20 mL). The organic phase was dried over Na_2SO_4 and the solvent was removed under vacuum. The crude sticky product was recrystallized from hexane to give the pure desired compound (547 mg, 1.8 mmol, 72%) as white powder.

Crystals suitable for X-ray diffraction analysis were grown via slow evaporation of a dichloromethane solution.

^{1}H NMR (300 MHz, C_6D_6, 298 K): δ [ppm] = 7.52-7.45 (m, 4H, *o*-PPh_2), 7.10-7.01 (m, 6H, *m*-PPh_2 and *p*-PPh_2), 6.98-6.94 (m, 2H, H_a, H_c), 6.62 (t, $^3J_{HH}$ = 7.4 Hz, 1H, H_b), 3.94 (s, 1H, NH), 2.90 (t, $^3J_{HH}$ = 8.4 Hz, 2H, H_e), 2.60 (t, $^3J_{HH}$ = 8.4 Hz, 2H, H_d).

^{13}C{^{1}H} NMR (75 MHz, C_6D_6, 298 K): δ [ppm] 156.08 (d, $^2J_{CP}$ = 18.4 Hz, ind-*C*-NCH2), 136.95 (d, $^1J_{CP}$ = 10.2 Hz, PhP-*C*-C_5H_5), 134.01 (d, $^2J_{CP}$ = 19.1 Hz, *C*-H_o), 132.47 (d, $^2J_{CP}$ = 6.6 Hz, *C*-H_a), 128.90 (s, ind-*C*-CH_2CH_2), 128.81 (s, *C*-H_m/ H_p), 128.79 (s, *C*- H_m/ H_p), 125.49 (s, *C*-H_c), 118.81 (d, $^3J_{CP}$ = 3.4 Hz, *C*-H_b), 114.37 (d, $^1J_{CP}$ = 9.6 Hz, *C*-PPh_2), 46.96 (s, *C*-H_e), 29.72 (d, $^4J_{CP}$ = 2.21 Hz, *C*-H_d).

^{31}P{^{1}H} NMR (121 MHz, C_6D_6, 298 K): δ [ppm] = –17.75 (s).

Elemental Analysis: $C_{20}H_{18}NP$ Calculated: C 79.19% H 5.98% N 4.62%

Found: C 79.01% H 5.83% N 4.61%

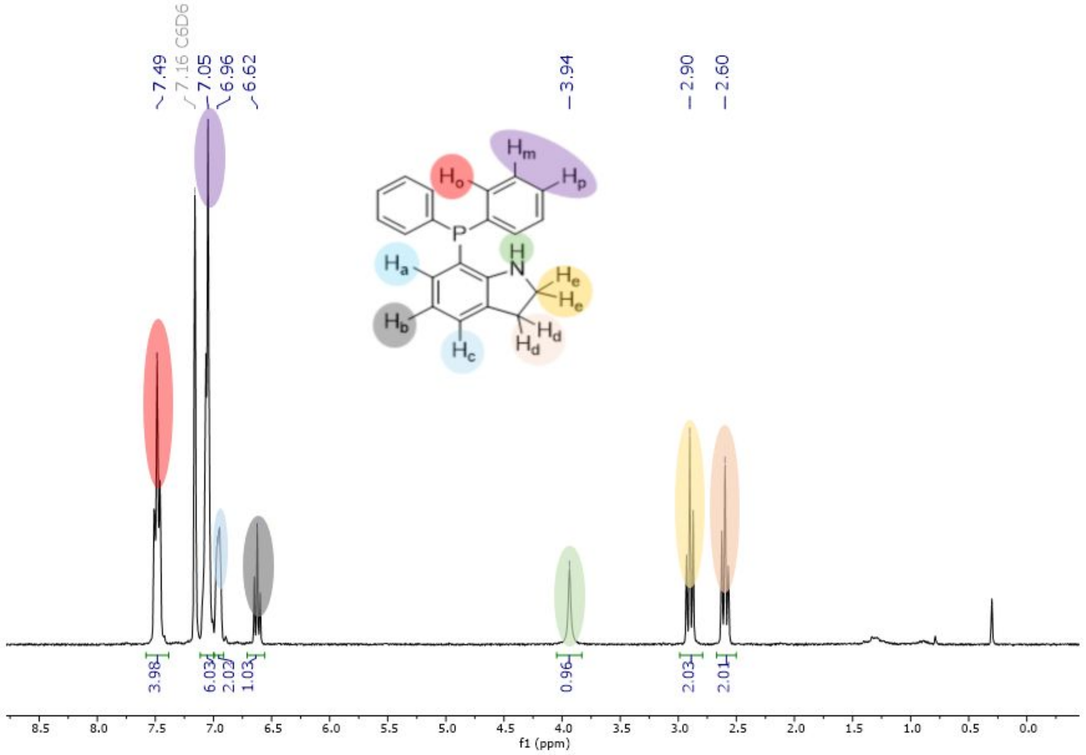

Figure S4. ^{1}H NMR spectrum of **$Ph_2P(Ind)H$** (300 MHz, C_6D_6, 298 K).

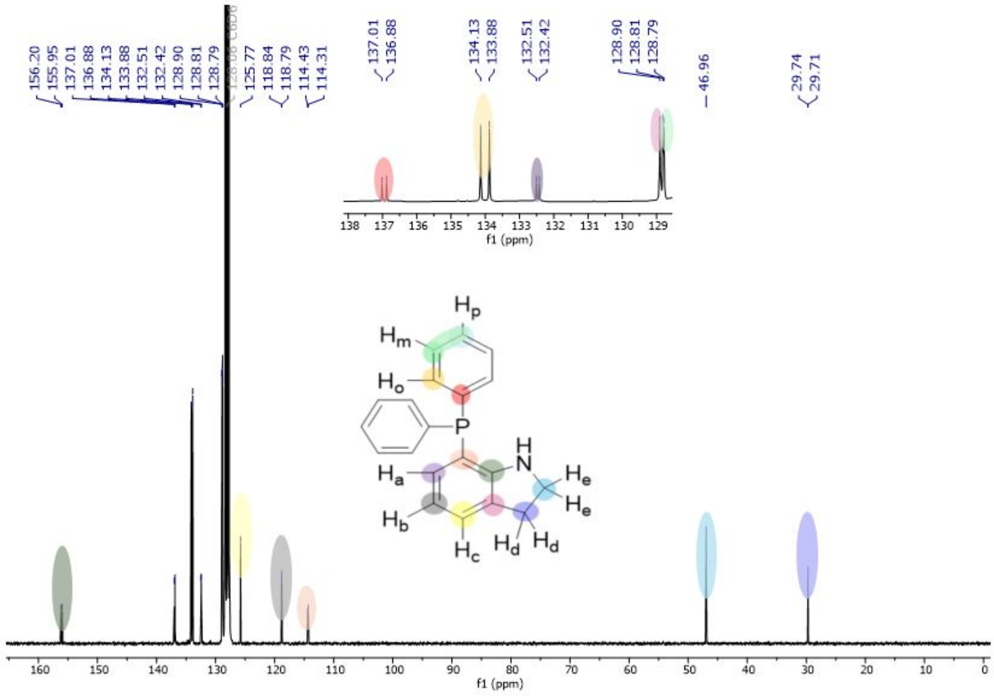

Figure S5. $^{13}C\{^{1}H\}$ NMR spectrum of **$Ph_2P(Ind)H$** (75 MHz, C_6D_6, 298 K).

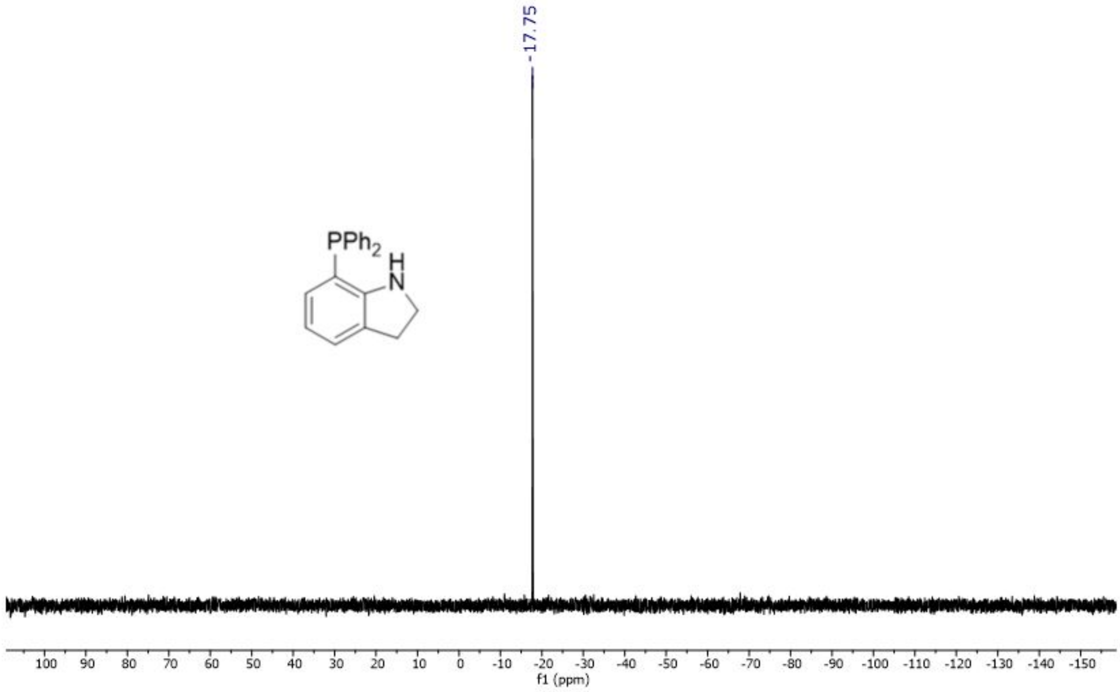

Figure S6. $^{31}P\{^{1}H\}$ NMR spectrum of **$Ph_2P(Ind)H$** (121 MHz, C_6D_6, 298 K).

2.3. Synthesis of $Ph_2P(Ind)K$

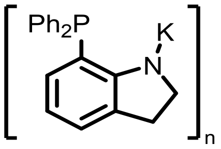

Benzyl potassium (944 mg, 7.25 mmol, 1.10 eq) was added at room temperature to a solution of Ph_2P(Ind)H (2.00 g, 6.59 mmol, 1.00 eq) in toluene (15 mL). The yellow solution was stirred for 2 h at room temperature. The yellow precipitate was washed with hexane and dried under high *vacuo* for 4 h to almost quantitatively yield the bright yellow compound **Ph_2P(Ind)K** (1.8 g, 5.27 mmol, 80%).

Crystals suitable for X-ray diffraction analysis were grown via slow evaporation of a toluene solution.

^{1}H NMR (300 MHz, THF-d_8, 298 K): δ [ppm] = 7.35-7.30 (m, 4H, *o*-PPh_2), 7.27-7.20 (m, 6H, *m*-PPh_2 and *p*-PPh_2), 6.46 (d, $^{3}J_{HH}$ = 7.4 Hz, 1H, H_a), 5.88 (t, $^{3}J_{HH}$ = 7.1 Hz, 1H, H_c), 5.37 (t, $^{3}J_{HH}$ = 7.1 Hz, 1H, H_b), 3.65 (t, $^{3}J_{HH}$ = 8.8 Hz, 2H, H_e), 2.76 (t, $^{3}J_{HH}$ = 8.8 Hz, 2H, H_d).

^{13}C{^{1}H} NMR (75 MHz, THF-d_8, 298 K): δ [ppm] 140.84 (d, $^{2}J_{CP}$ = 9.75 Hz, ind-*C*-NCH_2), 134.96 (d, $^{1}J_{CP}$ = 18 Hz, PhP-*C*-C_5H_5), 132.49 (s, *C*-H_o), 131.73 (d, $^{2}J_{CP}$ = 5.25 Hz, *C*-H_a), 129.84 (s, ind-*C*-CH_2CH_2), 128.87 (s, *C*- H_m/ H_p), 128.38 (s, *C*- H_m/ H_p), 126.20 (s, *C*-H_c), 123.26 (s, *C*-H_b), 105.58 (s, *C*-PPh_2), 55.69 (s, *C*-H_e), 32.75 (s, *C*-H_d).

^{31}P{^{1}H} NMR (121 MHz, THF-d_8, 298 K): δ [ppm] = –17.29 (s).

Elemental Analysis: $[C_{20}H_{17}KNP]_n$ Calculated: C 70.36% H 5.02% N 4.10%

Found: C 68.39% H 4.94% N 3.51%

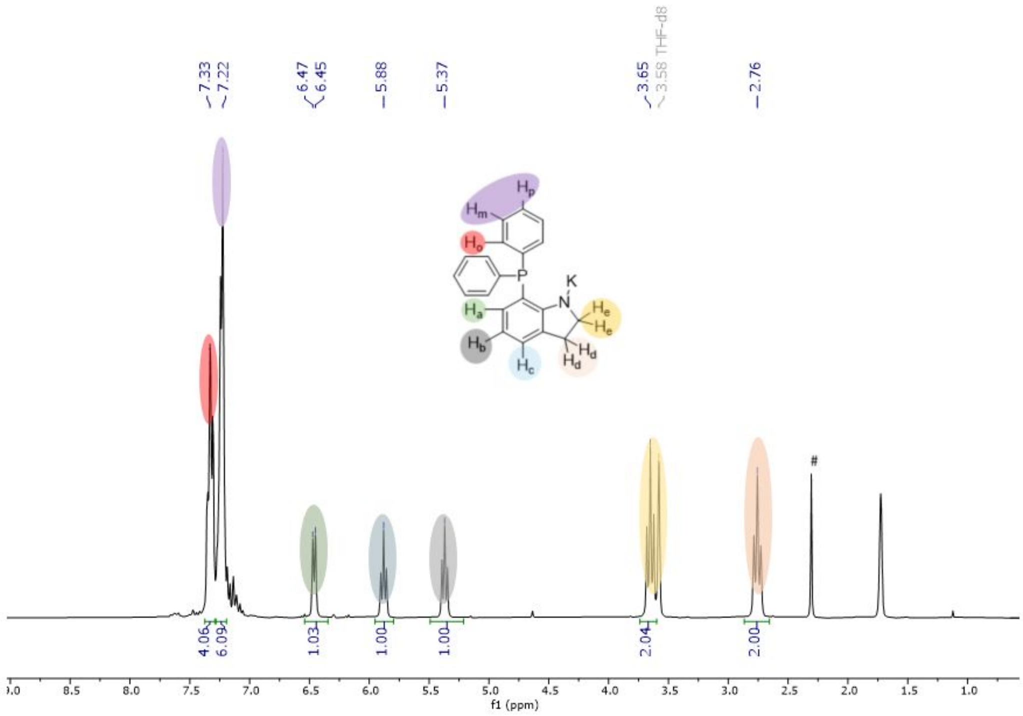

Figure S7. ^{1}H NMR spectrum of **$Ph_2P(Ind)K$** (300 MHz, THF-d_8, 298 K, # = co-crystallized toluene solvate).

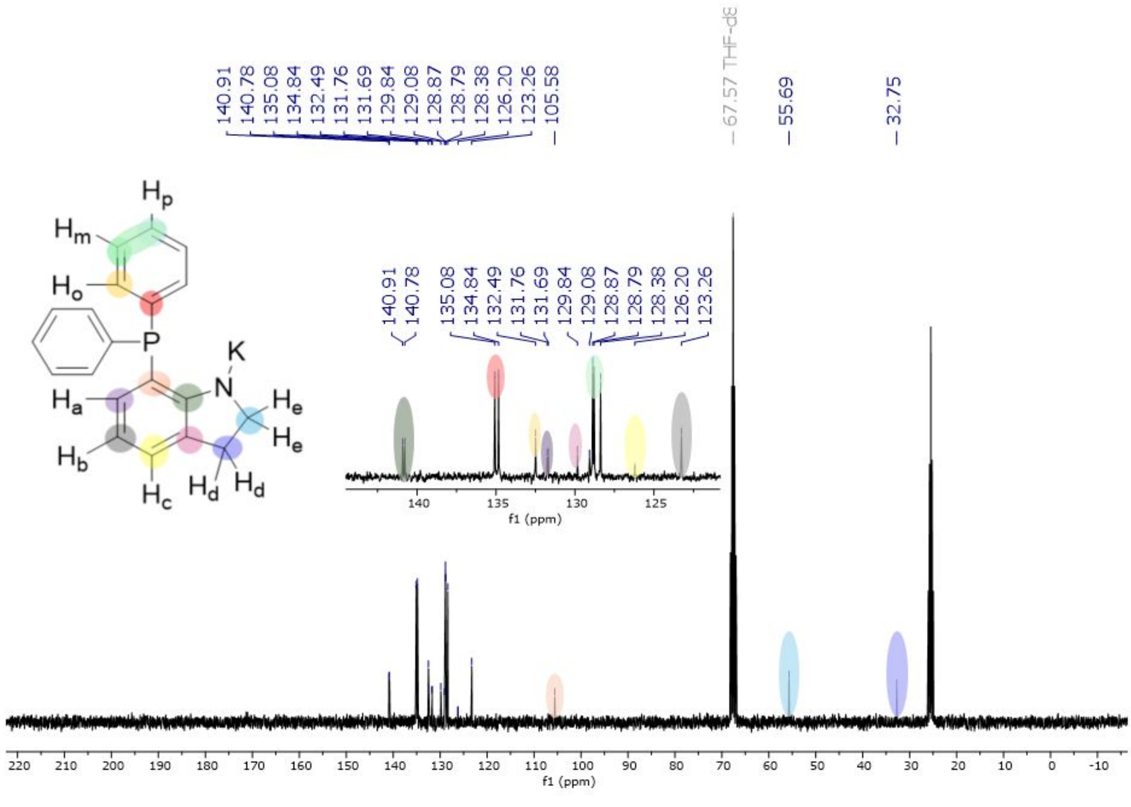

Figure S8. $^{13}C\{^{1}H\}$ NMR spectrum of **$Ph_2P(Ind)K$** (75 MHz, THF-d_8, 298 K).

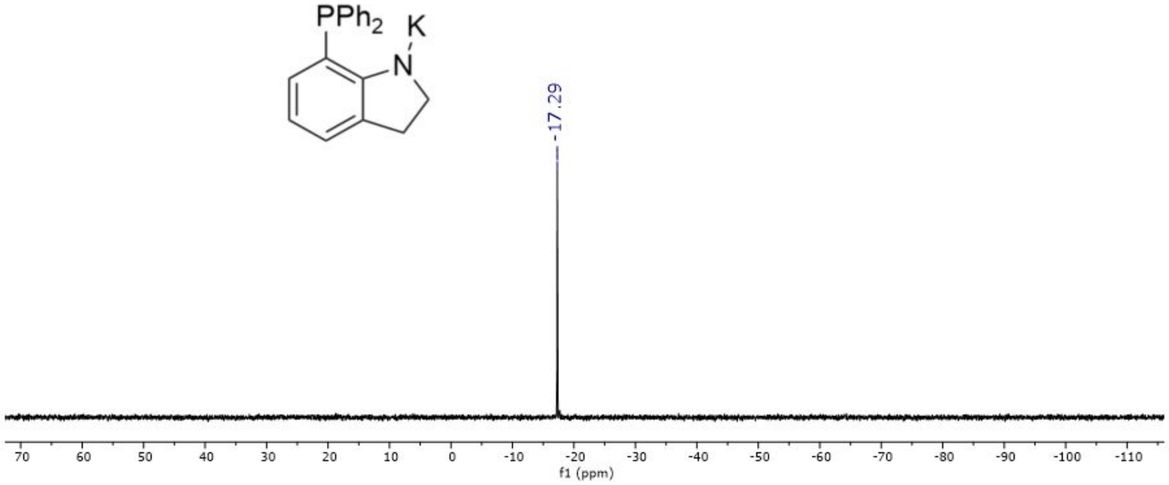

Figure S9. $^{31}P\{^1H\}$ NMR spectrum of **Ph₂P(Ind)K** (121 MHz, THF-d_8, 298 K).

2.4. Synthesis of (Ind)AlMe₂

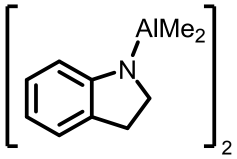

Indoline (1.00 g, 8.39 mmol, 1.00 eq) was dissolved in 10 mL of dry toluene and $AlMe_3$ (4.19 mL, 2.00 M in toluene, 8.39 mmol, 1.00 eq) was added slowly. After stirring for 2 h at room temperature the solvent was removed *in vacuo* and the residue was washed with 5 mL of dry hexane. The obtained colorless solid material was dried in high *vacuo* for 2 h to yield **(Ind)AlMe$_2$** (980 mg, 5.59 mmol, 66%).

Crystals suitable for X-ray diffraction analysis were obtained by recrystallization from a boiling hexane solution.

Note: XRD measurements revealed that **(Ind)AlMe$_2$** adopts a dimeric structure **[(Ind)AlMe$_2$]$_2$** in the solid state with a *trans* orientation of the bridging indoline ligands (see Scheme S1), suggesting that all four methyl groups are chemically equivalent. However, NMR spectroscopic analysis showed that upon dissolution in non-coordinating solvents, two species are present, one of which exhibits two distinct signals for the methyl groups. This observation was reproducible even after several recrystallizations of **(Ind)AlMe$_2$.** DOSY NMR measurements demonstrated that both species possess similar diffusion coefficients, indicating that they both exist as dimers in solution. Moreover, ROESY experiments revealed exchange between the aromatic signals of the indoline ligands. Based on these findings, it was concluded that **[(Ind)AlMe$_2$]$_2$** undergoes a *cis–trans* isomerization upon dissolution. For determination of its acceptor number, **[(Ind)AlMe$_2$]$_2$** was treated with Et_3PO (see Section 3). Coordination of the donor molecule led to cleavage of the dimeric structure, resulting in the formation of a single species in solution (see Figure S50).

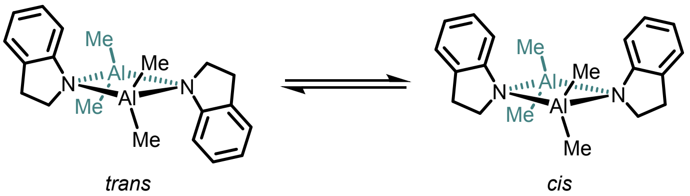

Scheme S1: Proposed *cis–trans* isomerization of **(Ind)AlMe$_2$**.

<u>*trans* isomer</u>

^{1}H NMR (300 MHz, C_6D_6, 298 K): δ [ppm] = 7.24 (d, 1 H, H_{Ar}), 7.02-6.99 (m, 2 H, H_{Ar}), 6.90-6.84 (m, overlapping with signal of *cis* isomer, 1 H, H_{Ar}), 3.41 (t, $^3J_{HH}$ = 7.7 Hz, 2 H, NCH_2CH$_2$), 2.54 (t, overlapping with signal of *cis* isomer, 2 H, NCH$_2$$CH_2$), –0.51 (s, 6 H, AlMe$_2$).

<u>*cis* isomer</u>

^{1}H NMR (300 MHz, C_6D_6, 298 K): δ [ppm] = 7.44 (d, 1 H, H_{Ar}), 7.08-7.04 (m, 2 H, H_{Ar}), 6.90-6.84 (m, overlapping with signal of *cis* isomer, 1H, H_{Ar}), 3.32 (t, $^3J_{HH}$ = 7.7 Hz, 2 H, NCH_2CH$_2$), 2.54 (t, overlapping with signal of *cis* isomer, 2 H, NCH$_2$$CH_2$), –0.37 (s, 3 H, AlMe$_2$), –0.57 (s, 3 H, AlMe$_2$).

Due to presence of two species in solution recording of ^{13}C NMR data was not pursued.

Elemental Analysis: $[C_{10}H_{14}AlN]_2$ Calculated: C 68.55% H 8.05% N 7.99%

Found: C 67.27% H 7.77% N 7.88%

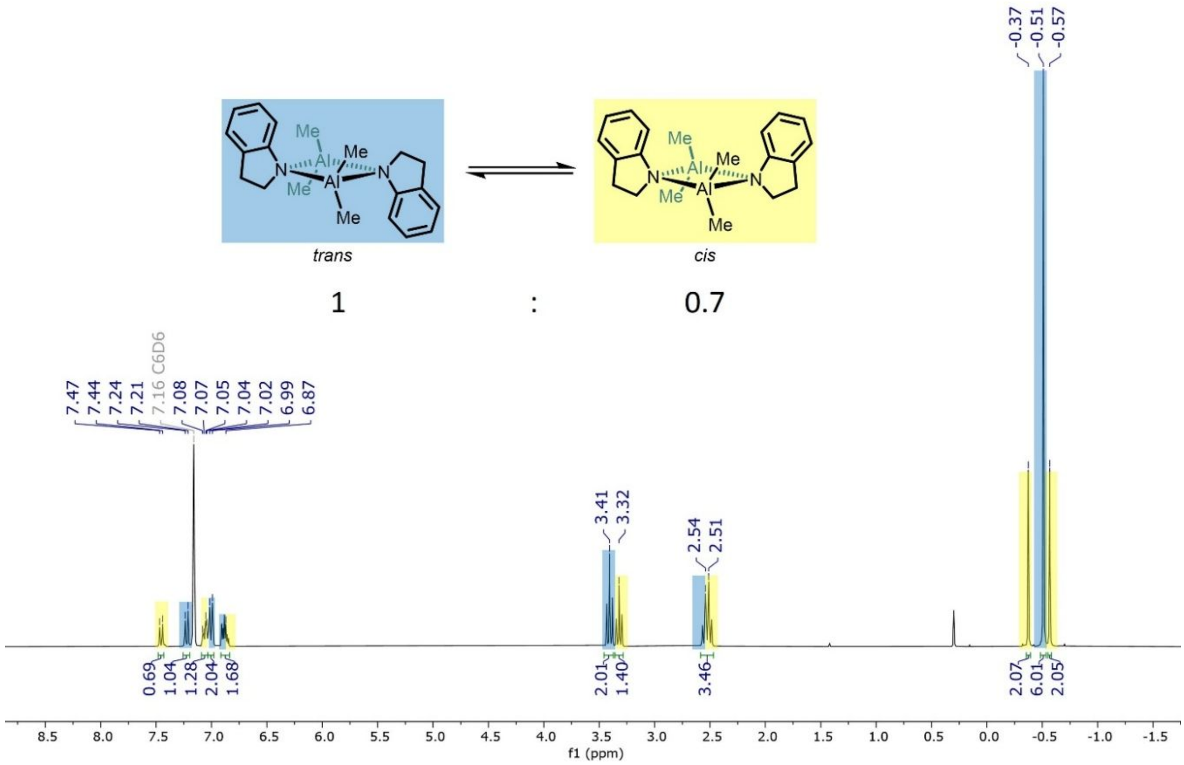

Figure S10. ^{1}H NMR spectrum of **[(Ind)AlMe$_2$]$_2$** (300 MHz, C_6D_6, 298 K).

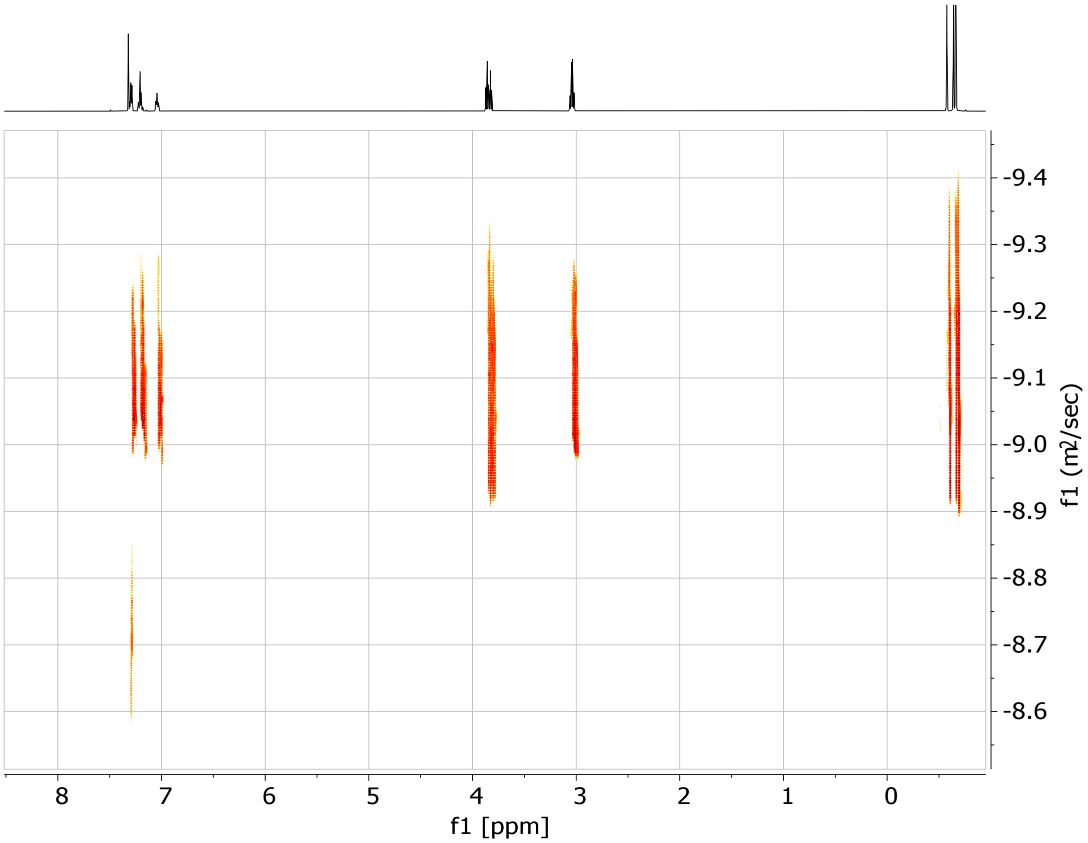

Figure S11. DOSY NMR spectrum of **[(Ind)AlMe$_2$]$_2$** (600 MHz, $CDCl_3$, 298 K).

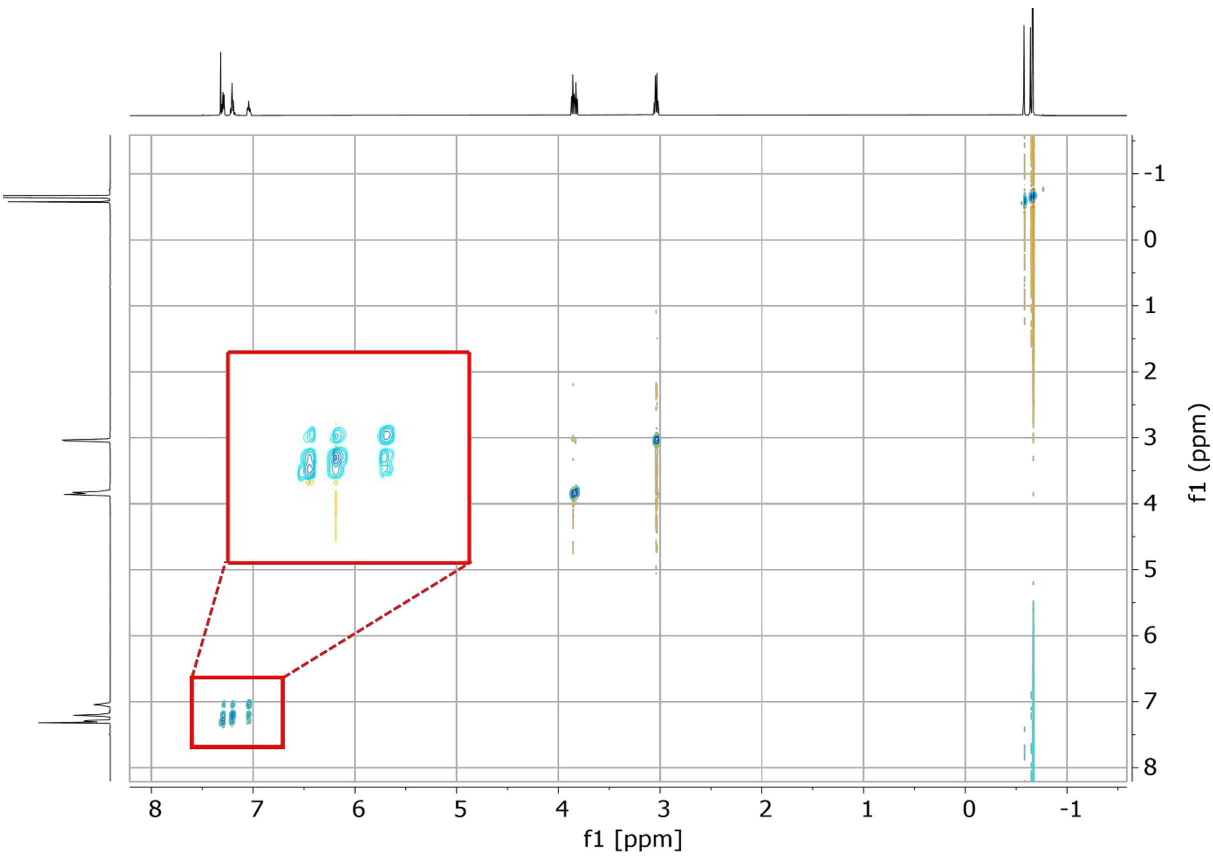

Figure S12. ^{1}H-^{1}H-ROESY NMR spectrum of **[(Ind)AlMe$_2$]$_2$** (600 MHz, $CDCl_3$, 298 K).

2.5. Synthesis of $[Ph_2P(Ind)AlCl_2]_2$, 1

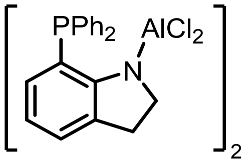

$EtAlCl_2$ (1.00 mL, 1.00 mmol, 1 M in hexane, 1.00 eq) was added at room temperature to a suspension of $Ph_2P(Ind)H$ (303 mg, 1.00 mmol, 1.00 eq), in DCM (3 mL). After refluxing the bright yellow solution for 1 h the volume was reduced to half. Storing at –30 °C afforded $[Ph_2P(Ind)AlCl_2]_2$, **1**, as a colorless crystalline material. Further concentration and storage at –30°C afforded another batch of crystals (combined yield: 340 mg, 0.85 mmol, 85%).

Single Crystals suitable for X-ray diffraction analysis were grown via slow evaporation of the volatiles of a DCM/toluene mixture.

NOTE: After crystallization, compound **1** exhibited only limited solubility in aromatic solvents, requiring characterization in chlorinated solvents. Although **1** was isolated as a colorless crystalline solid, it displayed a fluorescent yellow color upon dissolution.

Elemental analysis of the colorless crystalline material confirmed that the sample features the expected molar ratio of $[Ph_2P(Ind)AlCl_2]_2$, confirming purity of the sample. However, analysis by ^{1}H and ^{31}P NMR spectroscopy revealed the presence of two species in solution. One species showed an asymmetric chemical environment for the aliphatic CH_2 groups of the indoline ligand (see Figure S13, blue labels). This species was assigned to the dimeric structure of **1**, as identified by XRD analysis (see main text, Figure 2). The second species exhibited a symmetric environment and was denoted as **1'** (see Figure S13, yellow labels). DOSY NMR analysis indicated that **1** possesses a slightly larger hydrodynamic radius compared to **1'**. For comparison, a DOSY NMR spectrum of the monomeric compound $Ph_2P(Ind)AlMe_2]_2$, **3**, was recorded, which features a larger diffusion coefficient/smaller hydrodynamic radius compared to **1** and **1'** (see Figure S31).

To gain further insight into the nature of **1'**, variable-temperature NMR studies were performed (see Figure S16). Upon increasing the temperature, the signals corresponding to **1** gradually decreased in intensity, while those assigned to **1'** increased. The reverse behaviour was observed upon cooling. These observations demonstrate that **1** and **1'** participate in a temperature-dependent equilibrium that is fully reversible over multiple heating/cooling cycles. It is therefore proposed that **1** undergoes a monomer–dimer equilibrium in non-coordinating solvents, with **1'** corresponding to the monomeric complex $Ph_2P(Ind)AlCl_2$. This interpretation is also consistent with the DOSY NMR results.

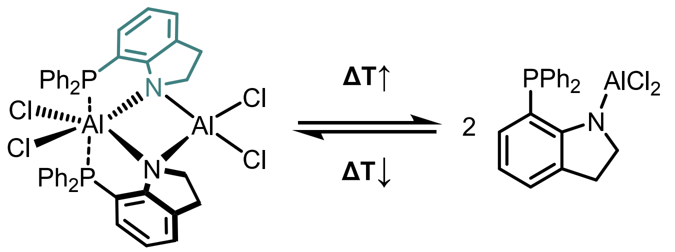

Scheme S2: Temperature-dependent monomer-dimer equilibrium of **1**.

For determination of its acceptor number, **1** was treated with Et_3PO (see Section 3). Coordination of the donor molecule led to cleavage of the dimeric structure, resulting in the formation of a single species in solution (see Figure S51). In addition, the ^{31}P NMR signals became sharp, indicating disruption of the Al–P interaction. Surprisingly, treatment of **1** was with THF instead of Et_3PO did not lead to complete conversion into a single species; two species were observed in solution. Concentration of the NMR sample led to crystallization of $[Ph_2P(Ind)]_2AlCl(THF)$, which was assigned as **1*-THF**. These observations suggest that compound **1** is prone to ligand scrambling in solution (see Scheme S3). Nevertheless, the crystalline material obtained from non-coordinating solvents corresponds to the targeted product and displays the expected molecular stoichiometry $Ph_2P(Ind)AlCl_2$ (see results of elemental analysis).

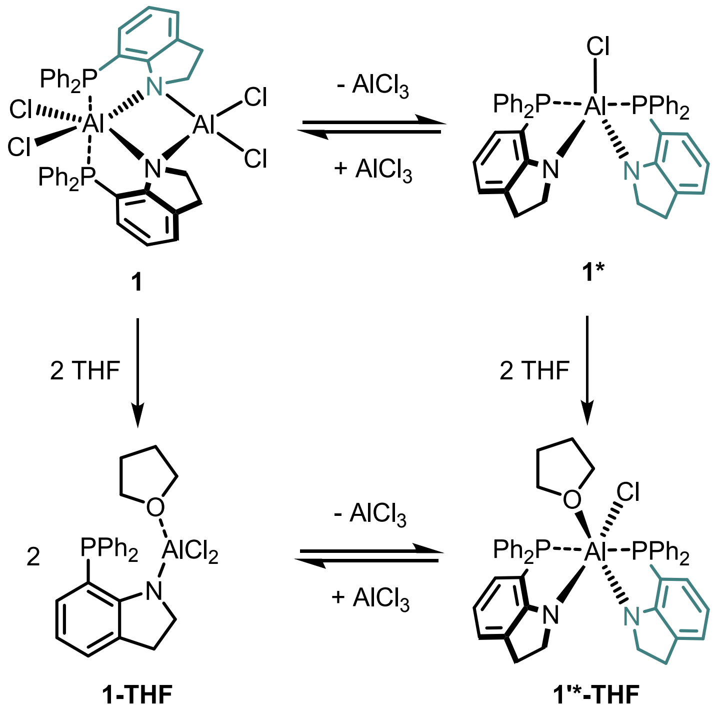

Scheme S3: Proposed ligand scrambling of **1**.

Elemental Analysis: $[C_{40}H_{34}Al_2Cl_4N_2P_2]_2 \bullet (C_7H_8)_2 \bullet CH_2Cl_2$

Calculated: C 61.02% H 4.64% N 3.00%

Found: C 60.75% H 4.64% N 2.83%

For elemental analysis, the same crystalline material used for the X-ray diffraction measurement was submitted. The crystal structure revealed co-crystallization of two toluene molecules and one dichloromethane molecule per two formula units of compound **1**. This solvent stoichiometry was consistent with the results obtained from elemental analysis.

<u>Spectroscopic data for $[Ph_2P(Ind)AlCl_2]_2$, **1**</u>

^{1}H NMR (600 MHz, $CDCl_3$, 298 K): δ [ppm] = 7.93 (t, 4 H, H_{PPh2}), 7.81 (t, 4 H, H_{PPh2}), 7.50 - 7.10 (overlapping multiplets of **1** and **1**′, 18 H, $H_{Ar/PPh2}$), 3.63 (m, 2 H, N$CH_2$$CH_2$), 2.97 (m, 2 H, N$CH_2$$CH_2$), 2.81 (m, 2 H, N$CH_2$$CH_2$), 2.66 (m, 2 H, N$CH_2$$CH_2$).

^{31}P{^{1}H} NMR (243 MHz, $CDCl_3$, 298 K): δ [ppm] = –27.00 (bs).

<u>Spectroscopic data for $[Ph_2P(Ind)]_2AlCl$, **1’**</u>

^{1}H NMR (600 MHz, $CDCl_3$, 298 K): δ [ppm] = 7.61 (t, 4 H, H_{PPh2}), 7.50 - 7.10 (overlapping multiplets of **1** and **1**′, 20 H, $H_{Ar/PPh2}$), 6.89 (t, 2 H, H_{Ar}), 6.46 (t, 2 H, H_{Ar}), 3.81 (m, 4 H, N$CH_2$$CH_2$), 3.18 (m, 4 H, N$CH_2$$CH_2$).

^{31}P{^{1}H} NMR (243 MHz, $CDCl_3$, 298 K): δ [ppm] = –30.00 (bs).

Due to presence of two species in solution recording ^{13}C NMR data was not pursued. ^{31}P NMR signals have been assigned by recording a ^{1}H-^{31}P HMBC NMR spectrum.

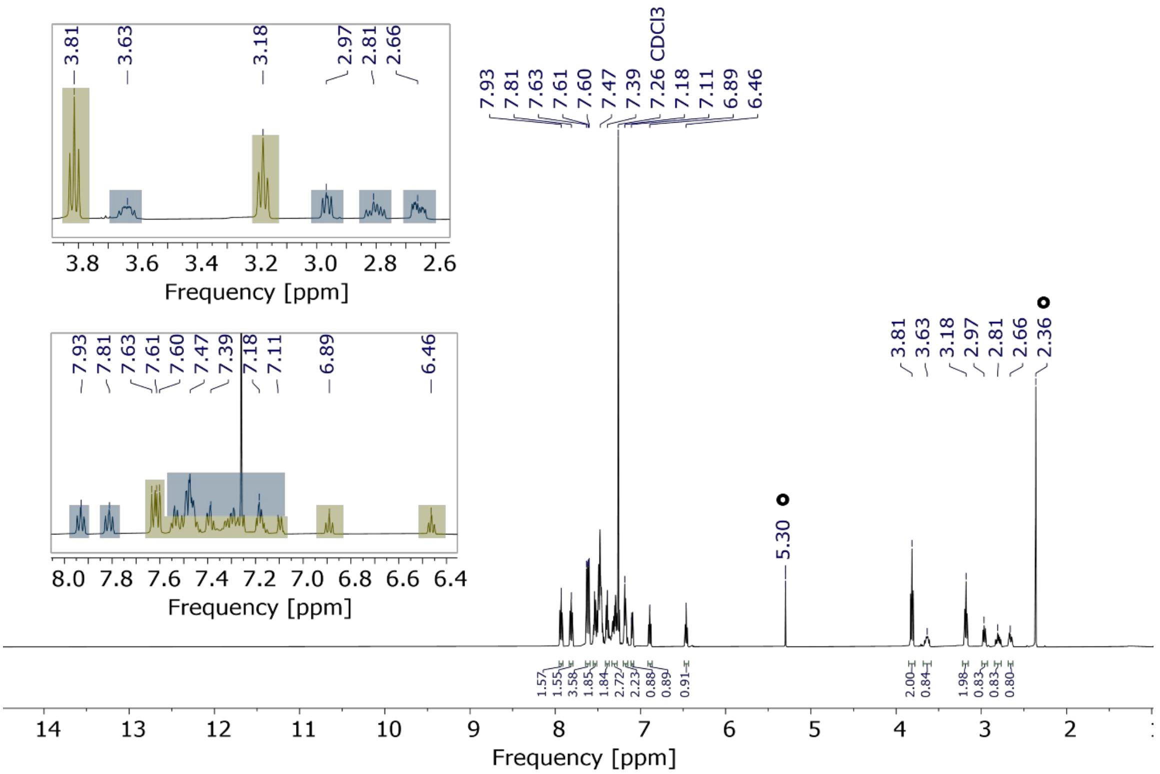

Figure S13. ^{1}H NMR spectrum of **1** (600 MHz, $CDCl_3$, 298 K, blue labels = **1**, yellow label = **1’**, ° = co-crystallized DCM and toluene).

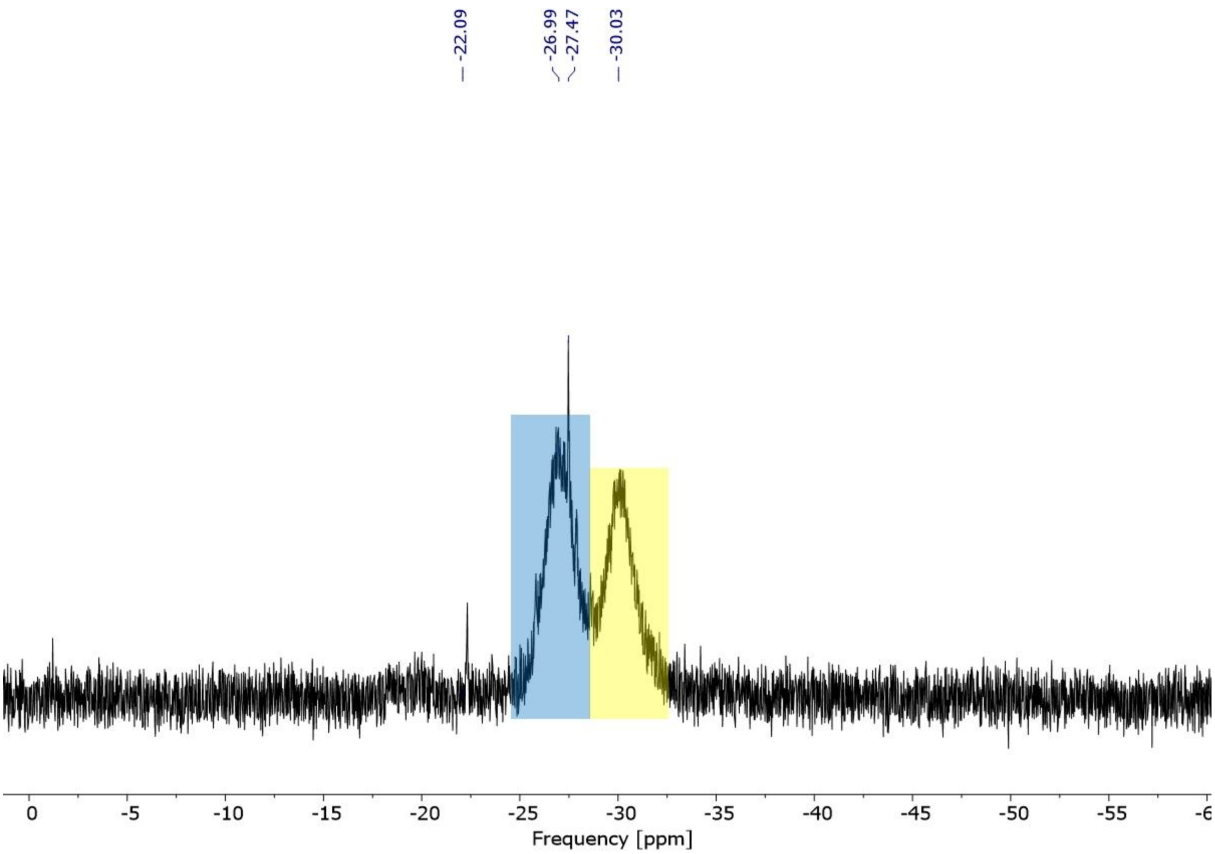

Figure S14. $^{31}P\{^{1}H\}$ NMR spectrum of **1** (243 MHz, $CDCl_3$, 298 K , blue labels = **1**, yellow label = **1'**).

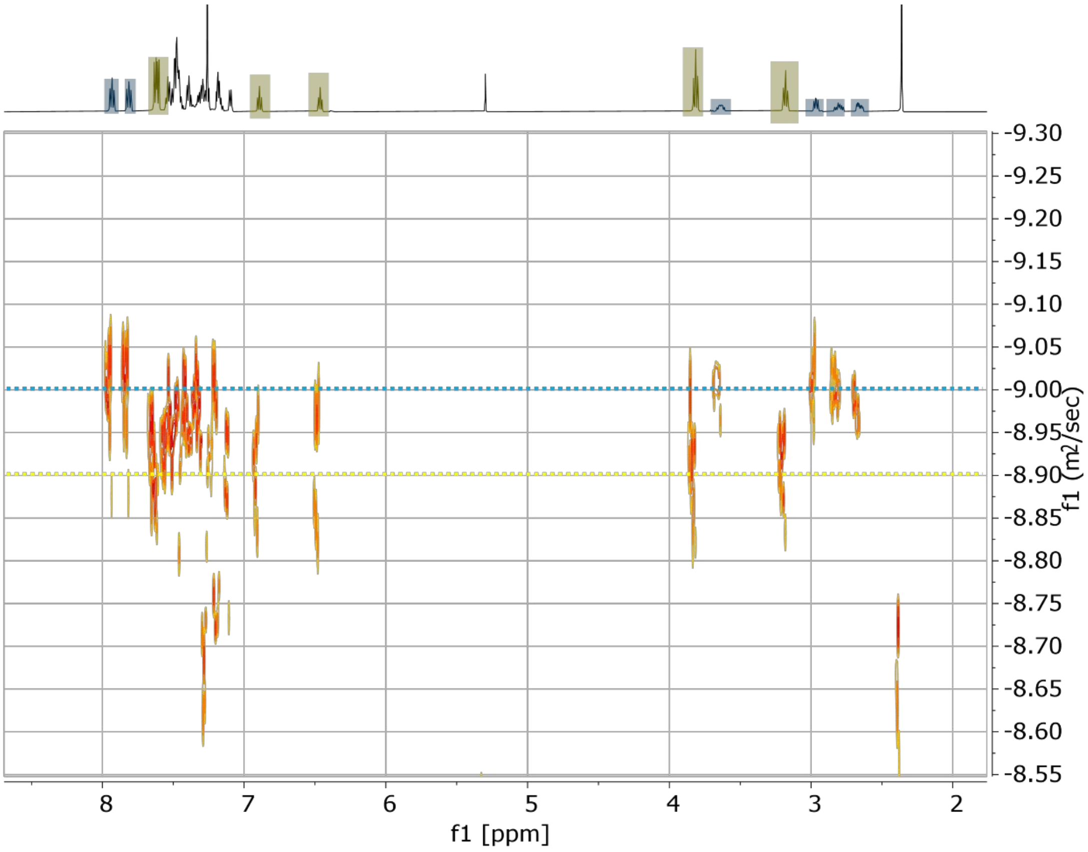

Figure S15. DOSY NMR spectrum of **1** (600 MHz, $CDCl_3$, 298 K, blue labels/line = **1**, yellow label/line = **1'**).

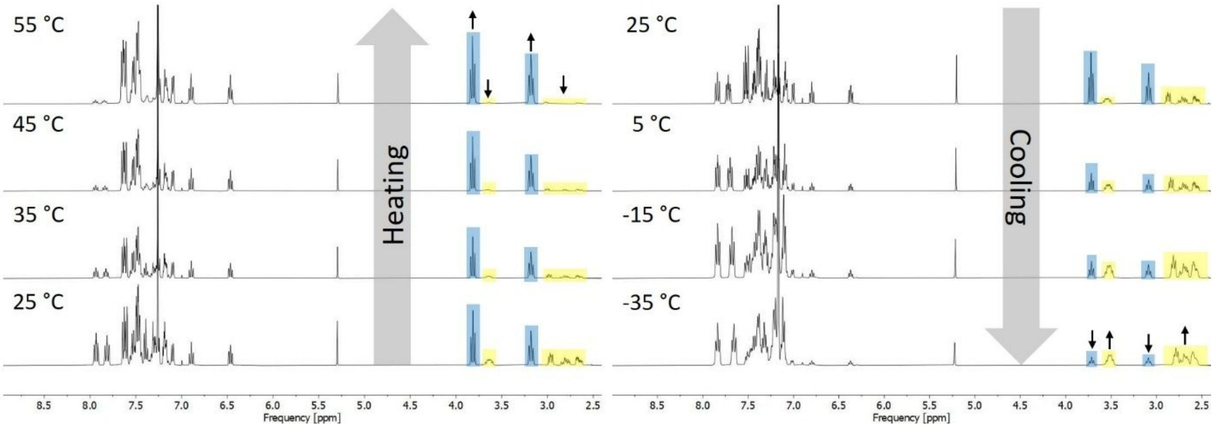

Figure S16: ^{1}H NMR (400 MHz) stack plot of **1** at variable temperature (400 MHz, $CDCl_3$, 298 K, blue labels = **1**, yellow label = **1'**).

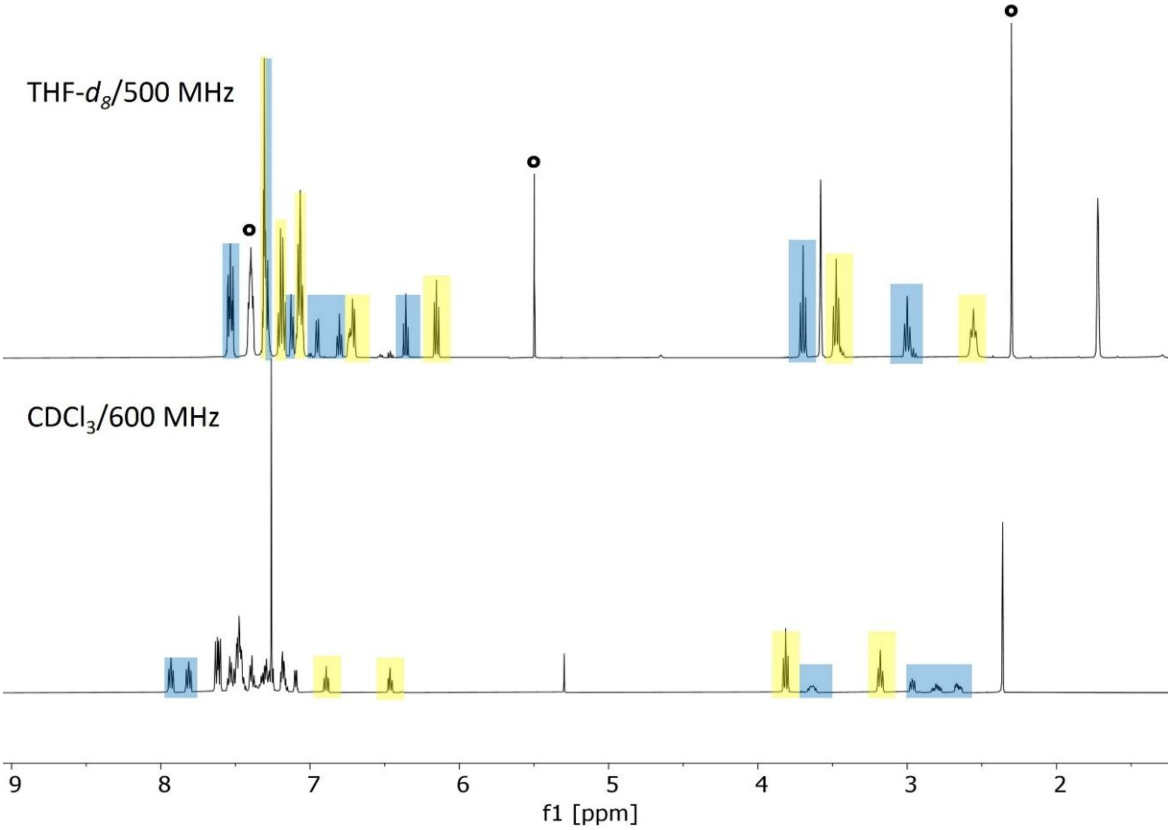

Figure S17. ^{1}H NMR stack plot of **1** in different solvents (blue labels = **1/1-THF**, yellow labels = **1*/1*-THF**, ° = co-crystallized DCM and toluene).

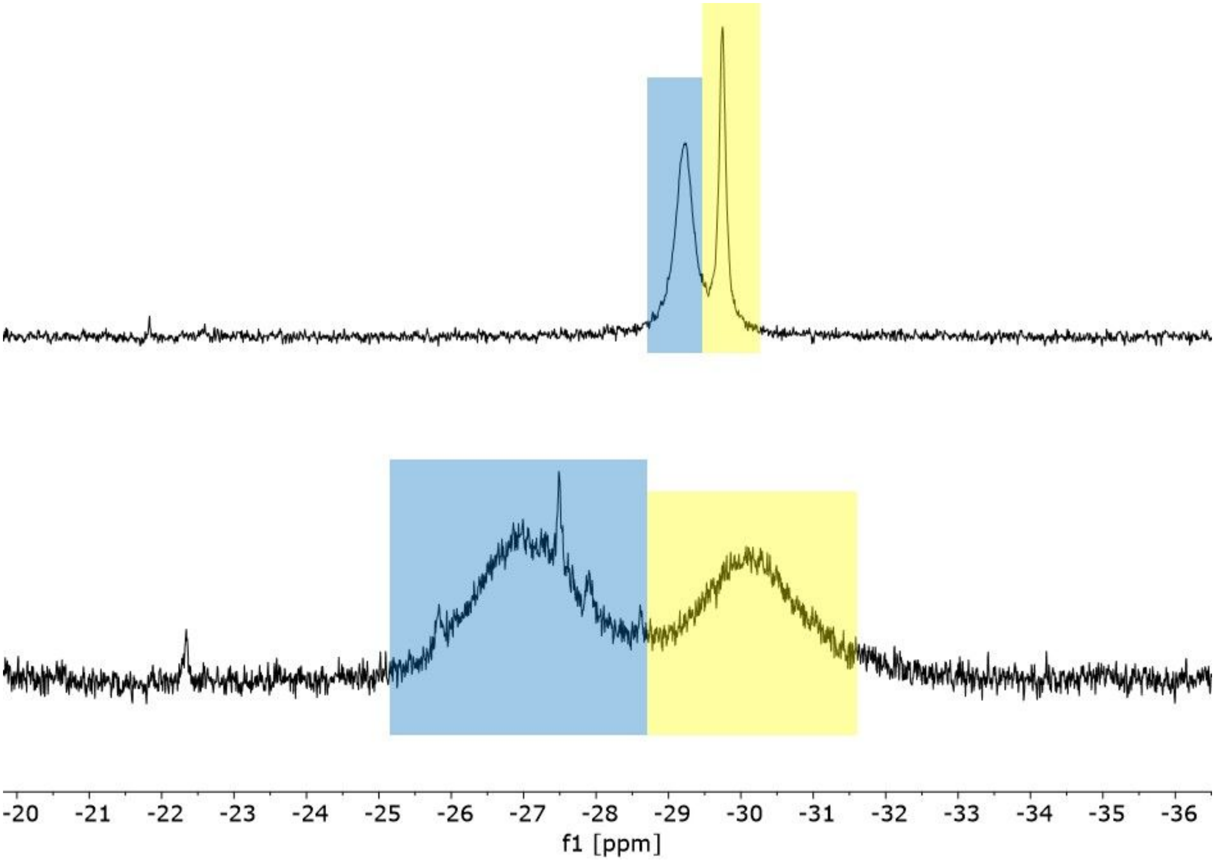

Figure S18. $^{31}P\{^{1}H\}$ NMR stack plot of **1** in different solvents (bottom $CDCl_3$, top THF-d_8, blue labels = **1/1-THF**, yellow labels = **1'/1'-THF**).

Isolation of $[Ph_2P(Ind)]_2AlCl(THF)$, 1′-THF

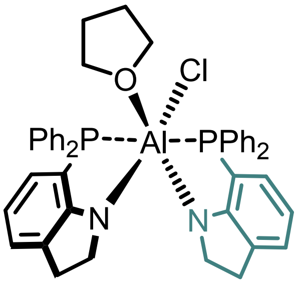

Compound **1** (30 mg, 38 µmmol) was dissolved in THF (1 mL). After stirring for 1 min, the solution was concentrated to approximately one-third of its original volume. The resulting mixture was left standing overnight, affording yellow crystals of **1′-THF**. The crystals were collected by filtration, washed with hexane, and dried under reduced pressure (11 mg, 15 µmol, 40%).

The isolated crystalline material was suitable for X-ray diffraction analysis.

^{1}H NMR (500 MHz, THF-d_8, 298 K): δ [ppm] = 7.40 (m, 4 H, *o*-PPh_2), 7.29 (m, 1 H, H_{Ar}), 7.20 (m, 1 H, H_{Ar}), 7.07 (m, 6 H, *m*-PPh_2), 6.72 (m, 2 H, *p*-PPh_2), 6.15 (m, 1 H, H_{Ar}), 3.48 (t, $^{3}J_{HH}$ = 9.0 Hz, 2 H, N$CH_2$$CH_2$), 2.56 (t, $^{3}J_{HH}$ = 9.0 Hz, 2 H, N$CH_2$$CH_2$).

^{13}C{^{1}H} NMR (125 MHz , THF-d_8, 298 K): δ [ppm] = 167.47 (br, ind-*C*-NCH_2), 135.75 (b, PhP-*C*-PPh_2), 134.32 (br,C_o-PPh_2), 132.85(*s*, C_o-ind), 132.72(s, ind-*C*-CH_2CH_2), 129.40 (s, C_m/C_p-PPh_2), 129.31 (br, C_m/C_p-PPh_2), 128.89 (br, C_m-ind), 126.63 (s, C_p-ind), 114.01 (br, *C*-PPh_2), 50.01 (s, NCH_2), 31.45 (br, N$CH_2$$CH_2$).

Due signal broadening the ^{31}P coupling constants towards the carbon atoms could not be resolved.

^{31}P{^{1}H} NMR (202 MHz, THF-d_8, 298 K): δ [ppm] = –29.74 (s).

Elemental Analysis: $C_{40}H_{34}Al_2I_4N_2P_2 \bullet (C_4H_8O)_2$ Calculated: C 70.70% H 6.62% N 3.17%

Found: C 68.68% H 6.47% N 2.97%

The crystalline material submitted for elemental analysis contained two molecules of THF per molecule of **1′**. This finding was consistent with ^{1}H NMR and XRD analysis.

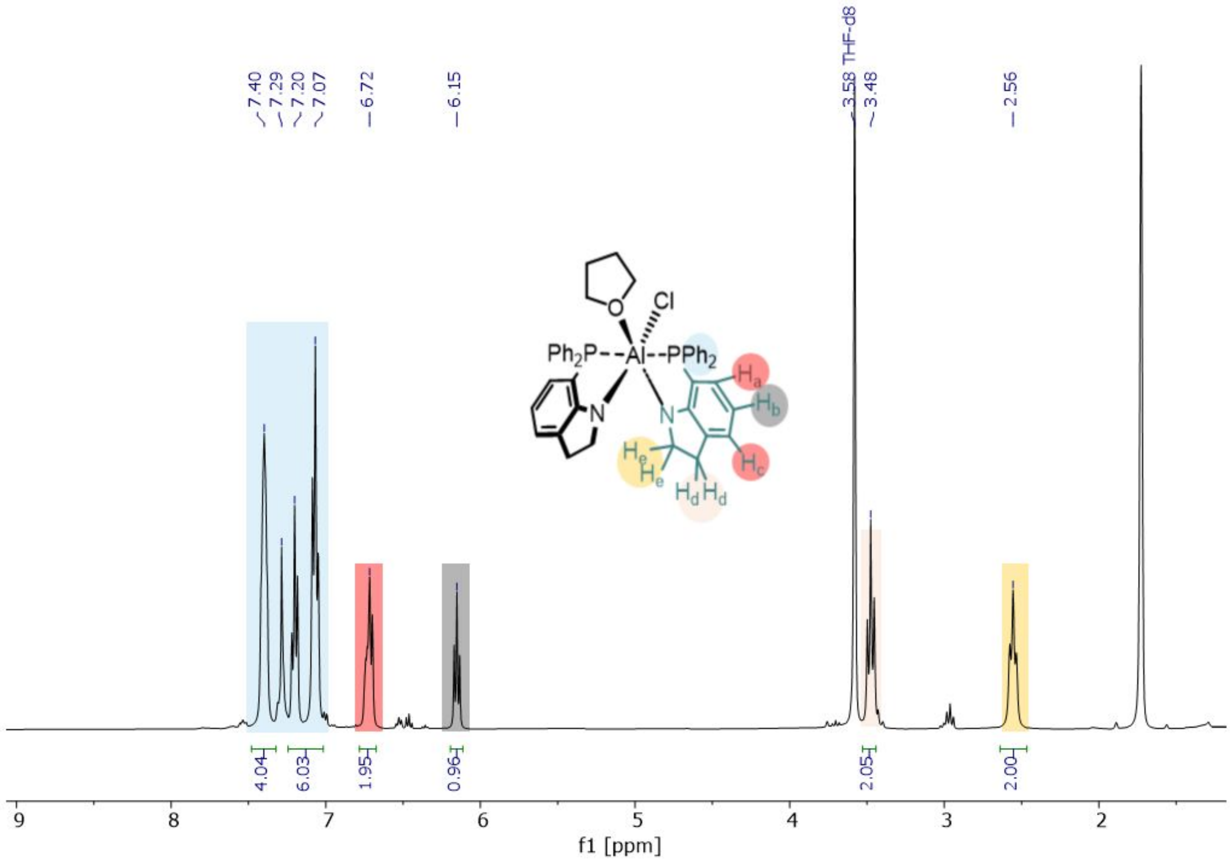

Figure S19. ^{1}H NMR spectrum of **1'-THF** (500 MHz, THF-d_8, 298 K).

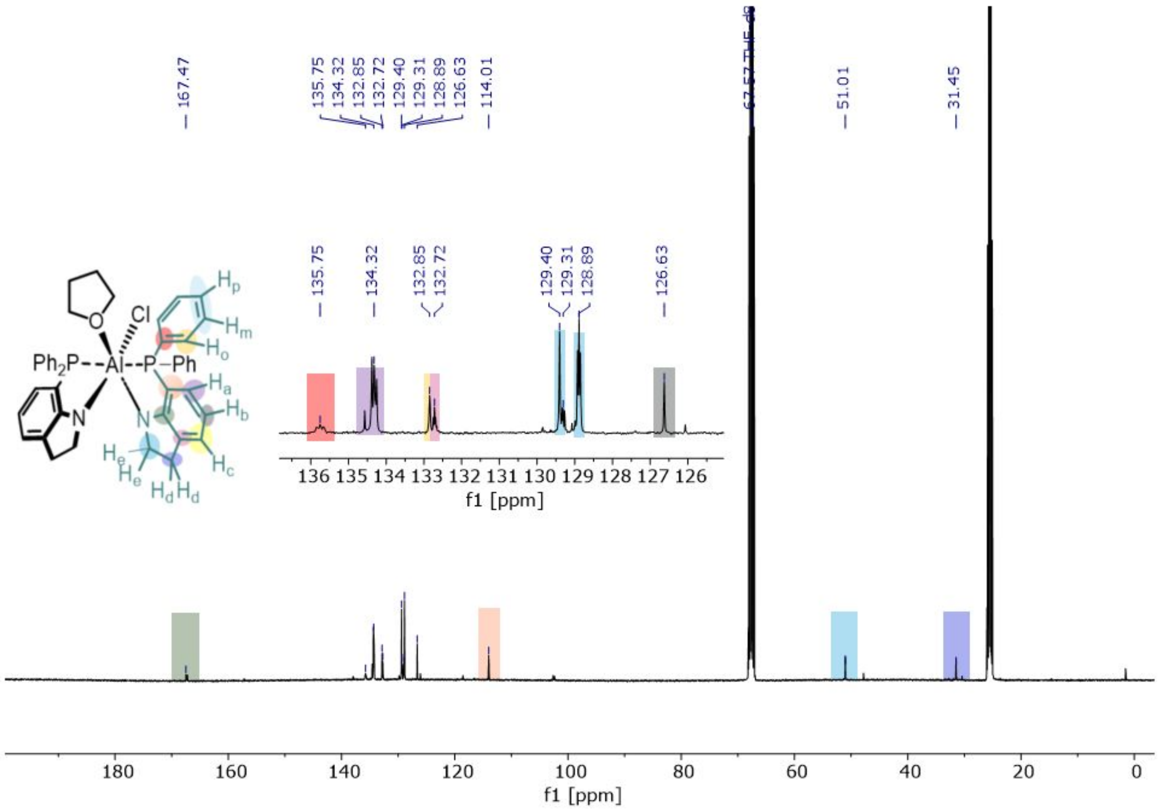

Figure S20. ^{13}C NMR spectrum of **1'-THF** (100 MHz, THF-d_8, 298 K).

2.6. Synthesis of $[Ph_2P(Ind)AlI_2]_2$, 2

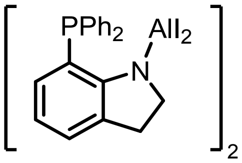

AlI_3 (236 mg, 0.58 mmol, 1.00 eq) and $Ph_2P(Ind)K$ (200 mg, 0.58 mmol, 1.00 eq) were suspended in toluene (10 mL) and stirred for 16 h at room temperature. After filtration and further extraction of the solid residue with toluene (5 mL) the combined organic phases were concentrated in vacuo to obtain a bright yellow oil. The residue was extracted with a DCM/hexane mixture. Concentration in *vacuo* led to the precipitation of a yellow solid (245 mg, 0.42 mmol, 72%) which was filtered off and dried under reduced pressure. Recrystallization via slow evaporation of a DCM/toluene mixture yielded $[Ph_2(Ind)AlI_2]$, **2**, as a colorless crystalline material.

Crystals suitable for X-ray diffraction analysis were grown from a concentrated toluene solution**.**

Note: In the course of this synthetic procedure, two different samples could be isolated depending on the purification method: fast precipitation from solution afforded a yellow powder, whereas slow crystallization gave rise to a colorless material. Elemental analysis indicated that both samples possess the molecular composition $Ph_2(Ind)AlI_2$. However, spectroscopic characterization revealed significant differences between the two samples.

XRD measurements showed that the colorless crystalline material corresponds to the dimeric compound $[Ph_2(Ind)AlI_2]_2$, **2**. Similar to its isostructural derivative **1**, NMR spectra in non-coordinating solvents indicate the presence of a second species in solution, denoted as **2'**. Due to the similarities of its NMR spectroscopic features to those of **1** and **1'**, it was assumed that **2'** corresponds to the monomeric product $Ph_2(Ind)AlI_2$.This assignment was further supported by a DOSY measurement (see Figure S26).

Analysis of the yellow powder by ^1H and ^{31}P NMR indicated that only a single species is present in solution, which resembles neither **2** or **2'**. Hence it was denoted as **2''**. DOSY measurements revealed that **2''** features a diffusion coefficient intermediate between those of **2** and **2'**. Attempts to determine the molecular structure by XRD measurements failed, as crystallization yielded compound **2** instead. Based on the results from elemental analysis it can only be assumed that **2''** represents an isomer of **2** with a different structural arrangement. Possible structures include an ionic formulation $[(Ph_2(Ind))_2Al]^+[AlI_4]^-$ or an alternative dimeric isomer. Apparently, **2''** does not undergo ligand scrambling until it converts to the more stable isomer **2** upon crystallization. Furthermore, **2** cannot be converted back to **2''**, indicating that the latter represents a metastable isomer.

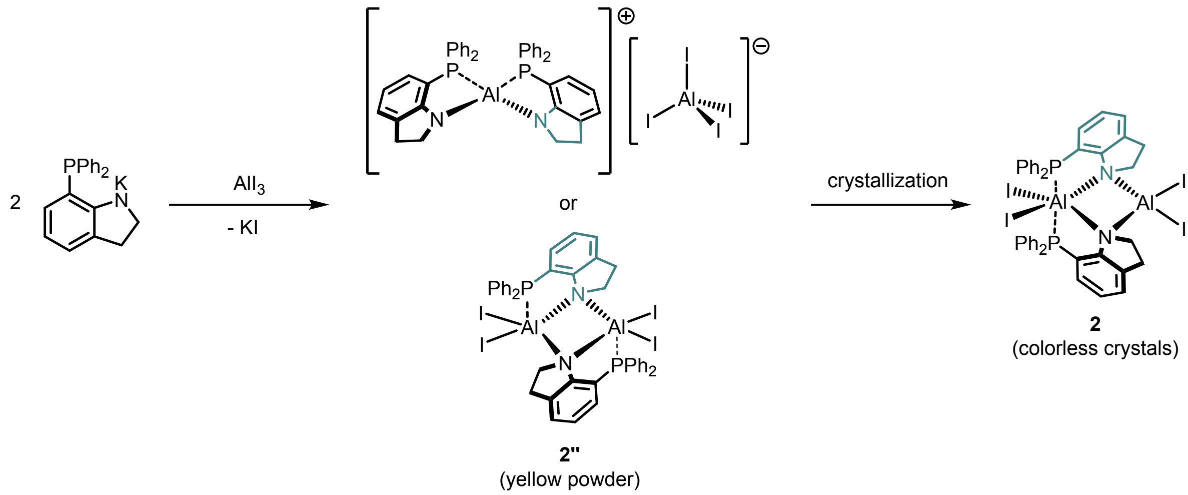

Scheme S4: Proposed formation of **2** (the shown structures for **2''** are only possible suggestions).

Analytical data obtained from the colorless crystalline sample

Elemental Analysis: $C_{40}H_{34}Al_2I_4N_2P_2$ Calculated: C 41.20% H 2.94% N 2.40%

Found: C 41.20% H 2.81% N 2.09%

Spectroscopic data for $[Ph_2P(Ind)AlI_2]_2$, **2**

^{1}H NMR (500 MHz, DCM-*d*$_2$, 298 K): δ [ppm] = 8.39 (t, 4 H, H_{PPh2}), 7.78 (t, 4 H, H_{PPh2}), 7.64 - 7.24 (overlapping multiplets of **1** and **1**′, 18 H, $H_{Ar/PPh2}$), 4.13 (m, 2 H, NCH_2CH$_2$), 3.35 (m, 2 H, NCH_2CH$_2$), 2.99 (m, 2 H, NCH$_2$$CH_2$), 2.84 (m, 2 H, NCH$_2$$CH_2$).

^{31}P{^{1}H} NMR (202 MHz, C_6D_6, 298 K): δ [ppm] = –35.18 (bs).

Spectroscopic data for $[Ph_2P(Ind)]_2AlI$, **2'**

^{1}H NMR (500 MHz, DCM-*d*$_2$, 298 K): δ [ppm] 7.64 - 7.24 (overlapping multiplets of **1** and **1**′, 22 H, $H_{Ar/PPh2}$), 6.86 (t, 4 H, H_{Ar}), 3.70 (m, 4 H, NCH_2CH$_2$), 3.16 (m, 4 H, NCH_2CH$_2$).

^{31}P{^{1}H} NMR (202 MHz, C_6D_6, 298 K): δ [ppm] = –14.88 (bs).

Due to presence of two species in solution recording of ^{13}C NMR data was not pursued. ^{31}P NMR signal have been assigned by recording a ^{1}H-^{31}P HMBC NMR spectrum.

Analytical data obtained from the yellow powder sample

Elemental Analysis: $C_{40}H_{34}Al_2I_4N_2P_2 \bullet CH_2Cl_2$ Calculated: C 39.36% H 2.90% N 2.24%

Found: C 39.30% H 2.81% N 3.16%

The crystalline material submitted for elemental analysis contained one molecule of DCM per molecule of **2''**. This finding was consistent with ^{1}H NMR analysis.

Spectroscopic data for **2''**

^{1}H NMR (500 MHz, DCM-d_2, 298 K): δ [ppm] = 7.65(m, 4 H, *o*-PPh$_2$), 7,58 (m, 2 H, $H_{p\text{-}PPh2}$), 7,50(m, 4 H, $H_{m\text{-}PPh2}$), 7.13 (m, 1 H, H_{Ar}), 6.92 (m, 1 H, H_{Ar}), 6.49 (m, 1H, H_{Ar}), 3.72 (t, 2 H, NCH_2CH), 3.18 (t, $^3J_{HH}$ = 8.8 Hz, 2H, NCH_2CH$_2$)

^{31}P{^{1}H} NMR (167 MHz, DCM-d_2, 298 K): δ [ppm] = –30.00 (bs).

Due to the uncertain structural constitution of **2''** recording of ^{13}C NMR data was not pursued. Furthermore, the signals in the ^{1}H NMR spectrum were integrated assuming one indoline ligand per molecule, although alternative structures containing multiple ligands molecular unit were also considered.

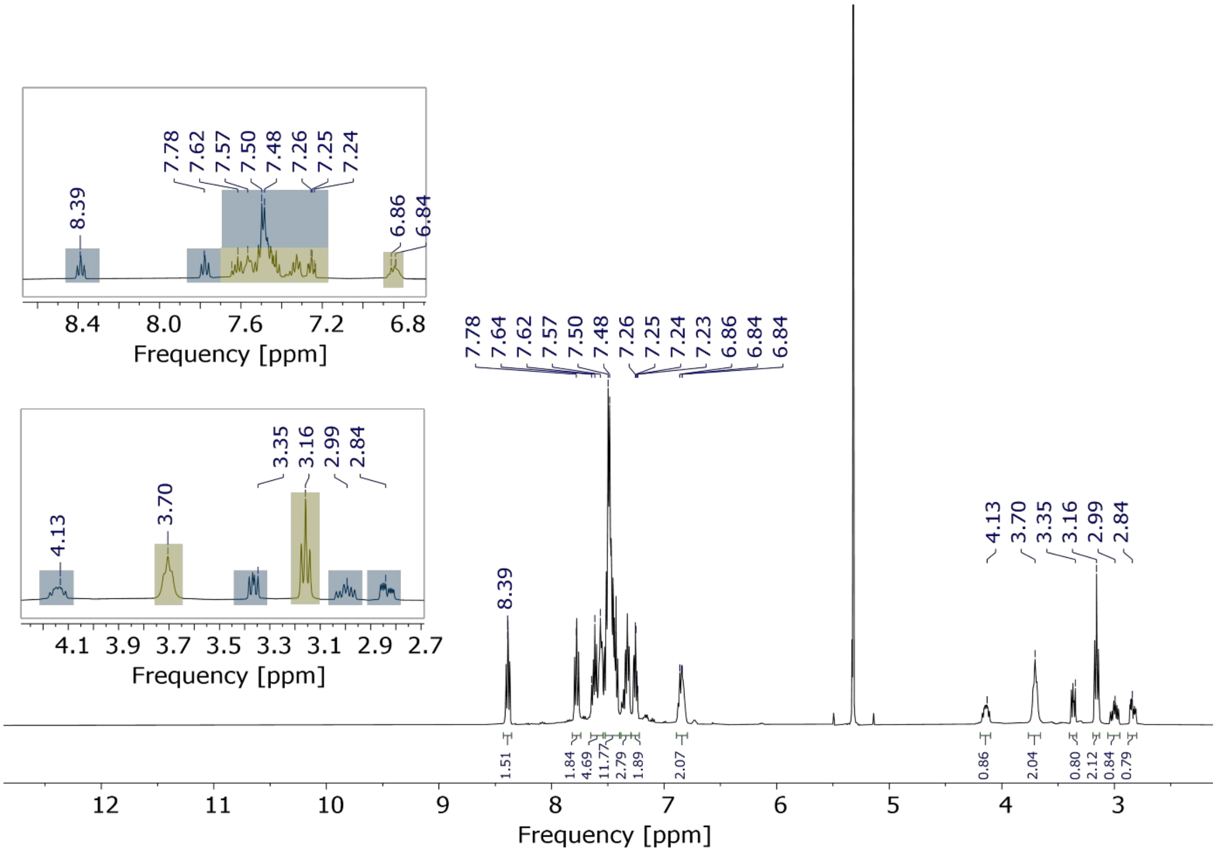

Figure S21. ^{1}H NMR spectrum of **2** (500 MHz, DCM-d_2, 298 K, blue labels = **2**, yellow label = **2'**).

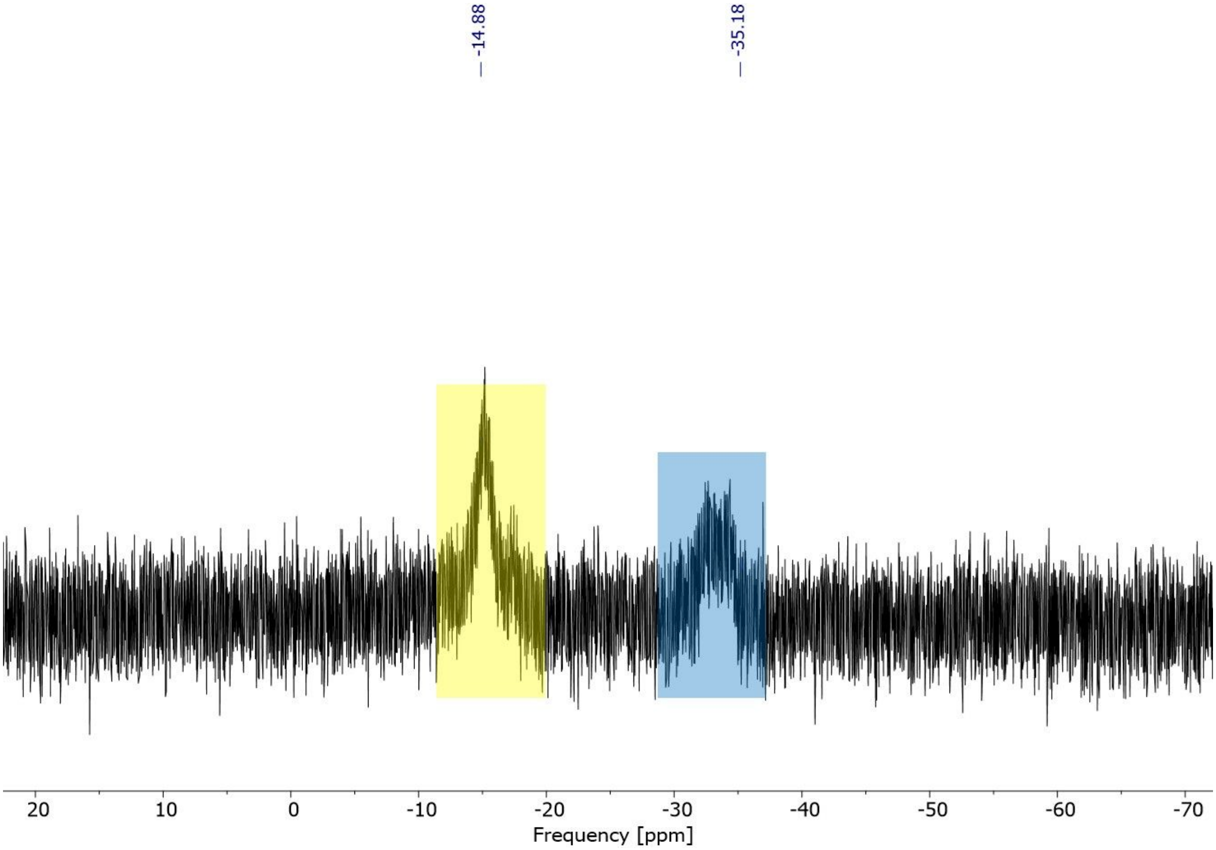

Figure S22. $^{31}P\{^{1}H\}$ NMR spectrum of **2** (243 MHz, DCM-d_2, 298 K, blue labels = **2**, yellow label = **2'**).

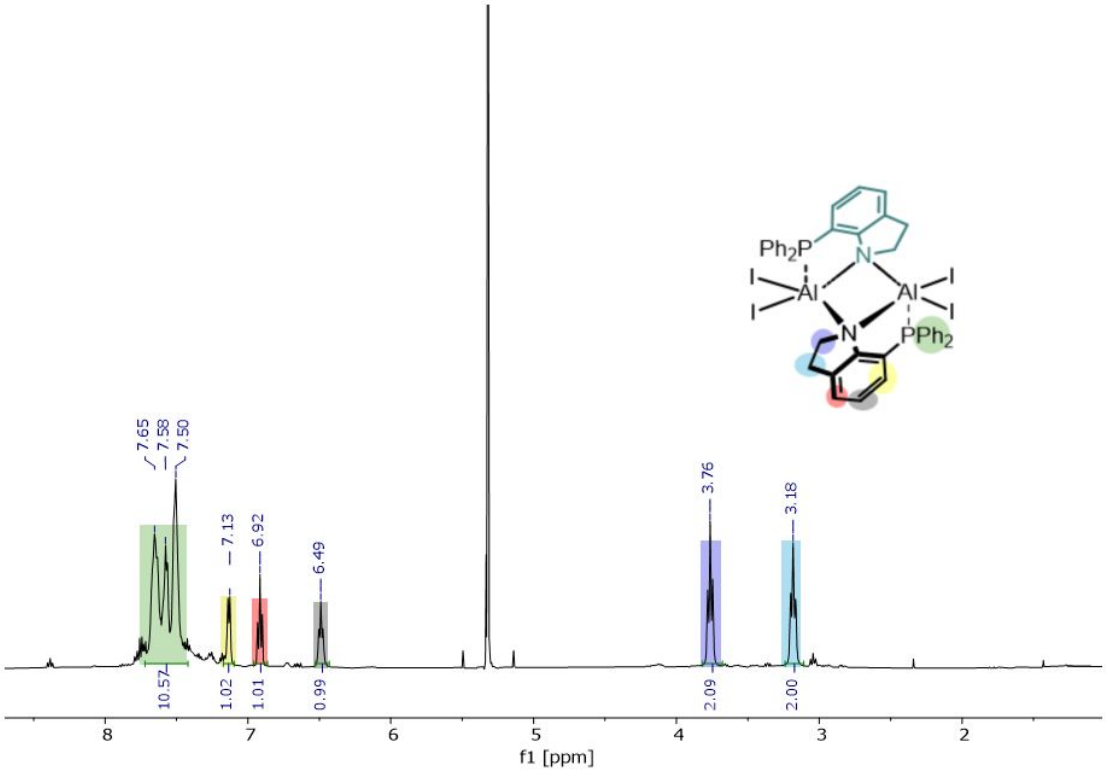

Figure S23. ^{1}H NMR spectrum of **2''** (500 MHz, DCM-d_2, 298 K).

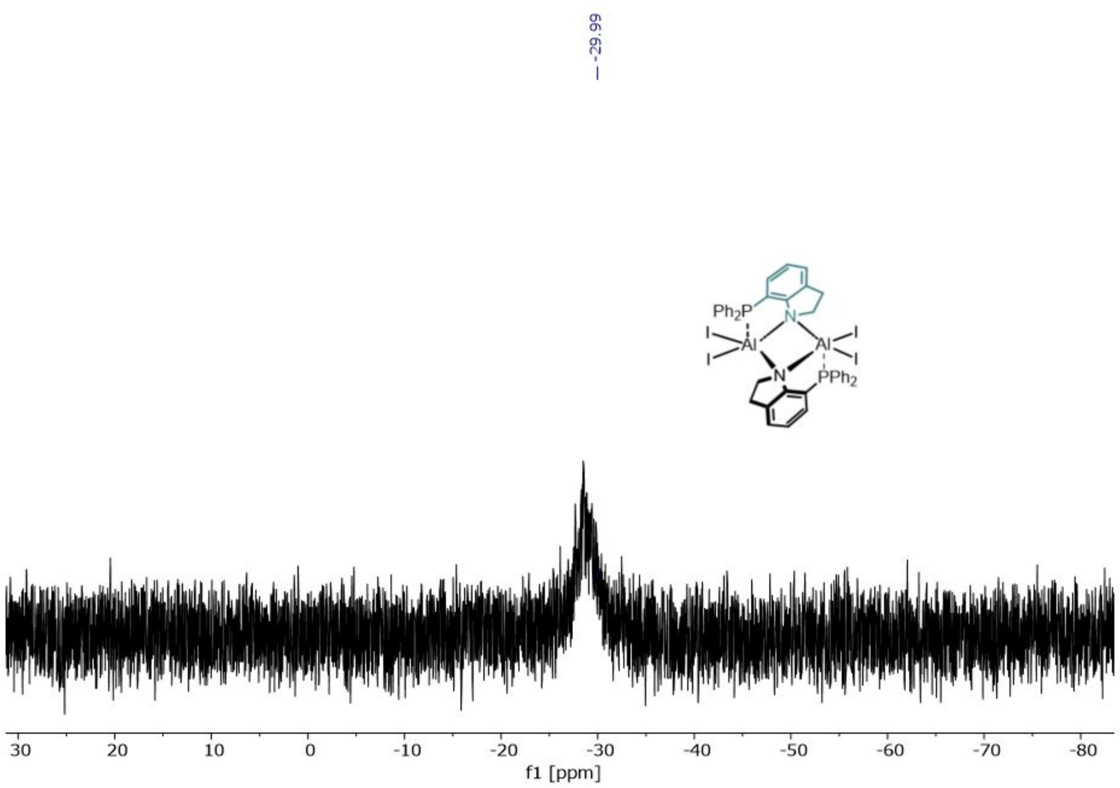

Figure S24: $^{31}P\{^{1}H\}$ NMR spectrum of **2''** (162 MHz, DCM-d_2, 298 K).

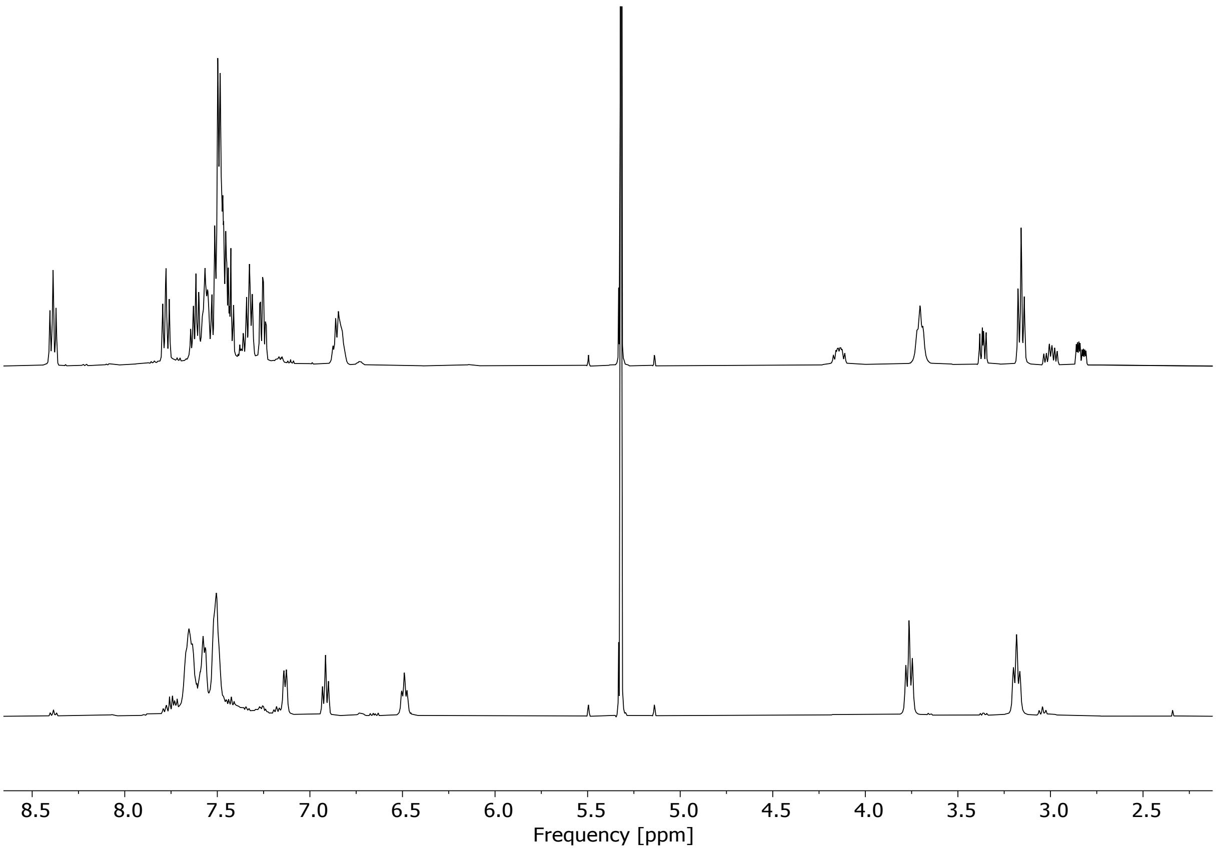

Figure S25. ^{1}H NMR stack plot of **2/2'** (top) and **2''** (bottom) (500 MHz, DCM-d_2, 298 K).

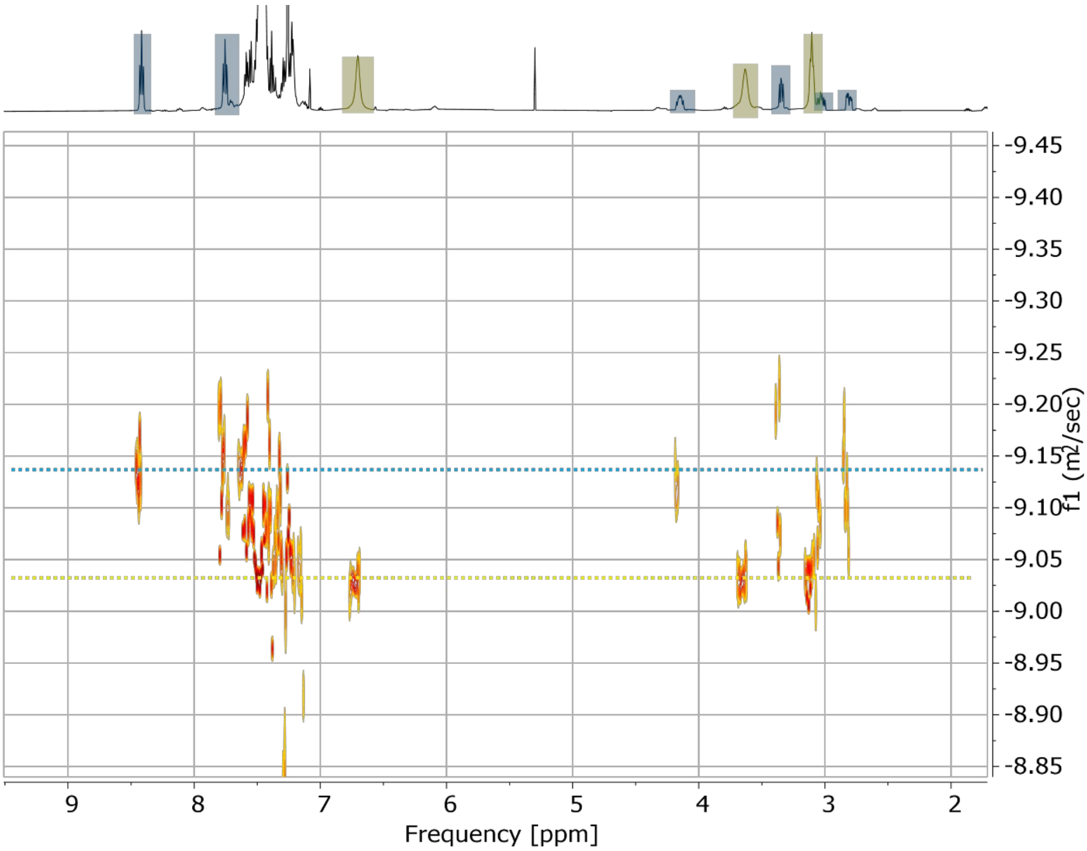

Figure S26. DOSY NMR spectrum of **2** (600 MHz, $CDCl_3$, 298 K, blue labels/line = **2**, yellow label/line = **2'**).

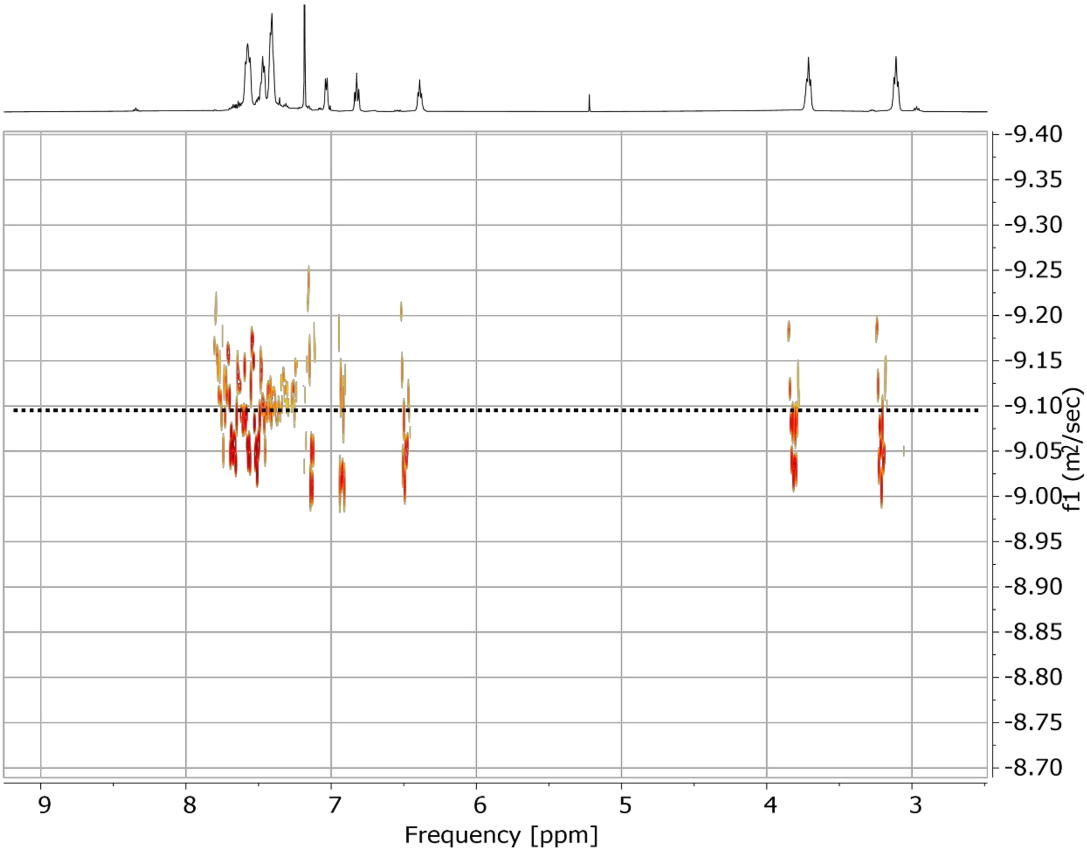

Figure S27. DOSY NMR spectrum of **2''** (600 MHz, $CDCl_3$, 298 K).

2.7. Synthesis of $Ph_2P(Ind)AlMe_2$, 3

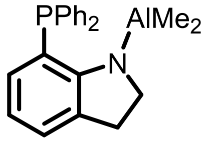

$Ph_2P(Ind)H$ (1.00 g, 3.29 mmol, 1.00 eq) was dissolved in toluene (10 mL) and $AlMe_3$ (1.64 mL, 2.00 M in toluene, 3.29 mmol, 1.00 eq) was added slowly. The reaction mixture immediately turned lime green and was stirred for 2 h at room temperature. The solvent was removed *in vacuo.* The residue was washed with 5 mL of hexane and dried under high *vacuo* for 1 h to yield $Ph_2P(Ind)AlMe_2$, **3**, (895 mg, 2.49 mmol, 76%) as a lime green compound.

Crystals suitable for X-ray diffraction analysis were grown by layering a concentrated DCM solution with a threefold excess of hexane.

^{1}H NMR (300 MHz, C_6D_6, 298 K): δ [ppm] = 7.43-7.36 (m, 4H, *o*-PPh_2), 6.96-6.91 (m, 6H, *m*-PPh_2 and *p*-PPh_2), 6.90-6.83 (m, 2H, H_a, H_c), 6.34 (ddd, $^3J_{HH}$ = 8.1 Hz, $^3J_{HH}$ = 6.8 Hz, $^4J_{HP}$ = 1.5 Hz 1H, H_b), 3.62 (t, $^3J_{HH}$ = 8.8 Hz, 2H, H_e), 2.88 (t, $^3J_{HH}$ = 8.8 Hz, 2H, H_d), -0.20 (d, $^3J_{HP}$ = 4.1 Hz, 6 H, $AlMe_2$).

^{13}C{^{1}H} NMR (75 MHz, C_6D_6, 298 K): δ [ppm] = 168.47 (d, $^2J_{CP}$ = 14.25 Hz, ind-*C*-NCH_2), 133.31 (d, $^1J_{CP}$ = 7.5 Hz, PhP-*C*-C_5H_5), 131.47 (d, $^2J_{CP}$ = 11.25 Hz, *C*-H_o), 130.74 (*C*- H_a), 130.46 (s, ind-*C*-CH_2CH_2), 129.62 (s, *C*- H_m/ H_p), 129.14 (s, *C*- H_m/ H_p), 129.06 (s, *C*-H_c), 114.54 (d, $^3J_{CP}$ = 3.75 Hz, *C*-PPh_2), 98.65 (d, $^1J_{CP}$ = 25.75 Hz, *C*-H_b), 48.42 (d, $^4J_{CP}$ = 2.21 Hz, *C*-H_e), 31.84 (d, $^4J_{CP}$ = 2.25 Hz, *C*-H_d), –9.97 (d, $^2J_{CP}$ = 11.25 Hz, $AlMe_2$).

^{31}P{^{1}H} NMR (121 MHz, C_6D_6, 298 K): δ [ppm] = –19.39 (s).

Elemental Analysis: $C_{22}H_{23}AlNP$ Calculated: C 73.53% H 6.45% N 3.90%

Found: C 72.99% H 6.55% N 3.92%

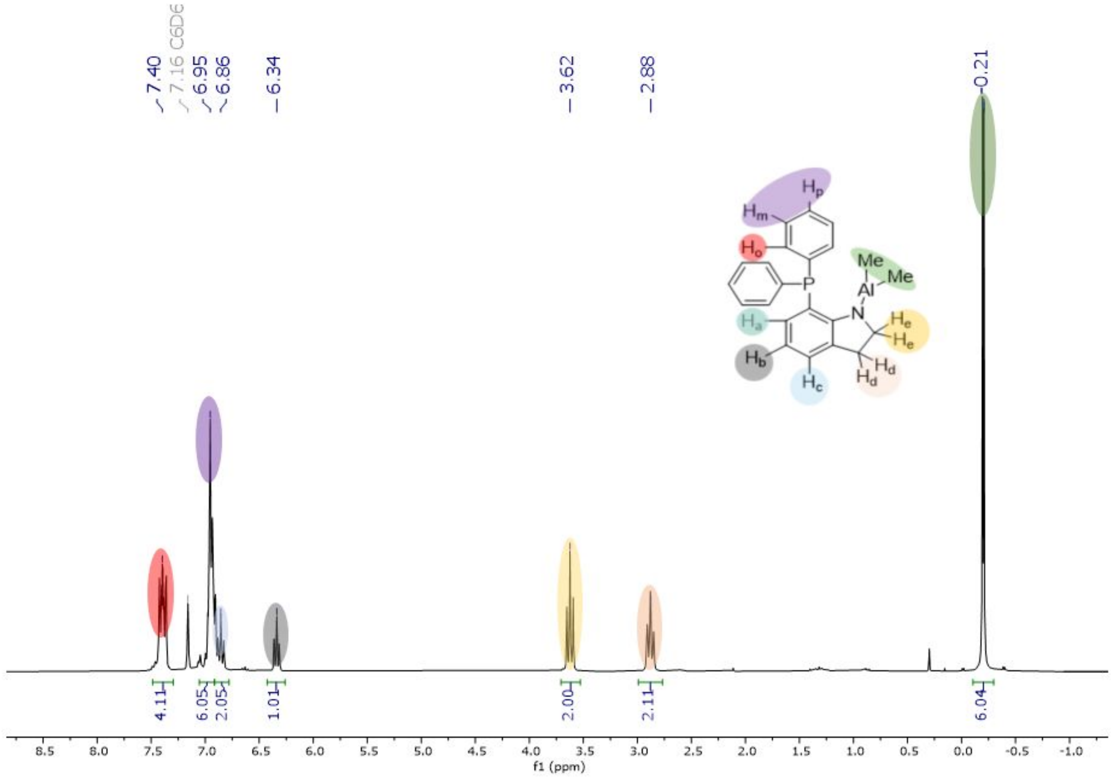

Figure S28. ^{1}H NMR spectrum of **3** (300 MHz, C_6D_6, 298 K).

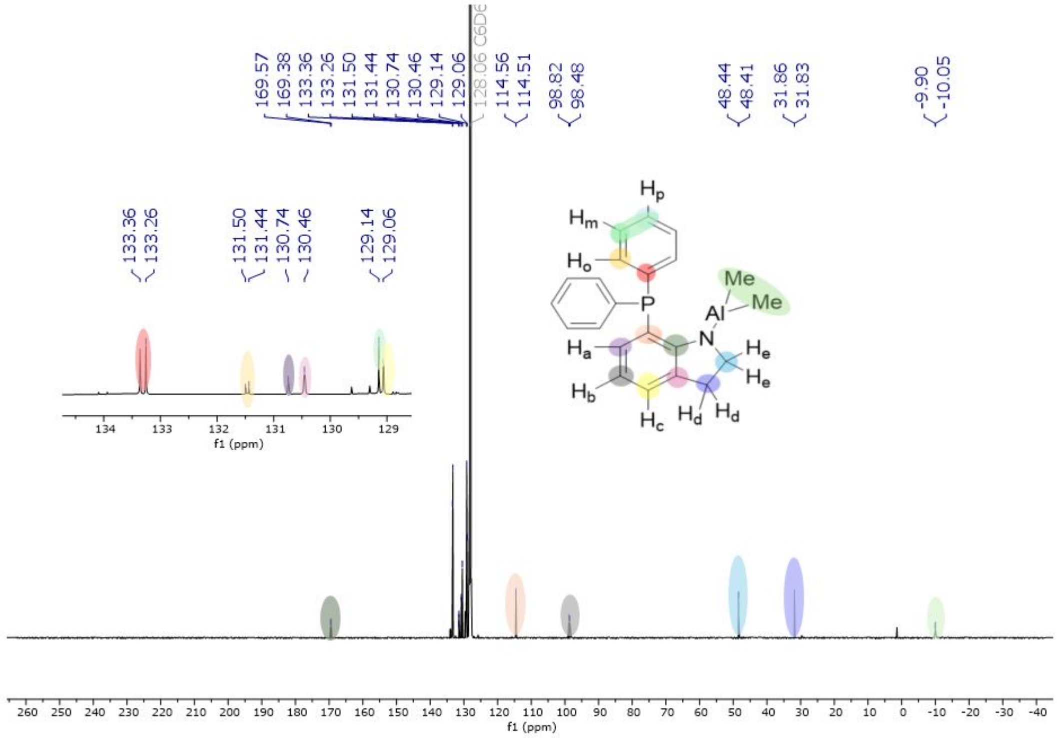

Figure S29. $^{13}C\{^{1}H\}$ NMR spectrum of **3** (75 MHz, C_6D_6, 298 K).

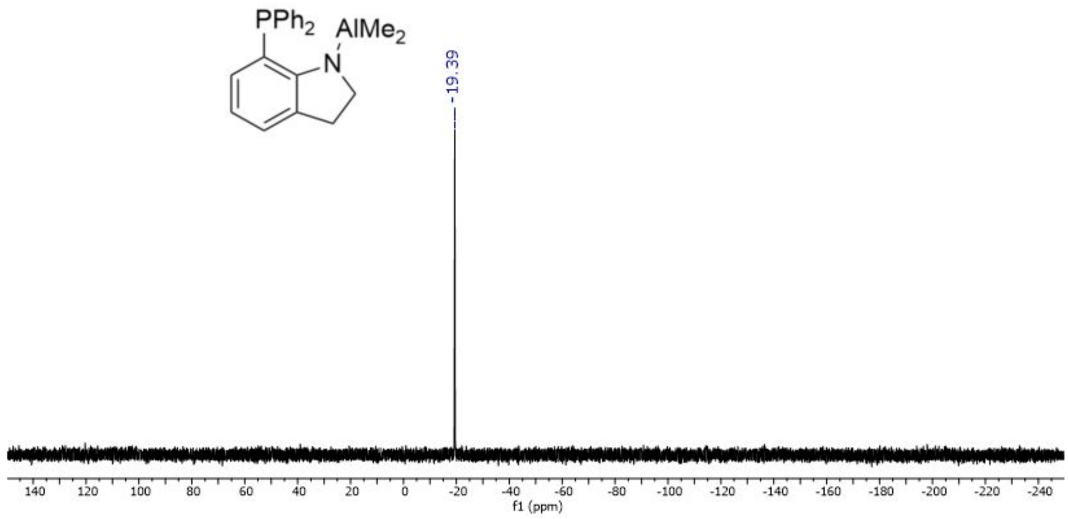

Figure S30. $^{31}P\{^{1}H\}$ NMR spectrum of **3** (121 MHz, C_6D_6, 298 K).

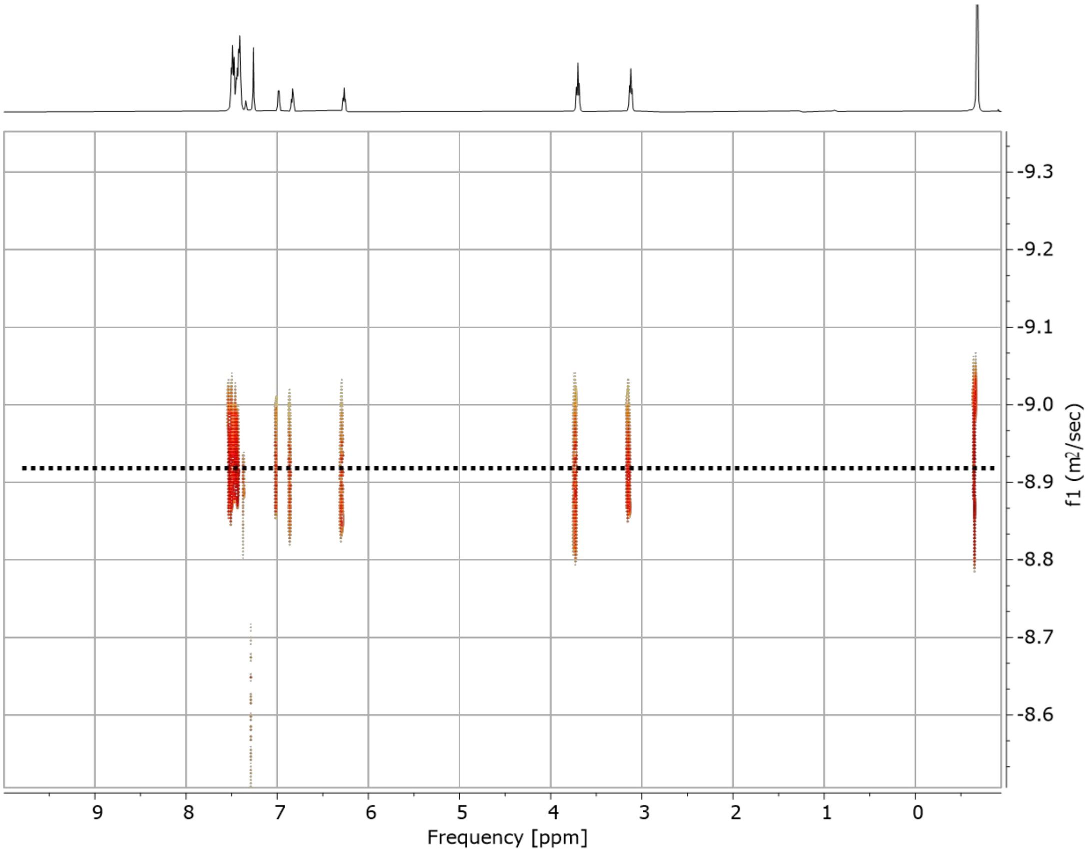

Figure S31. DOSY NMR spectrum of **3** (600 MHz, $CDCl_3$, 298 K).

2.8. Synthesis of $Ph_2P(Ind)Al(C_6F_5)_2$, 4

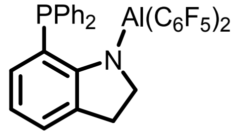

Method A

$Ph_2P(Ind)K$ (177 g, 0.52 mmol, 1.00 eq) and $(C_6F_5)_2AlCl$ (205 mg, 0.52 mmol, 1.00 eq) were suspended in toluene (5 mL) and stirred overnight. After removal of the solvent the residue was extracted with toluene (3x 5 mL). The combined organic phases were concentrated to about 2 mL and stored for crystallization overnight. The bright yellow crystalline material was filtered off, washed with hexane and dried under reduced pressure to afford $Ph_2P(Ind)Al(C_6F_5)_2$, **4** (124 mg, 0.19 mmol, 37%).

Method B

$MeAl(C_6F_5)_3$ (0.2 mL, 0.5 M in toluene, 0.01 mmol, 1.00 eq) was added to a toluene solution of **$Ph_2P(Ind)H$** (30 mg, 0.1 mmol, 1.00 eq) and the mixture was refluxed for 2 days. After removal of the solvent the bright yellow residue was washed with small portions of hexane, dried under reduced pressure and was used without any further purification.

Crystals suitable for X-ray diffraction analysis were grown via slow evaporation of a benzene solution.

Elemental Analysis: $C_{32}H_{17}AlF_{10}NP$ Calculated: C 57.93% H 2.58% N 2.11%

Found: C 58.90% H 2.69% N 2.14%

Spectroscopic data for $Ph_2P(Ind)Al(C_6F_5)_2$, **4**

^{1}H NMR (400 MHz, C_6D_6, 298 K): δ [ppm] = 7.26 (m, 4 H, H_{PPh2}), 7.08 – 6.75 (overlapping multiplets of **4** and **4'**, 22 H, $H_{Ar/PPh2}$), 6.72 (m, 1 H, H_{Ar}), 6.36 (m, 1 H, H_{Ar}), 3.89 (m, 2 H, N$CH_2$$CH_2$), 2.95 (overlapping multiplets of **4** and **4'**, 2 H, N$CH_2$$CH_2$).

^{31}P{^{1}H} NMR (162 MHz, C_6D_6, 298 K): δ [ppm] = –19.61 (bs).

^{19}F NMR (376 MHz, C_6D_6, 298 K): δ [ppm] = – 121.06 (m, 4 F, *o*-F), – 152.27 (t, $^3J_{FF}$ = = 19.7 Hz, 2 F, *p*-F), –161.10 (m, 4 F, *m*-F).

Note: Elemental analysis of the crystalline material confirmed the expected molar ratio for $Ph_2P(Ind)Al(C_6F_5)_2$, indicating purity of the sample. However, analysis by ^{1}H and ^{31}P NMR spectroscopy revealed the presence of two species in solution (see Figures S32 and S33), even after multiple recrystallization cycles. Variable-temperature NMR studies were therefore conducted to investigate whether 4 undergoes a monomer–dimer equilibrium in solution, analogous to that observed for 1. In contrast to 1, no changes in the relative signal integrals were observed upon heating or cooling, indicating that no temperature-dependent equilibrium is present.

Although slow evaporation of benzene solutions typically afforded single crystals of 4, one crystallization experiment unexpectedly yielded single crystals of $[Ph_2P(Ind)]_2Al(C_6F_5)$, **4***(see

crystallographic section). These findings suggest that 4 undergoes ligand scrambling in solution (see crystallographic section). Accordingly, the major species observed in solution was assigned to compound 4, whereas the minor species was tentatively attributed to the scrambling product $[Ph_2P(Ind)]_2Al(C_6F_5)$, **4'**. Unfortunately, **4'** could not be isolated in sufficient quantities to allow independent characterization by NMR spectroscopy.

Attempts to verify this assignment by DOSY NMR spectroscopy were unsuccessful because both species exhibit very similar diffusion constants (see Figure S35). However, during determination of the acceptor number of **4**, the addition of Et_3PO resulted in NMR spectra displaying only a single set of signals, indicating that **4** and **4'** quantitatively converge to the adduct $Ph_2P(Ind)Al(C_6F_5)_2(Et_3PO)$ (**4-Et₃PO**) (see Section 3).

It should be noted that, although several experimental observations support the proposed scrambling behavior of compound **4**, the corresponding scrambling by-product $Al(C_6F_5)_3$ could not be detected by ^{19}F NMR spectroscopy (see Figure S34).

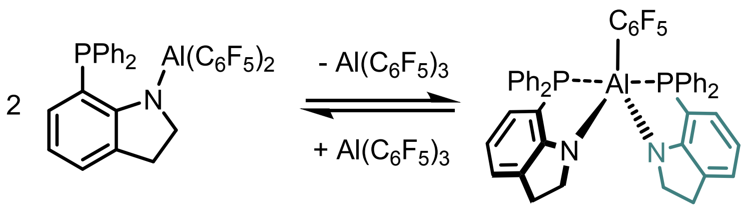

4 **4'**

Scheme S5: Proposed ligand scrambling of **4**.

Spectroscopic data for $[Ph_2P(Ind)]_2Al(C_6F_5)$, **4'**

^{1}H NMR (600 MHz, $CDCl_3$, 298 K): δ [ppm] = 7.33 (m, 4 H, H_{PPh2}), 7.08 - 6.75 (overlapping multiplets of **4** and **4'**, 22 H, $H_{Ar/PPh2}$), 6.89 (t, 2 H, H_{Ar}), 6.61 (m, 1 H, H_{Ar}), 6.42 (m, 1 H, H_{Ar}), 3.89 (t, 2 H, NCH_2CH₂), 2.95 (overlapping triplets of **4** and **4'**, 2 H, NCH_2CH₂).

^{31}P{^{1}H} NMR (243 MHz, $CDCl_3$, 298 K): δ [ppm] = –24.57 (bs).

Due to presence of two species in solution recording ^{13}C NMR data was not pursued. ^{31}P NMR signal have been assigned by recording a ^1H-^{31}P HMBC NMR spectrum.

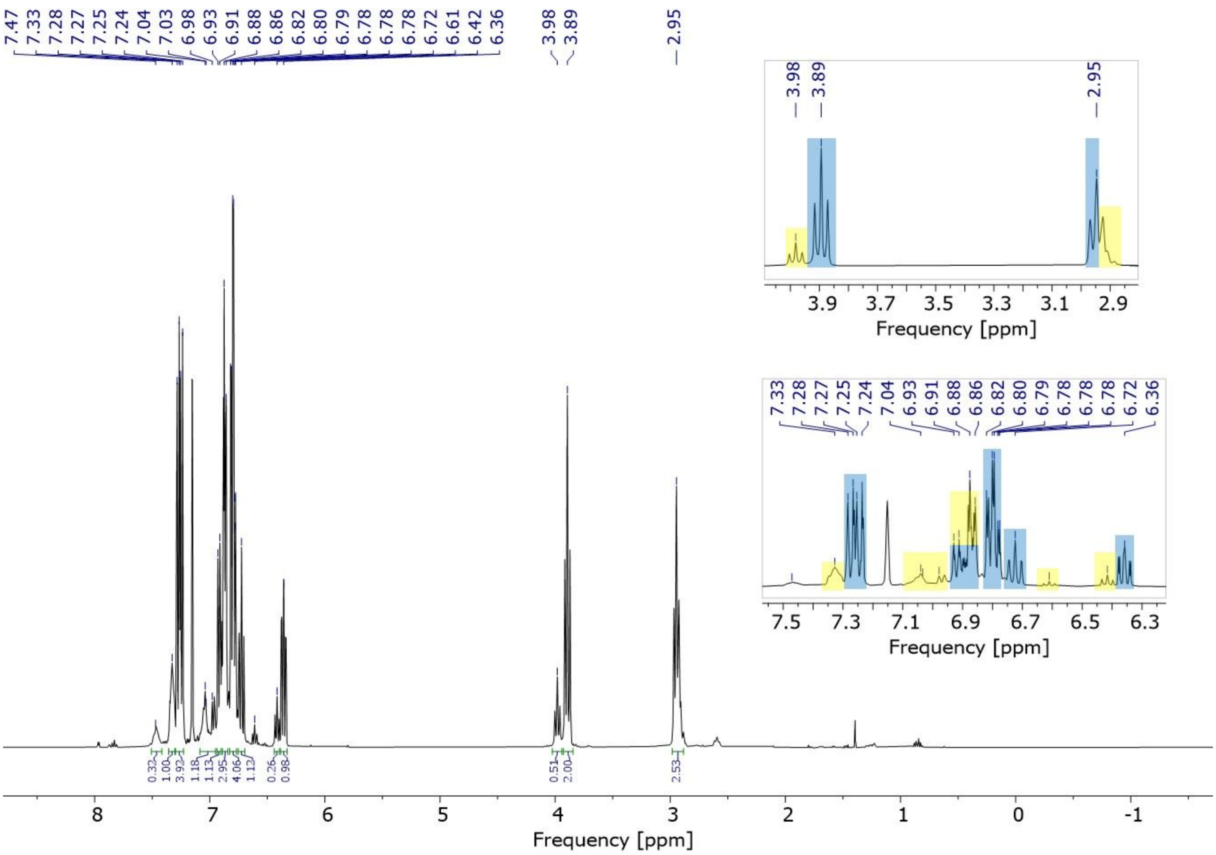

Figure S32. ^{1}H NMR spectrum of **4** (300 MHz, CD_2Cl_2, 298 K, blue labels = **4**, yellow label = **4'**).

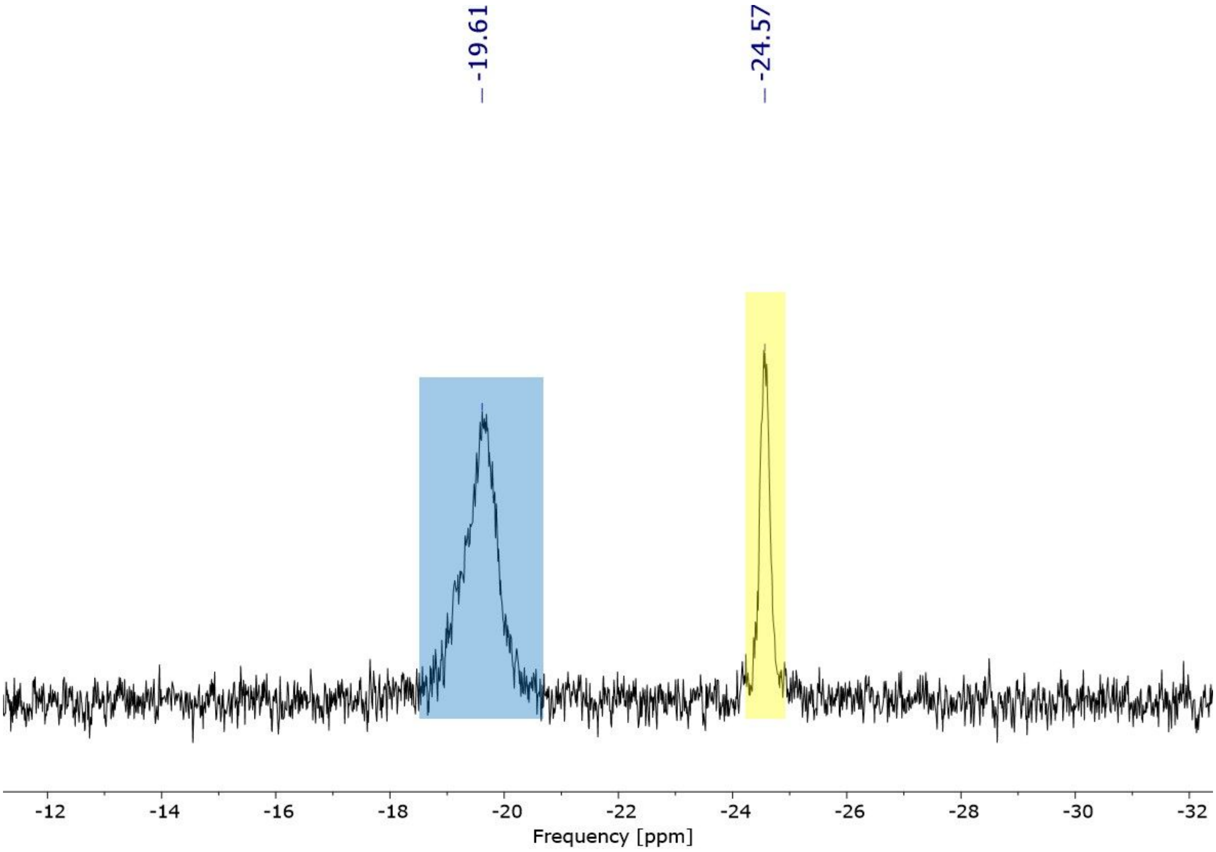

Figure S33. ^{31}P NMR spectrum of **4** (121 MHz, CD_2Cl_2, 298 K, blue labels = **4**, yellow label = **4'**).

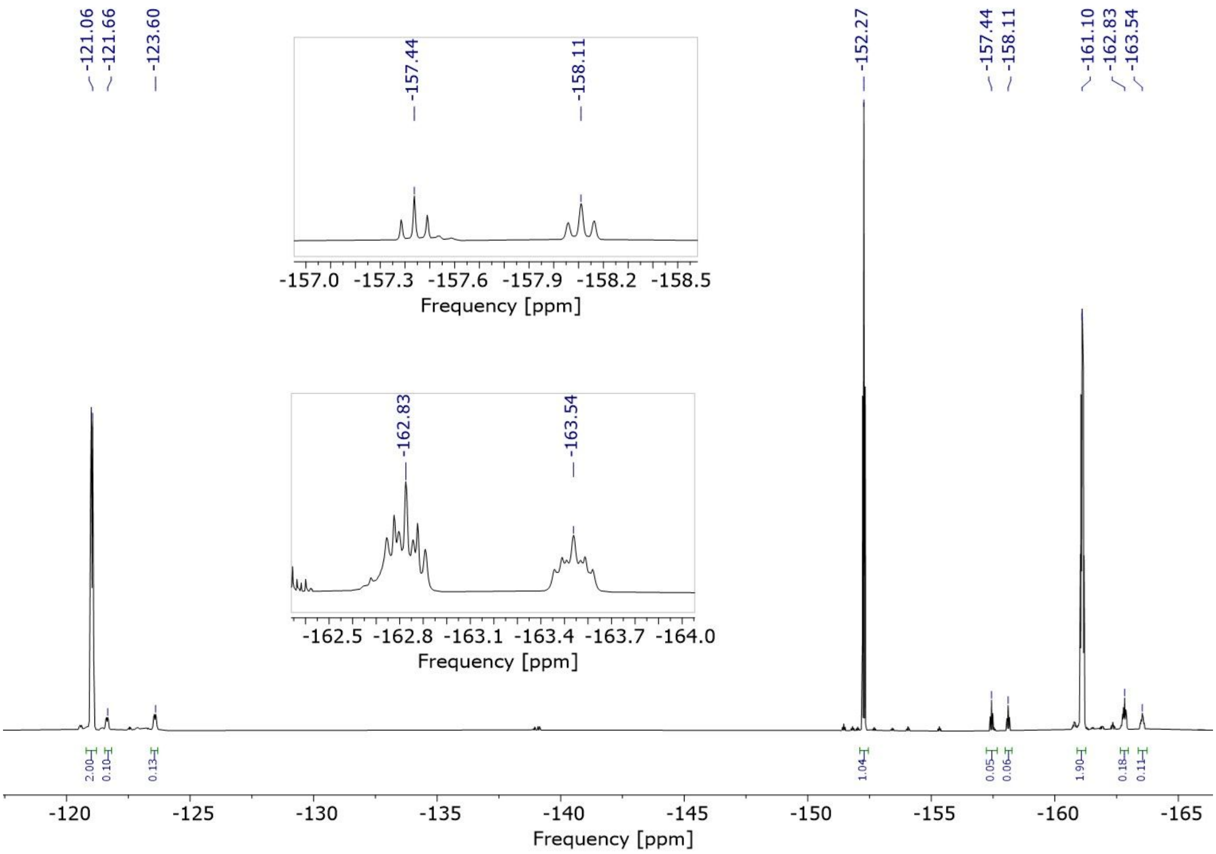

Figure S34. ^{19}F NMR spectrum of **4** (282 MHz, CD_2Cl_2, 298 K).

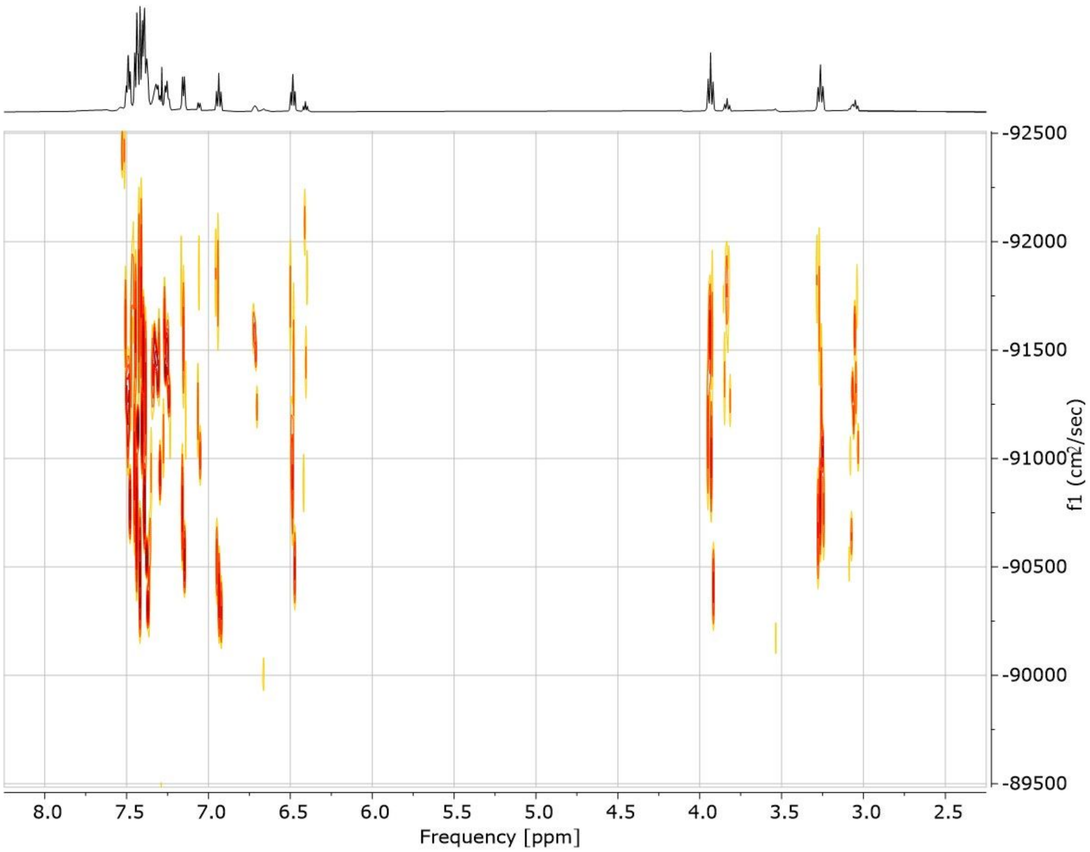

Figure S35. ^{19}F NMR spectrum of **$Ph_2P(Ind)Al(C_6F_5)_2$, 4** (282 MHz, CD_2Cl_2, 298 K).

2.9. Synthesis of $Ph_2P(Ind)Al(Me)_2(Ph_2CN_2)$, 3-CNN

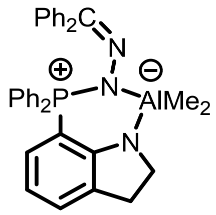

$Ph_2P(Ind)AlMe_2$ (100 mg, 0.28 mmol, 1.00 eq) was dissolved in toluene (5 mL). A solution of diphenyldiazomethane (54 mg, 0.28 mmol, 1.00 eq) in 3 ml toluene was added slowly and stirred for 1 h at room temperature. The solvent was removed *in vacuo.* The residue was washed with 5 mL of hexane and dried under high *vacuo* for 2 h to yield $Ph_2P(Ind)Al(Me)_2(Ph_2CN_2)$, **3-CNN**, (85 mg, 0.15 mmol, 55%) as a yellow powder.

Crystals suitable for X-ray diffraction analysis were grown from concentrated benzene solution.

^{1}H NMR (300 MHz, C_6D_6, 298 K): δ [ppm] = 7.88-7.80 (m, 4H, *o*-PPh_2), 7.48-7.46 (m, 2H, *p*-PPh_2), 7.25-7.18 (m, 4H, *m*-PPh_2), 7.02-6.93 (m, 10H, *C*-PPh_2), 6.84-6.82 (m, 1H, H_a), 6.65-6.58 (m, 1H, H_c), 6.22-6.16 (m, 1H, H_b), 3.66 (t, $^3J_{HH}$ = 8.9 Hz, 2H, H_e), 2.61 (t, $^3J_{HH}$ = 8.8 Hz, 2H, H_d), –0.69 (br, 6 H, $AlMe_2$).

^{13}C{^{1}H} NMR (75 MHz, C_6D_6, 298 K): δ [ppm] = 165.01 (d, $^4J_{CP}$ = 5.25 Hz, *ipso* carbon attached to C=N), 162.6 (d, $^2J_{CP}$ = 22.5 Hz, ind-*C*-NCH2), 140.52 (br, PhP-*C*-C_5H_5), 134.92 (d, $^2J_{CP}$ = 7.5 Hz, *C*-H_o), 134.12 (d, $^2J_{CP}$ = 9 Hz, *C*- H_a), 132.39 (d, $^3J_{CP}$ = 9 Hz, ind-*C*-CH_2CH_2), 131.15 (s, *C*- H_m/ H_p), 130.05 (s, *C*- H_m/ H_p), 129.90 (s, *C*-H_c), 129.56 (s, *C*-PPh_2), 129.23 (s, $C_{o/m/p-CPh2}$), 128.81 (s, $C_{o/m/p-CPh2}$) 128.75 (s, $C_{o/m/p-CPh2}$), 112.61 (d, $^1J_{CP}$ = 12.75 Hz, *C*-H_b), 89.84(s, N=CPh_2), 50.01 (s, *C*-H_e), 29.27 (s, *C*-H_d), –9.62 (br, $AlMe_2$).

^{31}P{^{1}H} NMR (121 MHz, C_6D_6, 298 K): δ [ppm] = 35.29

Elemental Analysis: $C_{35}H_{33}AlN_3P$ Calculated: C 75.93% H 6.01% N 7.59%

Found: C 75.02% H 5.90% N 6.86%

Due to instability of the activated azo substrate the values always deviated somewhat more than usually acceptable, especially for the N value.

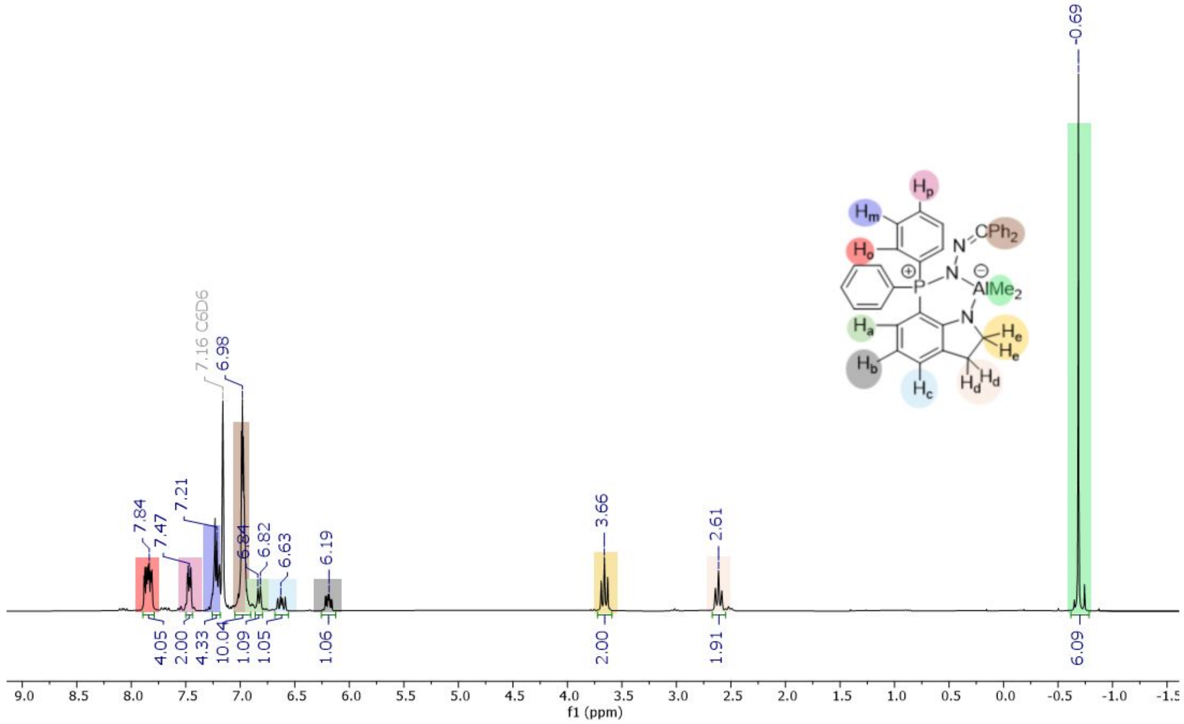

Figure S36 ^{1}H NMR spectrum of **3-CNN** (300 MHz, C_6D_6, 298 K).

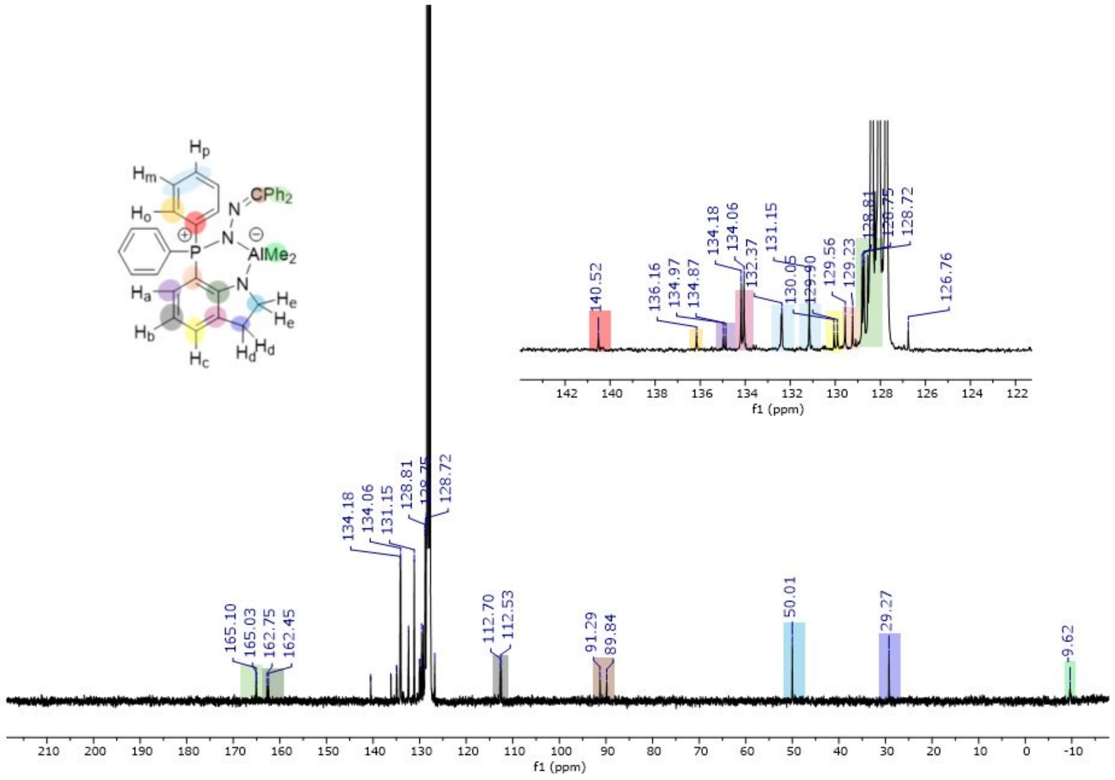

Figure S37 ^{13}C{^{1}H} NMR spectrum of **3-CNN** (300 MHz, C_6D_6, 298 K).

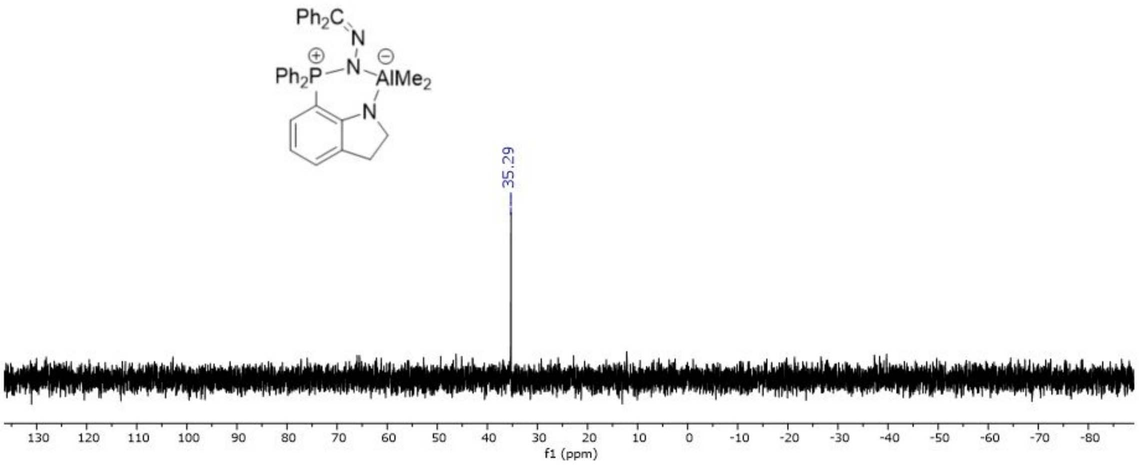

Figure S38 $^{31}P\{^{1}H\}$ NMR spectrum of **3-CNN** (121 MHz, C_6D_6, 298 K).

2.10 Synthesis of $Ph_2P(Ind)Al(C_6F_5)_2(Ph_2CN_2)$, 4-CNN

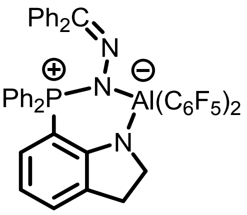

Compound **4** was generated in situ by mixing **$Ph_2P(Ind)H$** (50 mg, 0.15 mmol, 1.00 eq) and $MeAl(C_6F_5)_2$ (0.3 mL, 0.5 M in toluene, 0.15 mmol, 1.00 eq) in toluene (2 mL). After refluxing for 2 days a solution of diphenyldiazomethane (29 mg, 0.15 mmol, 1.00 eq) in 3 ml toluene was added slowly and the mixture was stirred for an additional hour at room temperature. After removal of the solvent the oily residue was extracted with hexane (2 mL) and stirred overnight. This caused precipitation of $Ph_2P(Ind)Al(C_6F_5)_2(Ph_2CN_2)$, **4-CNN** (68 mg, 0.08 mmol, 53%) as a colorless solid which was washed with hexane and dried under reduced pressure.

Crystals suitable for X-ray diffraction analysis were grown by layering a concentrated benzene solution with hexane.

Note: In contrast to the parent compound **4**, **4-CNN** does not appear to exhibit scrambling behavior, as the ^{1}H and ^{31}P NMR spectra display only one major set of signals. However, although the analyzed sample was recrystallized several times, the NMR spectra reproducibly showed the presence of minor impurities. In the elemental analysis, the measured carbon and hydrogen values only slightly exceeded the expected values, while the nitrogen value was significantly lower than anticipated. It was therefore concluded that, due to the strong activation of the azo substrate, **4-CNN** partially undergoes decomposition with concomitant release of N_2.

Surprisingly, the ^{19}F NMR spectrum displayed a second set of signals for the C_6F_5 group. The integrals of these signals appear too large to assign them to a minor decomposition product, as no corresponding signals of comparable intensity were observed in the ^{1}H or ^{31}P NMR spectra. It is therefore assumed that, due to the rapid data acquisition, the ^{19}F NMR spectrum resolves a dynamic process that is not captured by the other NMR experiments.

^{1}H NMR (400 MHz, C_6D_6, 298 K): δ [ppm] = 7.72 (m, 4H, *o*-PPh_2), 7.22 (m, 2H, *p*-PPh_2), 6.93 - 6.76 (m, 16 H, *m*-PPh_2/H_{Ar}), 6.29 (m, 1H, H_{Ar}), 3.46 (t, $^{3}J_{HH}$ = 8.9 Hz, 2H, H_e), 2.63 (t, $^{3}J_{HH}$ = 8.8 Hz, 2H, H_d).

^{31}P{^{1}H} NMR (162 MHz, C_6D_6, 298 K): δ [ppm] = 38.98 (s).

^{19}F NMR (376 MHz, C_6D_6, 298 K): δ [ppm] = –120.13 (m, 4 F, *o*-F), –155.64 (t, $^{3}J_{FF}$ = = 19.7 Hz, 2 F, *p*-F), –162.78 (m, 4 F, *m*-F).

Elemental Analysis: $C_{45}H_{27}AlF_{10}N_3P$ Calculated: C 63.02% H 3.17% N 4.90%

Found: C 63.68% H 3.75% N 3.18%

Due to instability of the activated azo substrate the values always deviated somewhat more than usually acceptable, especially for the N value.

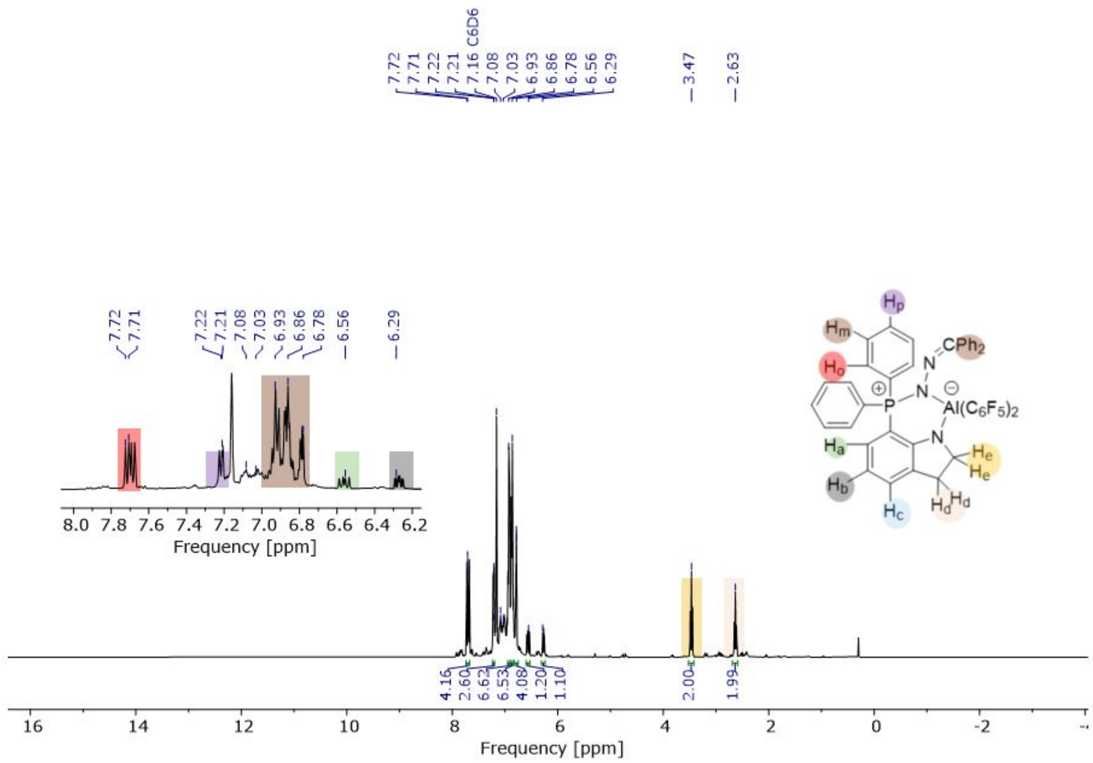

Figure S39 ^{1}H NMR spectrum of **4-CNN** (400 MHz, C_6D_6, 298 K).

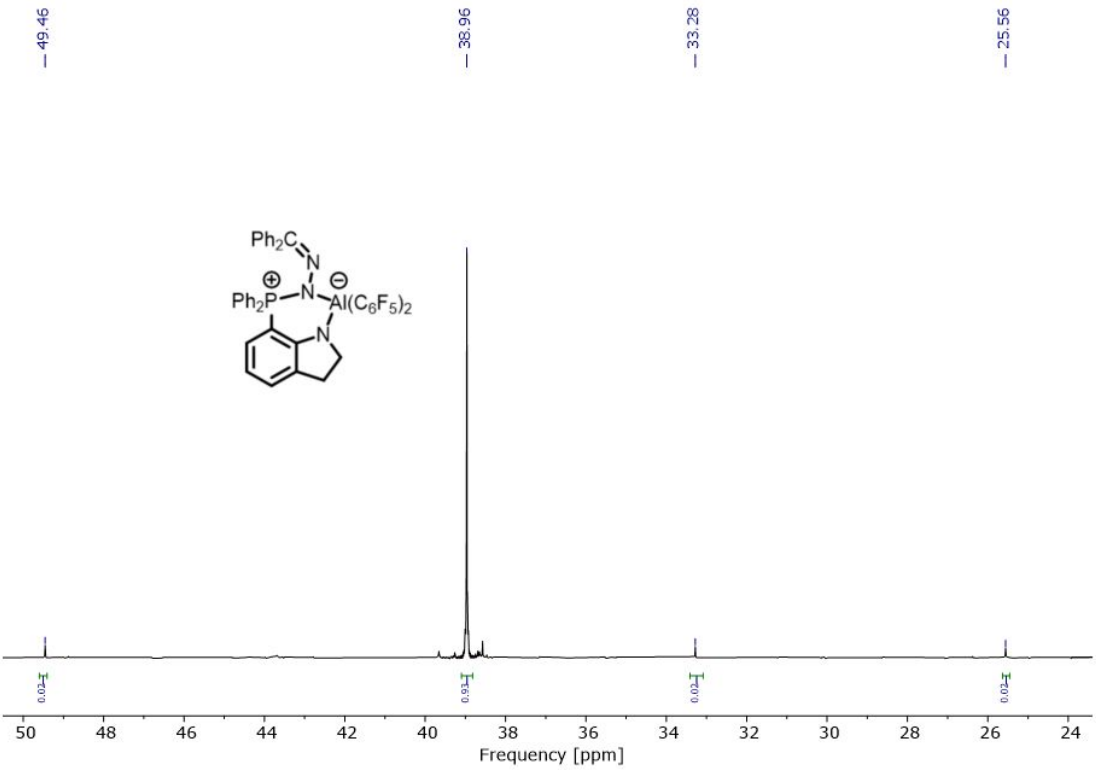

Figure S40 $^{31}P\{^{1}H\}$ NMR spectrum of **4-CNN** (162 MHz, C_6D_6, 298 K).

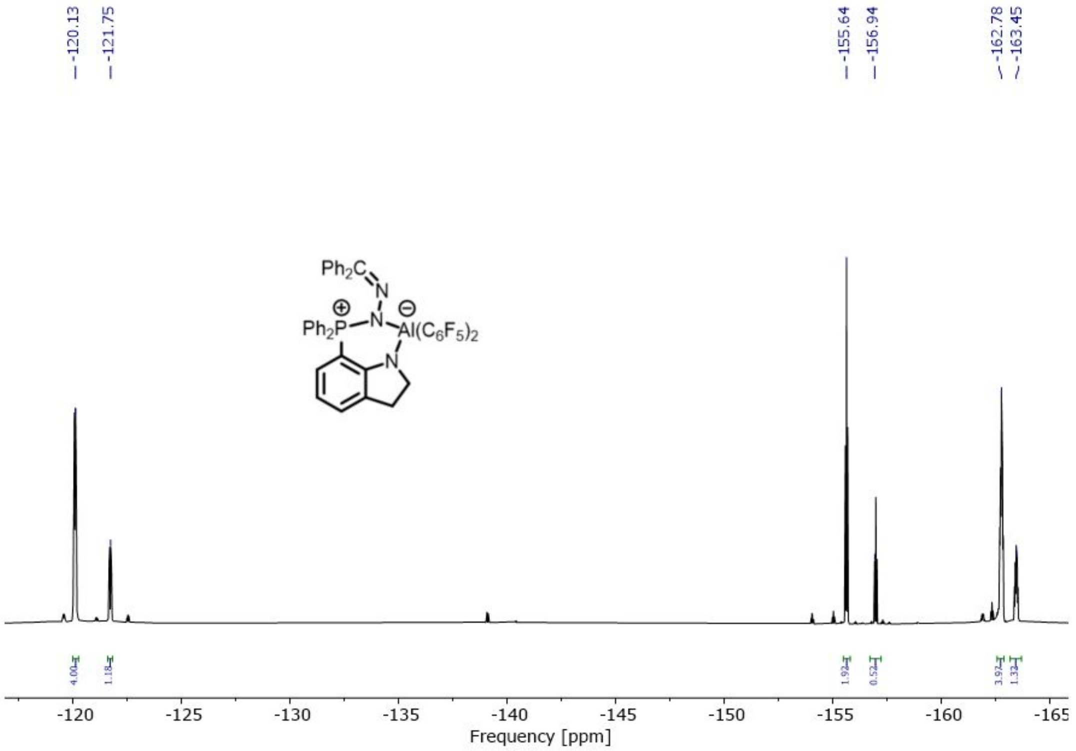

Figure S41 ^{19}**F NMR** spectrum of **4-CNN** (376 MHz, C_6D_6, 298 K).

2.11. Synthesis of $Ph_2P(Ind\text{-}CO_2)Al(Me)_2$, 3-$CO_2$

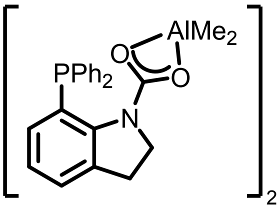

A benzene solution (1 mL) of compound **3** (60 mg, 0.17mmol) was subjected to three cycles of freeze-pump-thaw degassing. CO_2 was added (approx. 2 bar) and the mixture was stirred for 2 h causing a complete discoloration of the solution. After removal of the solvent the oily residue was taken up in hexane (1 mL) and stirred overnight. This caused precipitation of $[Ph_2P(Ind\text{-}CO_2)Al(Me)_2$, **3-$CO_2$** (44 mg, 0.11 mmol, 65%) as a colorless solid which was washed with hexane and dried under reduced pressure.

Crystals suitable for X-ray diffraction analysis were grown from a concentrated benzene solution.

^{1}H NMR (500 MHz, C_6D_6, 298 K): δ [ppm] = 7.49 (m, 4H, *o*-PPh_2), 7.08-7.02 (m, 6H, *m*-PPh_2 and *p*-PPh_2), 6.97 (m, 1H, ^{Ar}H), 6.70 -6.66 (m, 2H, ^{Ar}H), 3.61 (t, $^3J_{HH}$ = 7.4 Hz, 2H, CH_2), 2.18 (br, 2H, CH_2), –0.20 (br, 6 H, $AlMe_2$).

^{13}C{^{1}H} NMR (125 MHz, C_6D_6, 298 K): δ [ppm] = 155.84 (br, ind-*C*-NCH2), 143.3 (d, $^1J_{CP}$ = 18.75 Hz, PhP-*C*-C_5H_5), 137.52 (d, $^2J_{CP}$ = 18.75 Hz, *C*-H_o), 134.79 (d, $^2J_{CP}$ = 28.75 Hz, *C*-H_a), 132.62 (d, $^3J_{CP}$ = 6.25 Hz, ind-*C*-CH_2CH_2), 130.60 (s, *C*- H_m/ H_p), 129.35 (s, *C*- H_m/ H_p), 128.77 (d, $^1J_{CP}$ = 8.75 Hz, *C*-PPh_2), 125.84 (s, *C*-H_c), 124.45 (s, *C*-H_b), 51.25 (*C*-H_e), 28.82 (*C*-H_d), –8.73 (br, Al*Me_2*).

^{31}P{^{1}H} NMR (202 MHz, C_6D_6, 298 K): δ [ppm] = –7.56 (s)

Elemental Analysis: $[C_{23}H_{24}AlNO_2P]_2$ Calculated: C 68.31% H 5.98% N 3.46%

Found: C 68.77% H 5.83% N 3.41%

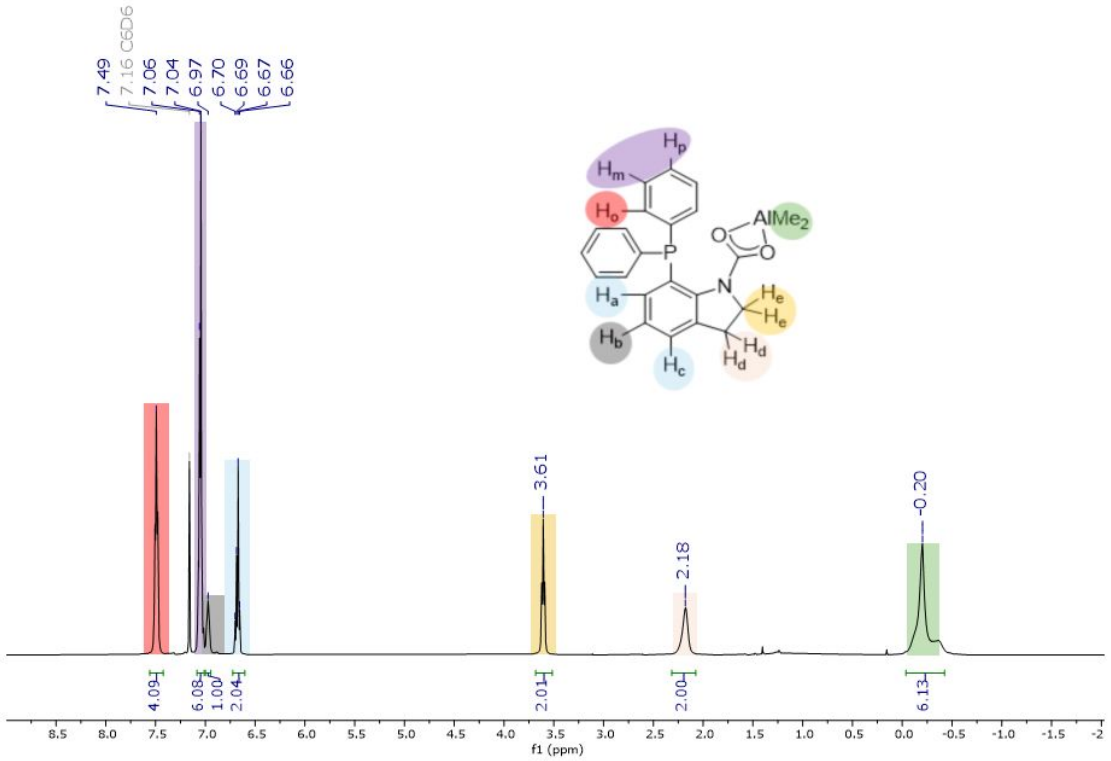

Figure S42. ^{1}H NMR spectrum of **3-CO$_2$** (500 MHz, C_6D_6, 298 K).

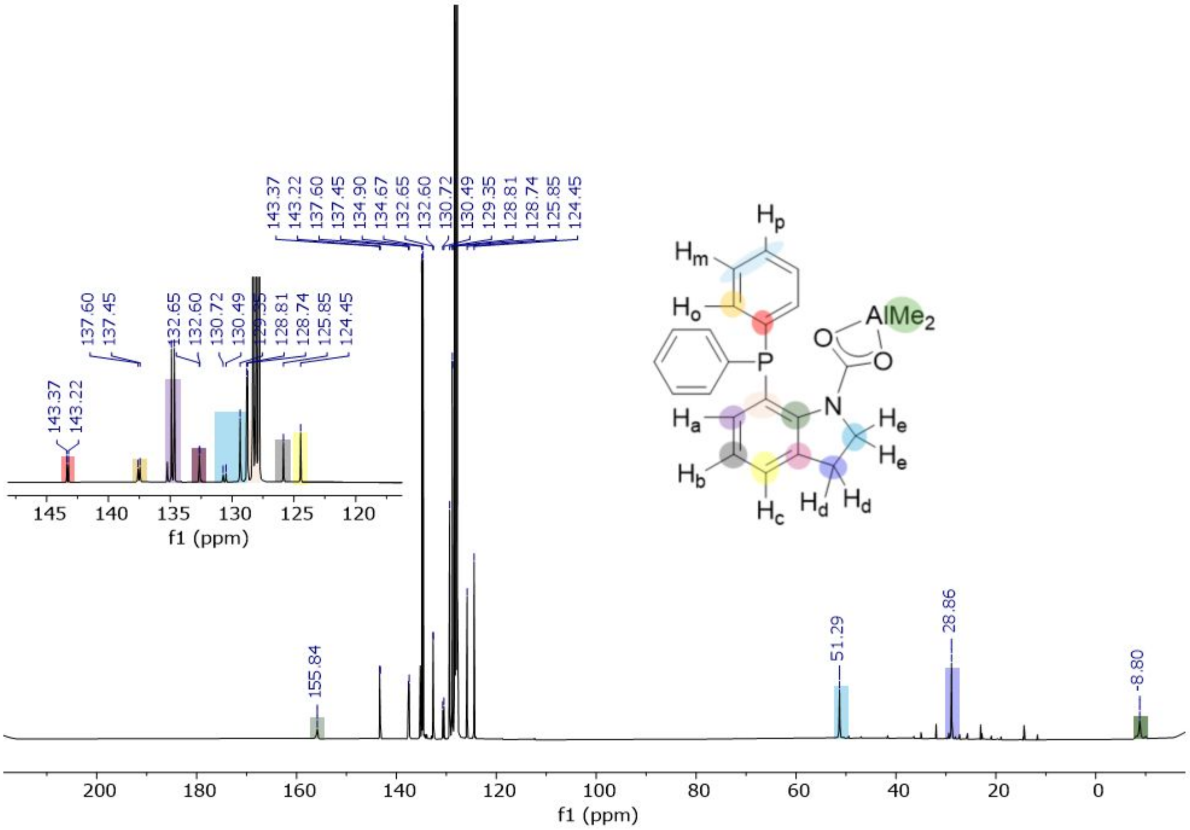

Figure S43. $^{13}C\{^{1}H\}$ NMR spectrum of **3-CO$_2$** (125 MHz, C_6D_6, 298 K).

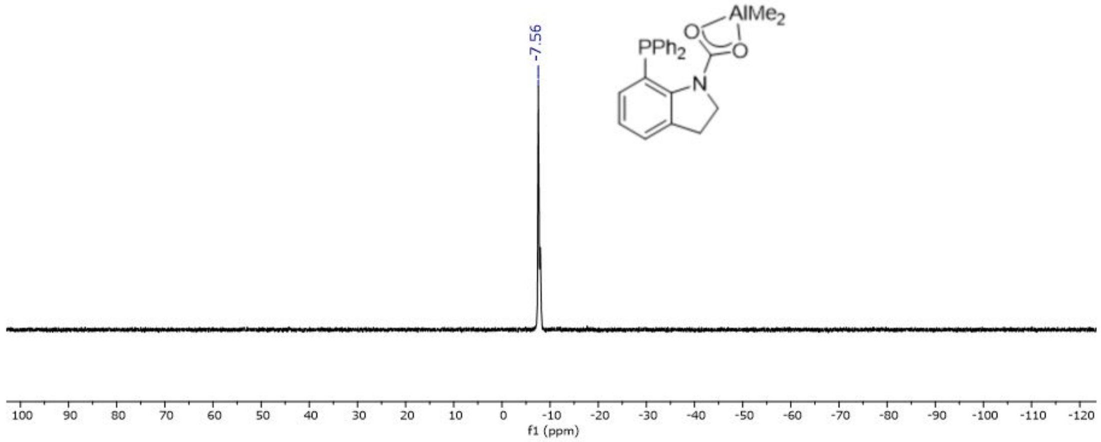

Figure S44. $^{31}P\{^{1}H\}$ NMR spectrum **3-CO₂** (202 MHz, C_6D_6, 298 K).

2.12. Synthesis of $Ph_2P(Ind)Al(C_6F_5)_2CO_2$, 4-CO₂

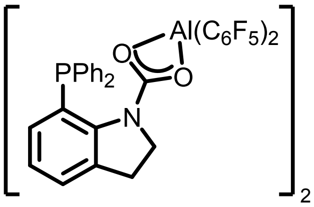

Compound **4** was generated in situ by mixing **$Ph_2P(Ind)H$** (50 mg, 0.15 mmol) and $MeAl(C_6F_5)_2$ (0.3 mL, 0.5 M in toluene, 0.15 mmol) in toluene (2 mL). After refluxing for 2 days the mixture was subjected to three cycles of freeze-pump-thaw degassing. CO_2 was added (approx. 2 bar) and the mixture was stirred for 2 h causing a complete discoloration of the solution. After removal of the solvent the oily residue dissolved in hexane (2 mL) and stirred overnight. This caused precipitation of $[Ph_2P(Ind\text{-}CO_2)Al(C_6F_5)_2]_2$, **4-$CO_2$** (72 mg, 0.10 mmol, 68%) as a colorless solid which was washed with hexane and dried under reduced pressure.

Crystals suitable for X-ray diffraction analysis were grown by slow evaporation of a benzene/hexane mixture.

Note: Elemental analysis confirmed that the isolated material features the expected composition for $[Ph_2P(Ind\text{-}CO_2)Al(C_6F_5)_2]_2$, indicating purity of the sample. However, analysis by ^{1}H and ^{31}P NMR spectroscopy revealed the presence of two species in solution (see Figures S45 and S46), even after multiple recrystallization cycles. This observation suggests that **4-CO_2** undergoes scrambling behavior similar to that observed for its parent compound **4**. The major species in solution was therefore assigned to compound **4-CO_2**, whereas the minor species was tentatively assigned to $[Ph_2P(Ind)]_2Al(C_6F_5)$, **4'-CO_2**.

The ^{19}F NMR spectrum displayed several minor sets of signals corresponding to scrambling products. However, due to their similar intensities, none of the signals could be confidently assigned to **4'-CO_2.**

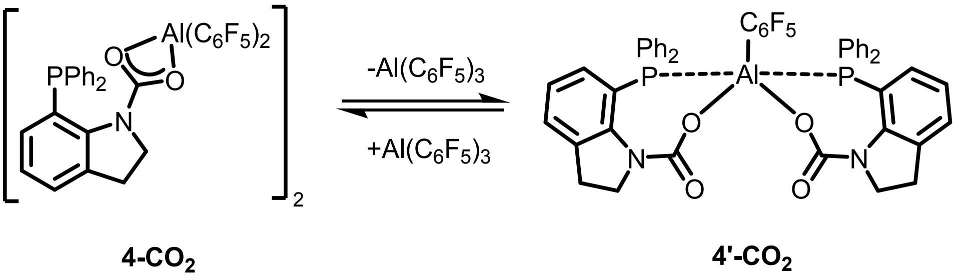

Scheme S6: Proposed ligand scrambling of **4-CO_2**.

Elemental Analysis: $[C_{33}H_{18}AlF_{10}NO_2P]_2$ Calculated: C 55.95% H 2.56% N 1.98%

Found: C 55.21% H 2.38% N 1.99%

Spectroscopic data for $[Ph_2P(Ind\text{-}CO_2)Al(C_6F_5)_2]_2$, **4-CO₂**

^{1}H NMR (400 MHz, C_6D_6, 298 K): δ [ppm] = 7.30 – 6.50 (overlapping multiplets of **4-CO₂** and **4'-CO₂**, 13 H, PPh_2/H_{Ar}), 3.81 (t, $^3J_{HH}$ = 7.4 Hz, 2 H, CH_2), 2.39 (t, $^3J_{HH}$ = 7.4 Hz, 2 H, CH_2).

^{31}P{^{1}H} NMR (162 MHz, CD_2Cl_2, 298 K): δ [ppm] = –6.42 (q).

^{19}F{^{1}H} NMR (376 MHz, CD_2Cl_2, 298 K): δ [ppm] = –123.18 (m, 4 F, *o*-C_6F_5), 153.40 (t, J = 19.6 Hz, 1F, *o*-C_6F_5), –161.5 (m, 4F, *p*-C_6F_5).

Spectroscopic data for $[Ph_2P(Ind\text{-}CO_2)]_2Al(C_6F_5)$, **4'-CO₂**

^{1}H NMR (400 MHz, C_6D_6, 298 K): δ [ppm] = 7.30 – 6.50 (overlapping multiplets of **4-CO₂** and **4'-CO₂**, 26 H, PPh_2/H_{Ar}), 3.92 (t, $^3J_{HH}$ = 7.4 Hz, 4 H, CH_2), 2.47 (t, $^3J_{HH}$ = 7.4 Hz, 4 H, CH_2)

^{31}P{^{1}H} NMR (162 MHz, CD_2Cl_2, 298 K): δ [ppm] = –7.44 (q).

Due to the presence of more than one species in solution, recording ^{13}C NMR data was not pursued. Because the ^{1}H NMR signals of **4-CO₂** and **4'-CO₂** overlap in the 6–8 ppm region, proper assignment of the aromatic signals was not possible.

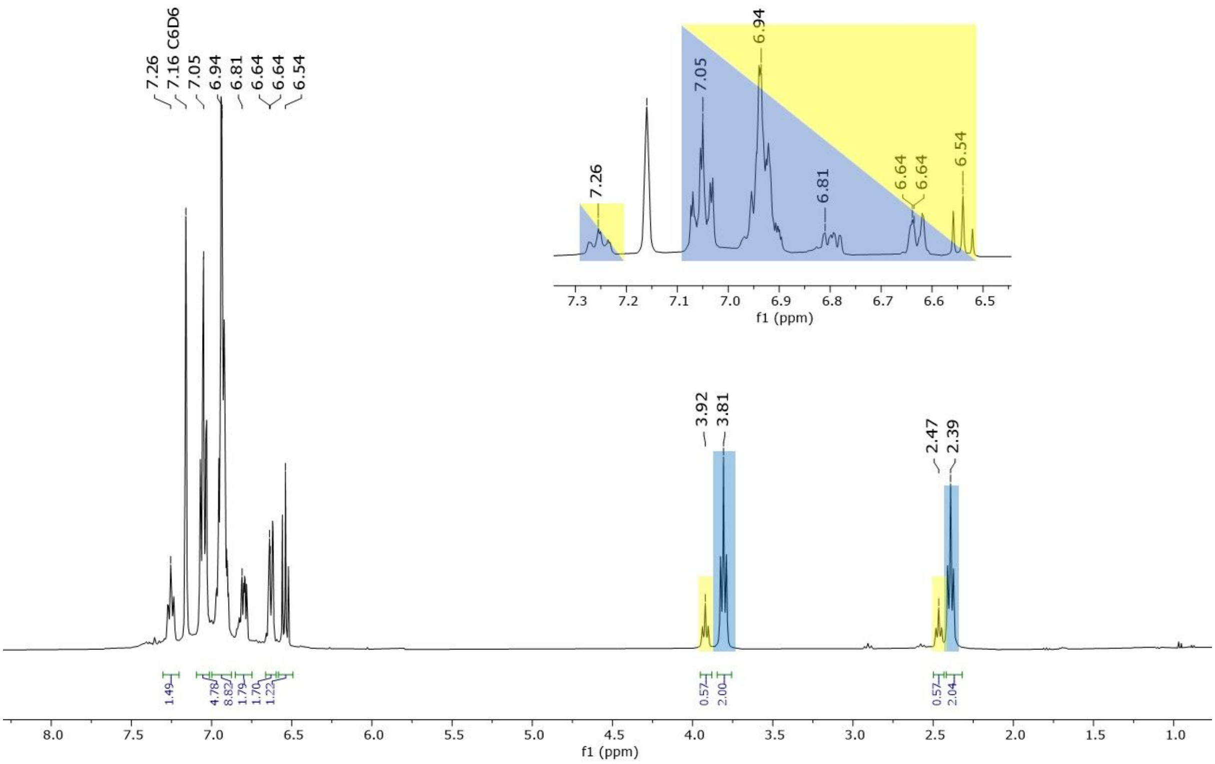

Figure S45 ^{1}H NMR spectrum of **4-CO₂** (400 MHz, C_6D_6, 298 K, blue labels = **4-CO₂**, yellow label = **4'-CO₂**).

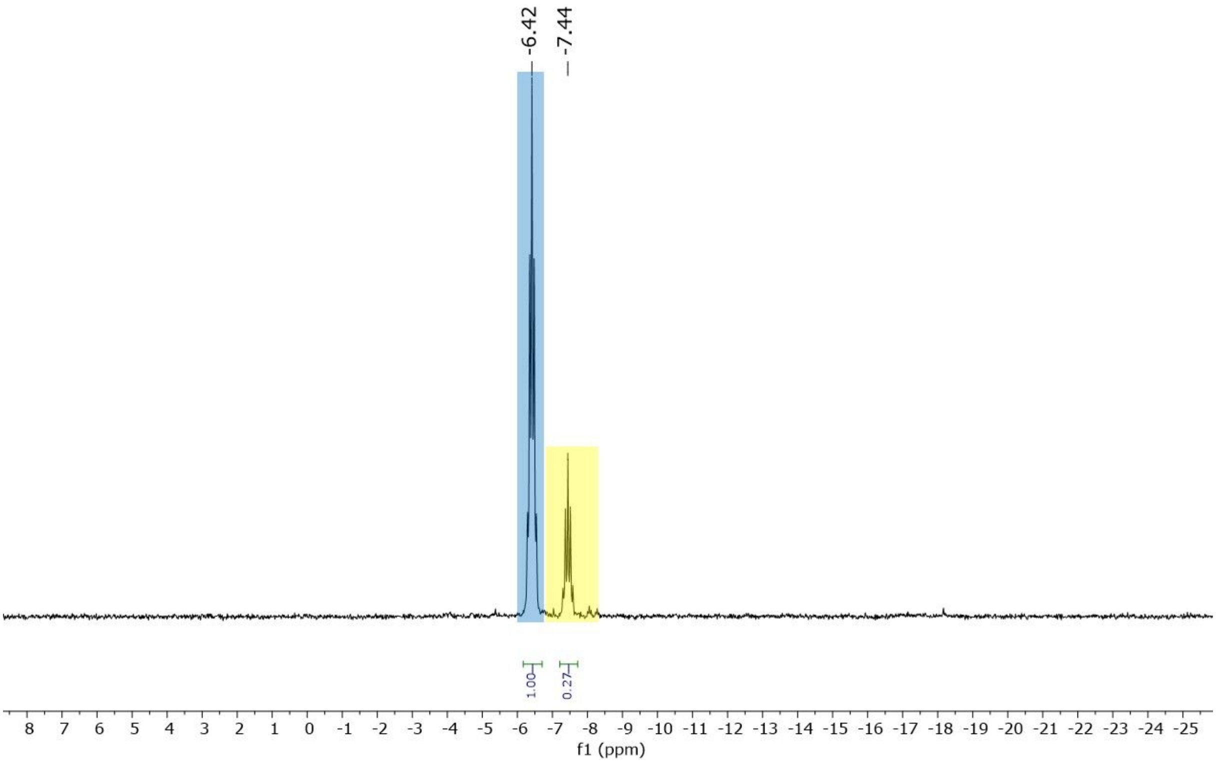

Figure S46 $^{31}P\{^1H\}$ NMR spectrum of **4-CO₂** (162 MHz, C_6D_6, 298 K, blue label = **4-CO₂**, yellow label = **4’-CO₂**).

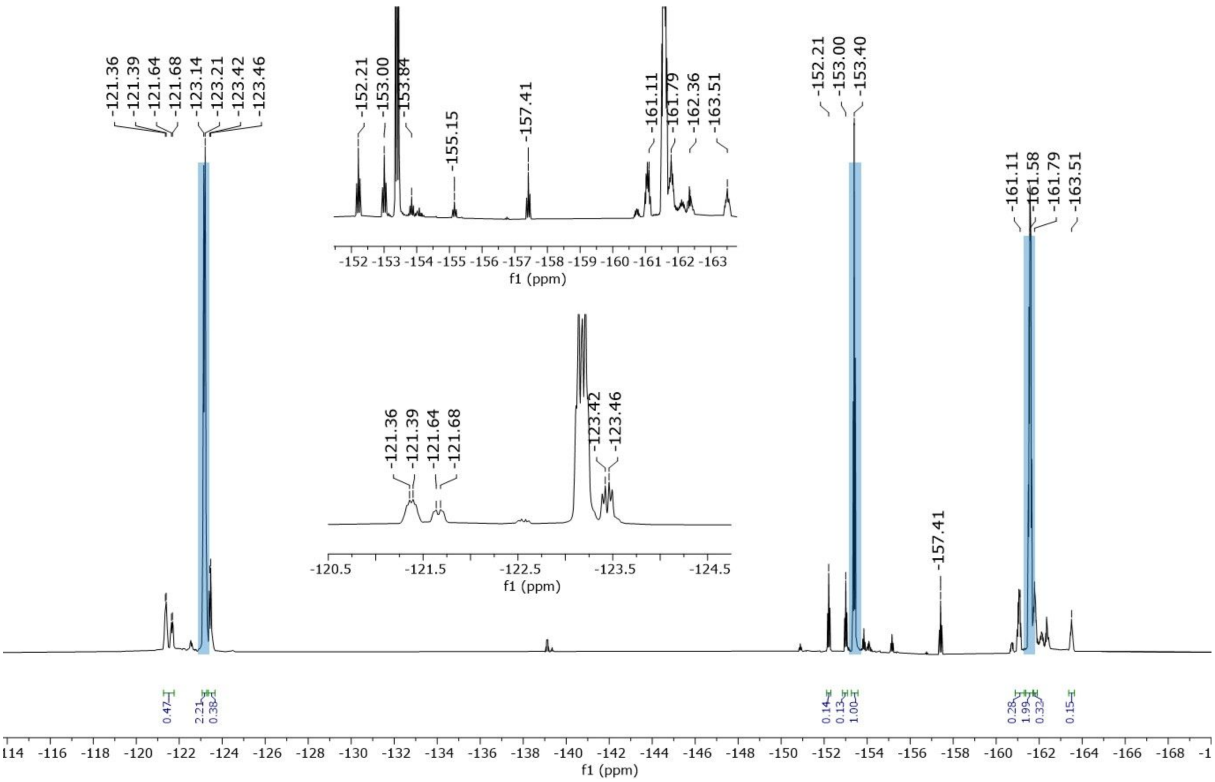

Figure S47 ^{19}F NMR spectrum of **4-CO₂** (376 MHz, C_6D_6, 298 K, blue label = **4-CO₂**).

3. Fluorescence of 3

Compound **3** exhibited fluorescence both in solution and in the solid state upon UV irradiation (Figure S48). Therefore, **3** was further characterized by UV/vis spectroscopy using an Agilent 8453 UV–Vis spectrophotometer and SUPRASIL quartz cuvettes (Hellma Analytics) with a path length of 10 mm. Comparison of the UV/vis spectra of **3** and the parent ligand $Ph_2P(Ind)H$ revealed that alumination of the indoline backbone gives rise to an intense absorption band at 403 nm, which is likely responsible for the fluorescent properties of the compound.

Compound **4** likewise displayed fluorescence in both solution and the solid state. However, due to the presence of two species in solution arising from the proposed ligand scrambling process, further UV/vis spectroscopic investigations were not pursued.

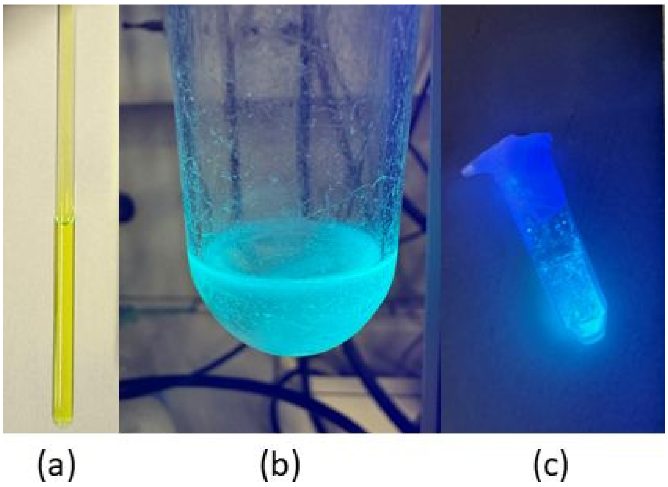

Figure S48. a) Photograph in day light (solution in C_6D_6); b) Photograph with UV-excitation (solution in toluene); c) Photograph with UV-excitation in solid state.

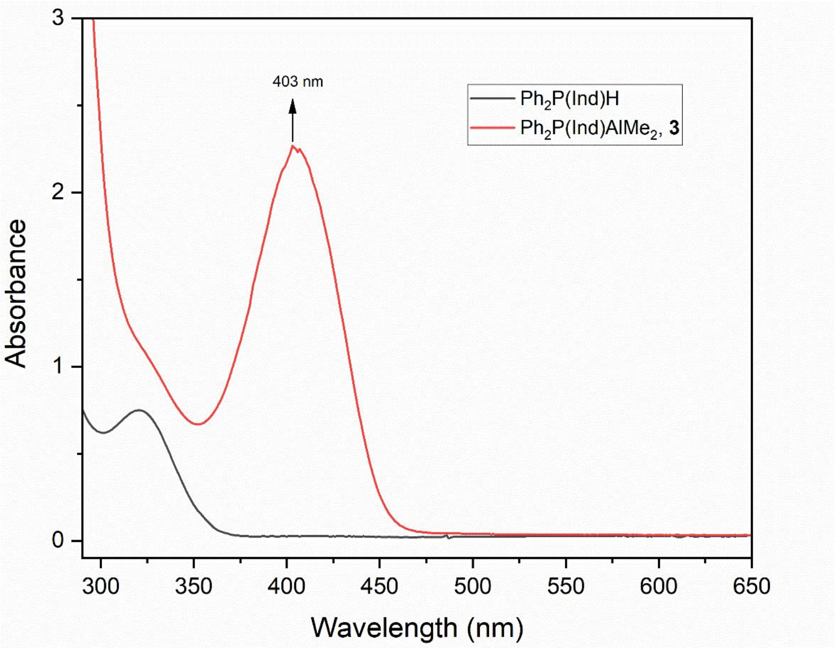

Figure S49. Superimposed UV-vis spectra of $Ph_2P(Ind)H$ (black) and compound **3** (red) in DCM (0.5 mM).

4. Acceptor Number by Gutmann-Beckett

For determination of the acceptor number (AN), the conventional Gutmann–Beckett method (GBM) was applied.[7] The corresponding indoline alane (0.04 mmol, 1 eq) compounds were dissolved in DCM-d_2 and a capillary filled with 10% triphenylphosphine in C_6D_6 was added as a reference (δ = –5.2 ppm). Triethylphosphine oxide (5.36 mg, 0.04 mmol, 1.00 eq) in DCM-d_2 was added and a $^{31}P\{^1H\}$ NMR spectrum was recorded. From the chemical shift of the Et_3PO adduct the acceptor numbers were calculated using the formula $AN = 2.21 \times (\delta(^{31}P_{Et3PO}) - 41)$.

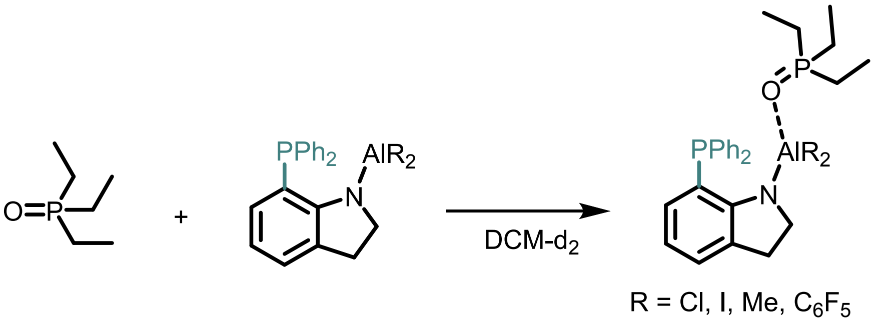

Scheme S6: Adduct formation of indoline aluminum compounds with Et_3PO.

Although some of the compounds **1**, **2** and **4** exhibit scrambling behavior or feature a monomer-dimer equilibrium, leading to the presence of multiple species in solution, the addition of Et_3PO resulted in NMR spectra displaying only a single set of signals. This indicates that upon adduct formation all species converted into the desired (Ind)$AlR_2(Et_3PO)$ product.

Notably, the AN of (Ind)$AlMe_2$ was found to be slightly higher than that of compound **3**, which may be attributed to the absence of electron donation from the phosphine center to the aluminum center.

Table S1. Collected $\delta(^{31}P_{Et3PO})$ values for Et_3PO adducts and the calculated ANs of the parent aluminum compounds.

Compound	$\delta(^{31}P_{Et3PO})$ [ppm]	AN
**(Ind)$AlMe_2$**	69.23	62
**Ph_2P(Ind)$AlCl_2$, 1**	73.49	71
**Ph_2P(Ind)AlI_2, 2**	84.06	95
**Ph_2P(Ind)$AlMe_2$, 3**	67.22	57
**Ph_2P(Ind)$Al(C_6F_5)_2$, 4**[(a)]	68.43	60

(a) For compound 4 the AN was determined in C_6D_6.

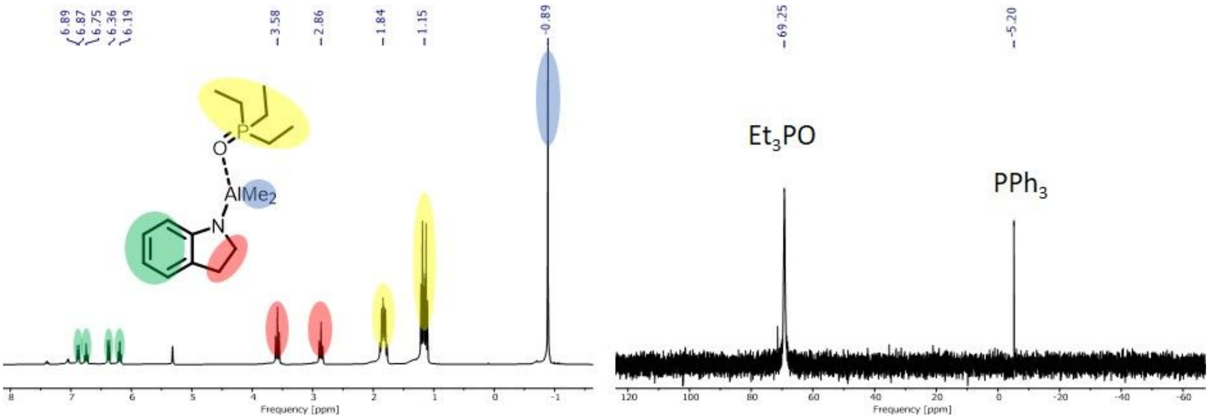

Figure S50. ^{1}H (left) and ^{31}P (right) NMR spectra of **IndAlMe$_2$(Et$_3$PO)** (300/121 MHz, DCM-d$_2$, 298 K).

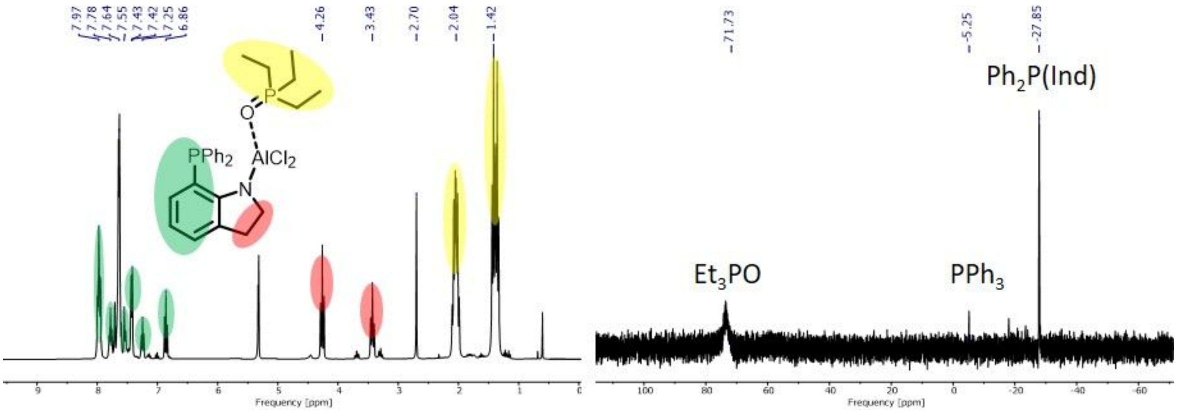

Figure S51. ^{1}H (left) and ^{31}P (right) NMR spectra of **1-Et$_3$PO** (300/121 MHz, DCM-d$_2$, 298 K).

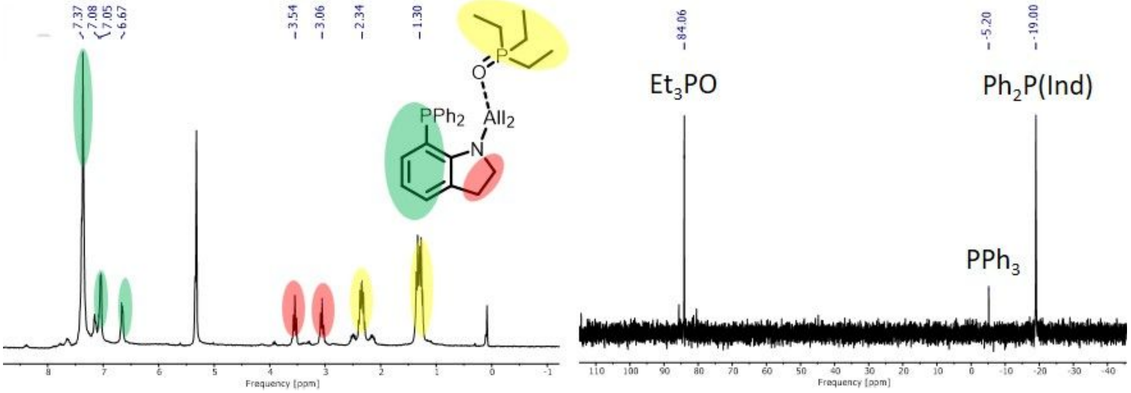

Figure S52. ^{1}H (left) and ^{31}P (right) NMR spectra of **2-Et$_3$PO** (300/121 MHz, DCM-d$_2$, 298 K).

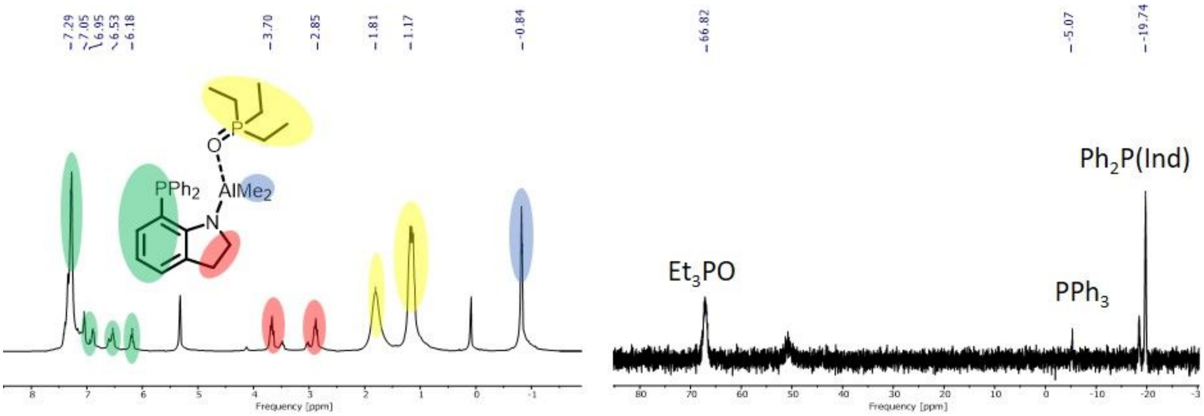

Figure S53. ^{1}H (left) and ^{31}P (right) NMR spectra of **3-Et₃PO** (300/121 MHz, DCM-d_2, 298 K).

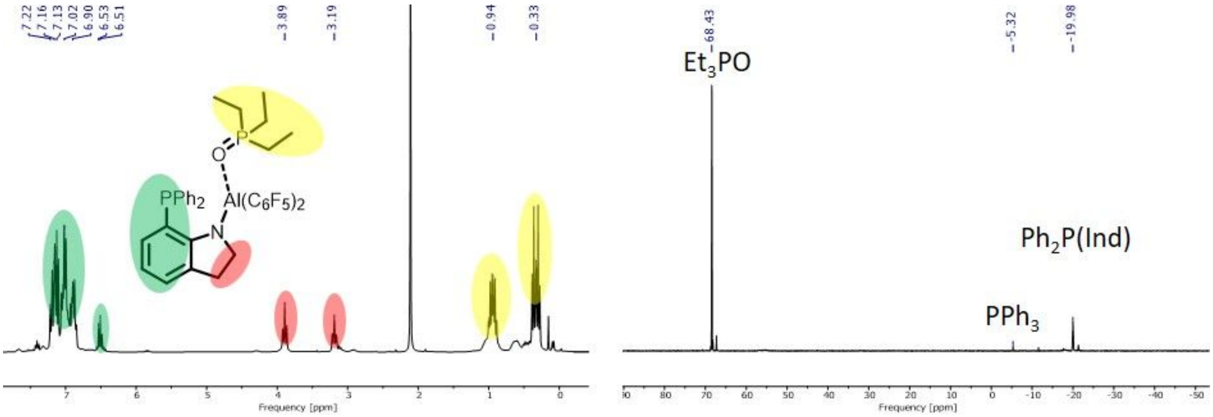

Figure S54. ^{1}H (left) and ^{31}P (right) NMR spectra of **4-Et₃PO** (300/121 MHz, C_6D_6, 298 K).

5. Dehydrodefluorination (DHF) reaction studies

General procedure

A J. Young NMR tube was charged with the corresponding aluminum reagent (0.05 mmol, 1 equiv), fluorocyclohexane (5.50 µL, 0.05 mmol, 1 equiv), and C_6D_6 (0.6 mL). The mixture was shaken for 1 min. The reaction course was monitored by ^{1}H, ^{19}F, and ^{31}P NMR spectroscopy. After complete consumption of fluorocyclohexane, the mixture was passed through a pipette filled with silica and subsequently analyzed by GC–MS.

Recently, Crimmin et al. reported the DHF of activated hydrofluorocarbons mediated by simple potassium bases such as KHMDS.[8] Accordingly, control reaction using KHMDS or $C_6H_5CH_2K$ (BzK) were conducted under the same conditions as those employed for the aluminum compounds. In both cases, no reaction with fluorocyclohexane was observed, even after extended reaction times.

Table S2: Reactivity of Al compounds towards fluorcyclohexane.

**Reagent**	**AN**[(a)]	**Reactivity toward** 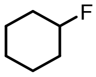
**3**	57	DHF, full conversion in 6 h
(Ind)$AlMe_2$	62	DHF
**4**	60	DHF, full conversion in <30min
Al(O^tBu)$_3$	21	No reaction
Al(NMe_2)$_3$	56[(b)]	No reaction
$AlMe_3$	59[(b)]	DHF, full conversion in 3 h
KHMDS	-	No reaction
BzK	-	No reaction

(a) Unless otherwise noted ANs has been determined by the Gutmann-Beckett method using DCM-d_2 as solvent (see section 3).
(b) Due to instability in chlorinated solvents the AN of these compounds was determined in C_6D_6.

5.1. DHF mediated by 3

Full consumption of fluorocyclohexane was observed 6 h after addition of compound **3**. The ^{1}H and ^{19}F NMR spectra indicated complete conversion of the substrate to cyclohexene, which was further confirmed by GC–MS analysis. Additionally, ^{1}H and ^{31}P NMR spectra revealed that, upon DHF of the substrate, **Ph_2P(Ind)H** was formed, indicating that the abstracted proton is bound as protonated indoline. Integration of the signals shows that, after complete conversion of one equivalent of fluorocyclohexane, approximately 50% of compound **3** remains unreacted. Furthermore, the characteristic signal for methane (~0.15 ppm) could be detected, meaning that Al-Me units are also involved in the proton abstraction. The ^{19}F NMR

spectrum displayed very broad signals in the region of −140 to −150 ppm, characteristic of aluminum-bound fluoride species of the type $AlMe_{3-x}F_x$.[9]

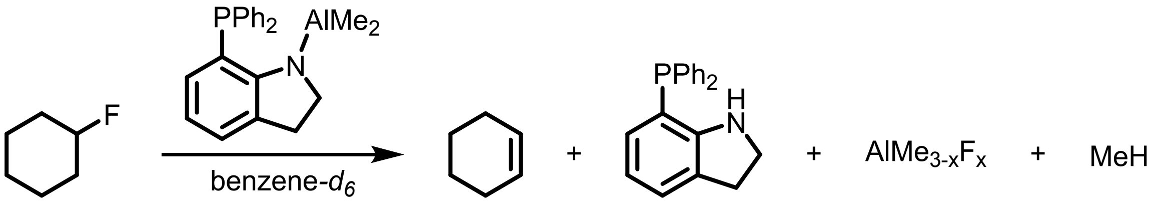

Scheme S6: DHF of fluorocyclohexane mediated by **3**.

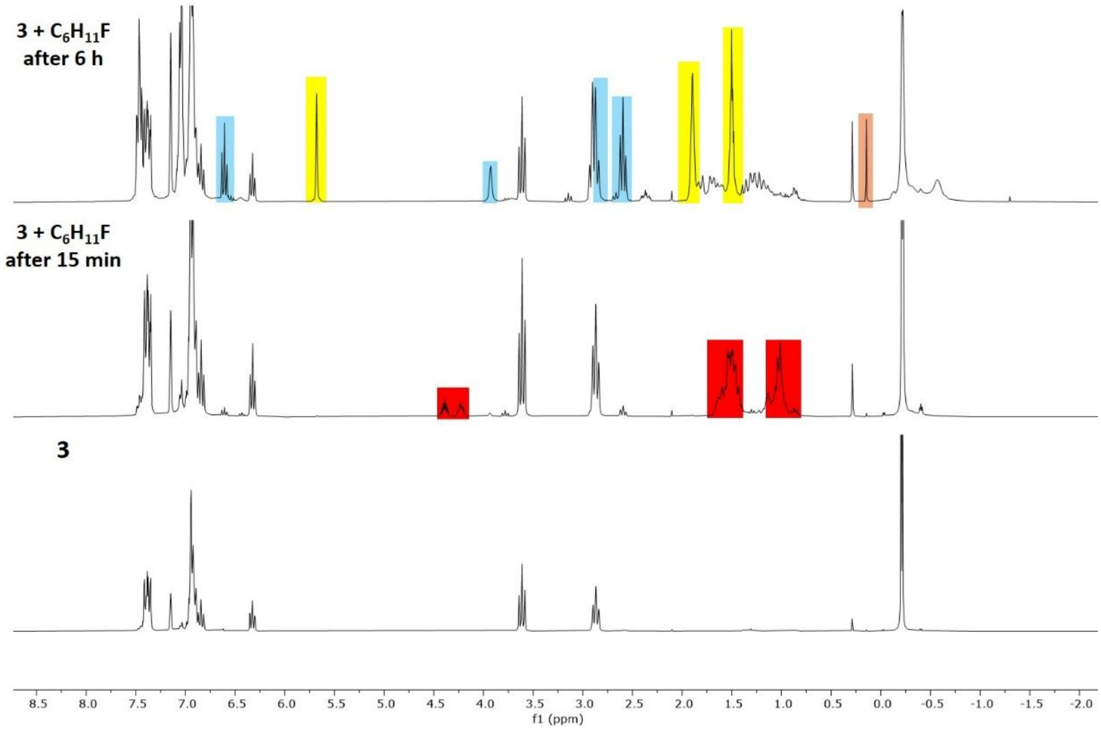

Figure S55. ^{1}H NMR stack plot (300 MHz, C_6D_6, 298 K) for the DHF of fluorocyclohexane mediated by **3** (red label = fluorocyclohexane, yellow label = cyclohexene, blue label = **$Ph_2P(Ind)H$**, green label = CH_4).

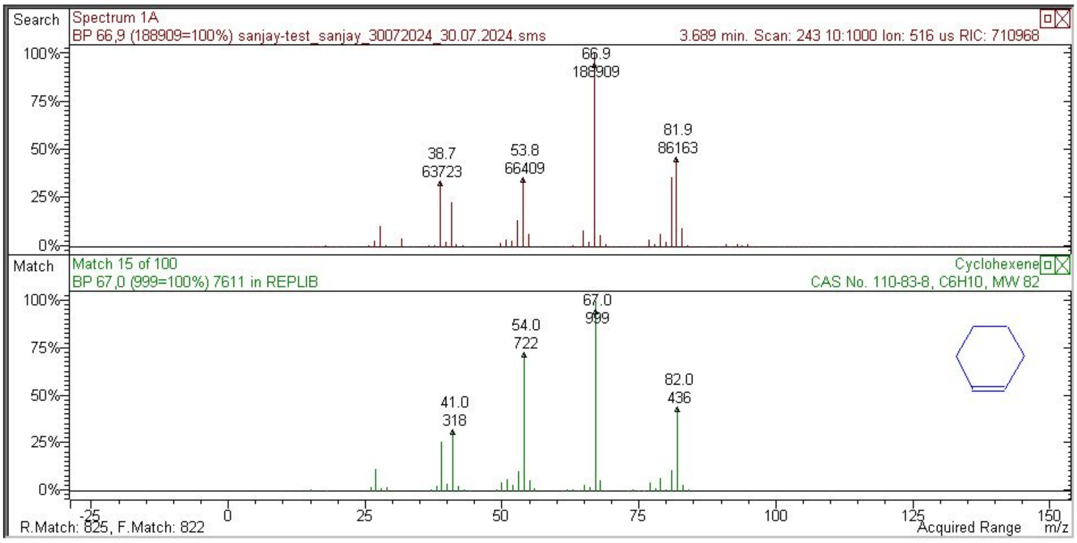

Figure S24. GC-MS spectrum of DHF of fluorocyclohexane mediated by **3**.

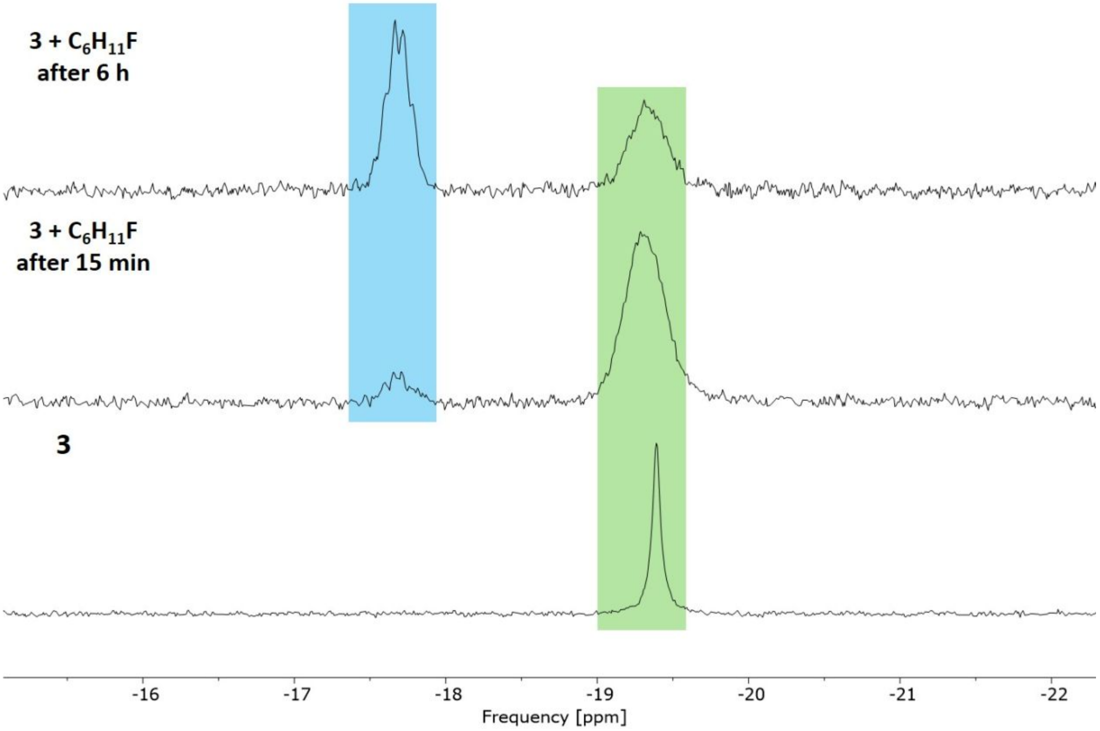

Figure S56. ^{31}P NMR stack plot (121 MHz, C_6D_6, 298 K) for the DHF of fluorocyclohexane mediated by **3** (blue label = **$Ph_2P(Ind)H$**, green label = **3**).

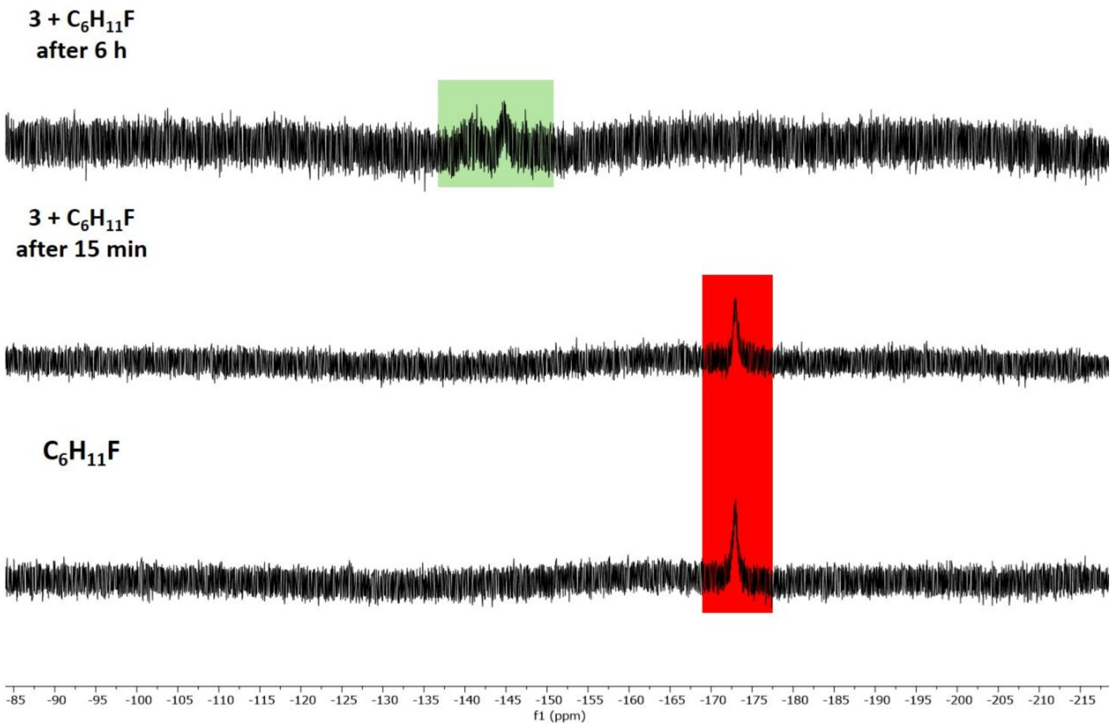

Figure S57. ^{19}F NMR stack plot (282 MHz, C_6D_6, 298 K) for the DHF of fluorocyclohexane mediated by **3** (red label = fluorocyclohexane, green label = $AlMe_{3-x}F_x$).

5.2. DHF mediated by 4

Full consumption of fluorocyclohexane was observed within 30 min after addition of compound **4**. The ^{1}H and ^{19}F NMR spectra indicated complete conversion of the substrate to cyclohexene. Additionally, ^{1}H and ^{31}P NMR spectra revealed that, upon DHF of the substrate, **$Ph_2P(Ind)H$** was formed. In contrast to the experiment with compound **3**, signals corresponding to **4** and **4'** were not detectable after complete conversion of one equivalent of fluorocyclohexane. Signals corresponding to C_6F_5H were observed only in trace amounts. These observations suggest that the low basicity of the C_6F_5 groups favors proton abstraction mainly by the aluminum amide functionality, whereas in the case of **3** the Al-Me units are sufficiently basic to participate in the DHF reaction. The ^{19}F NMR spectrum displayed signals in the region of −140 to −160 ppm, characteristic of aluminum-bound fluoride species of the type $Al(C_6F_5)_{3-x}F_x$.[9]

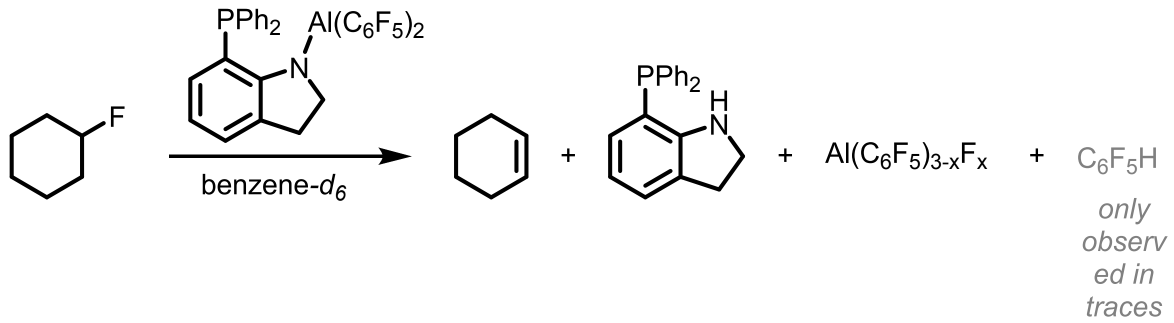

Scheme S7: DHF of fluorocyclohexane mediated by **4**.

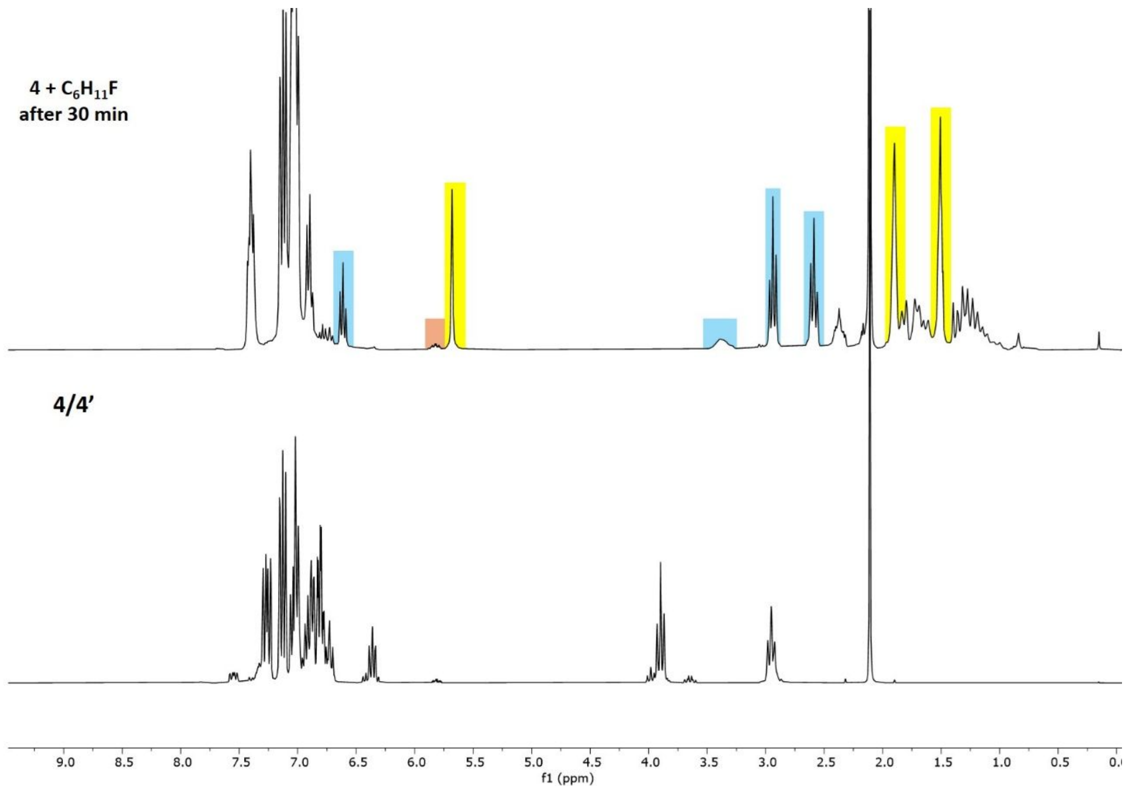

Figure S58. ^{1}H NMR stack plot (300 MHz, C_6D_6, 298 K) for the DHF of fluorocyclohexane mediated by **4** (yellow label = cyclohexene, blue label = **$Ph_2P(Ind)H$**, green label = C_6F_5H).

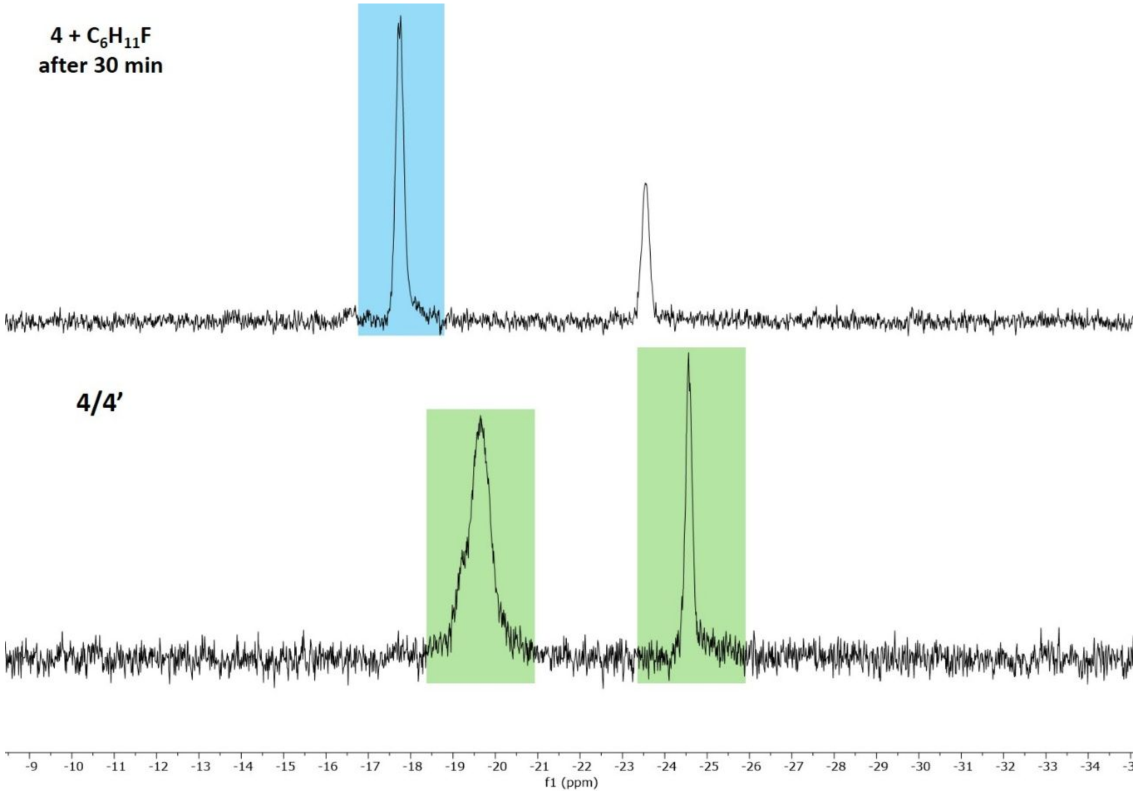

Figure S59. ^{31}P NMR stack plot (121 MHz, C_6D_6, 298 K) for the DHF of fluorocyclohexane mediated by **4** (blue label = **$Ph_2P(Ind)H$**, green label = **4/4’**).

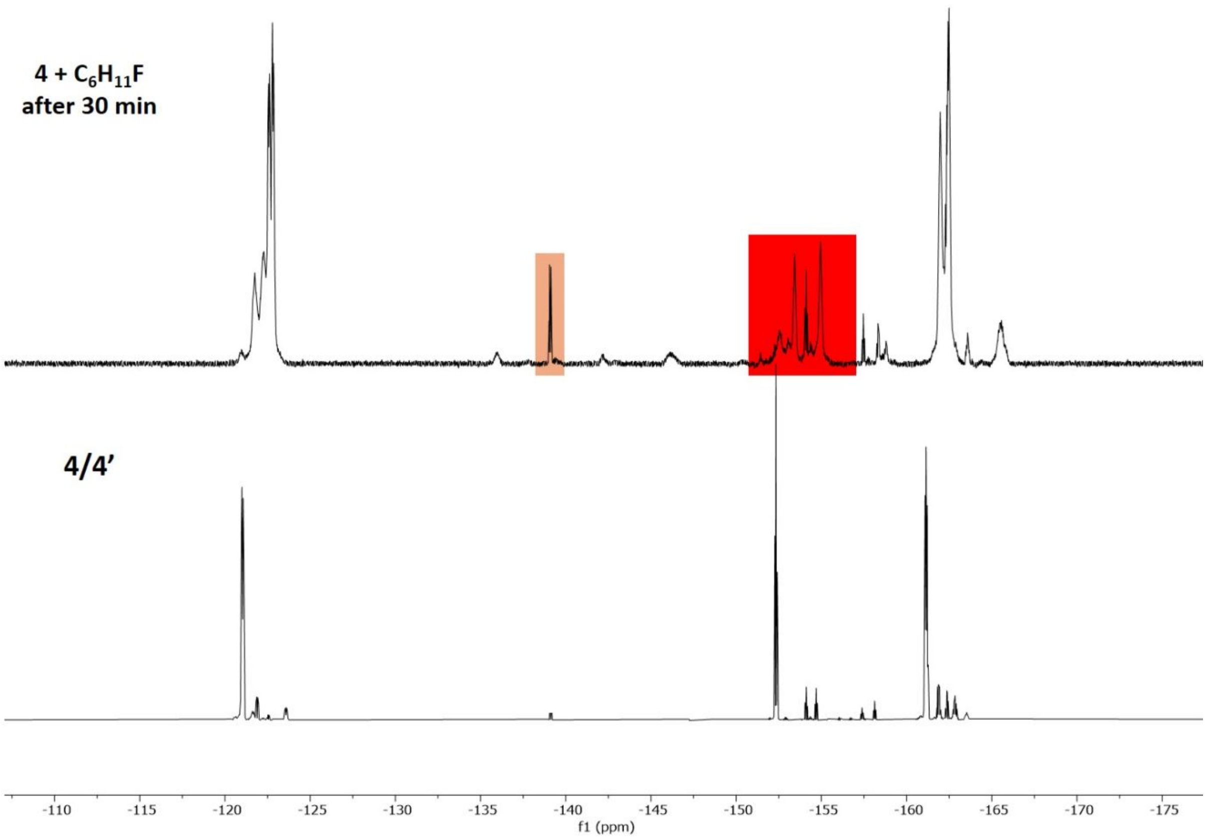

Figure S60. ^{19}F NMR stack plot (282 MHz, C_6D_6, 298 K) for the DHF of fluorocyclohexane mediated by **3** (green label = C_6F_5H, red label = $Al(C_6F_5)_{3-x}$**F_x**).

5.3. DHF mediated by (Ind)AlMe₂

After 24 h following the addition of $AlMe_3$, the formation of cyclohexene, (Ind)H, and methane was observed by ^1H NMR spectroscopy, indicating that the non-phosphinated indoline alane is also capable of mediating DHF. However, complete conversion was not achieved at this stage. The reaction was therefore accelerated by heating the mixture to 120 °C for 4 h, resulting in the disappearance of all substrate signals.

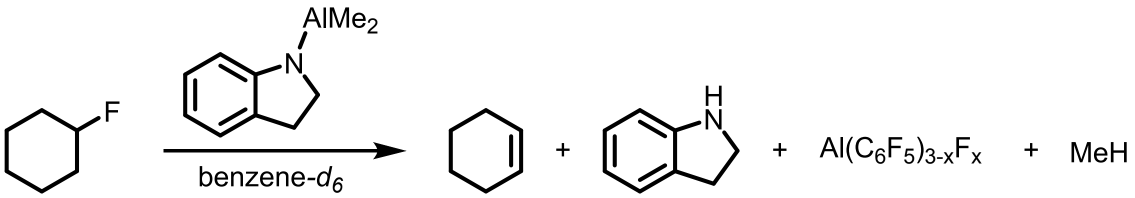

Scheme S8: DHF of fluorocyclohexane mediated by **(Ind)AlMe₂**.

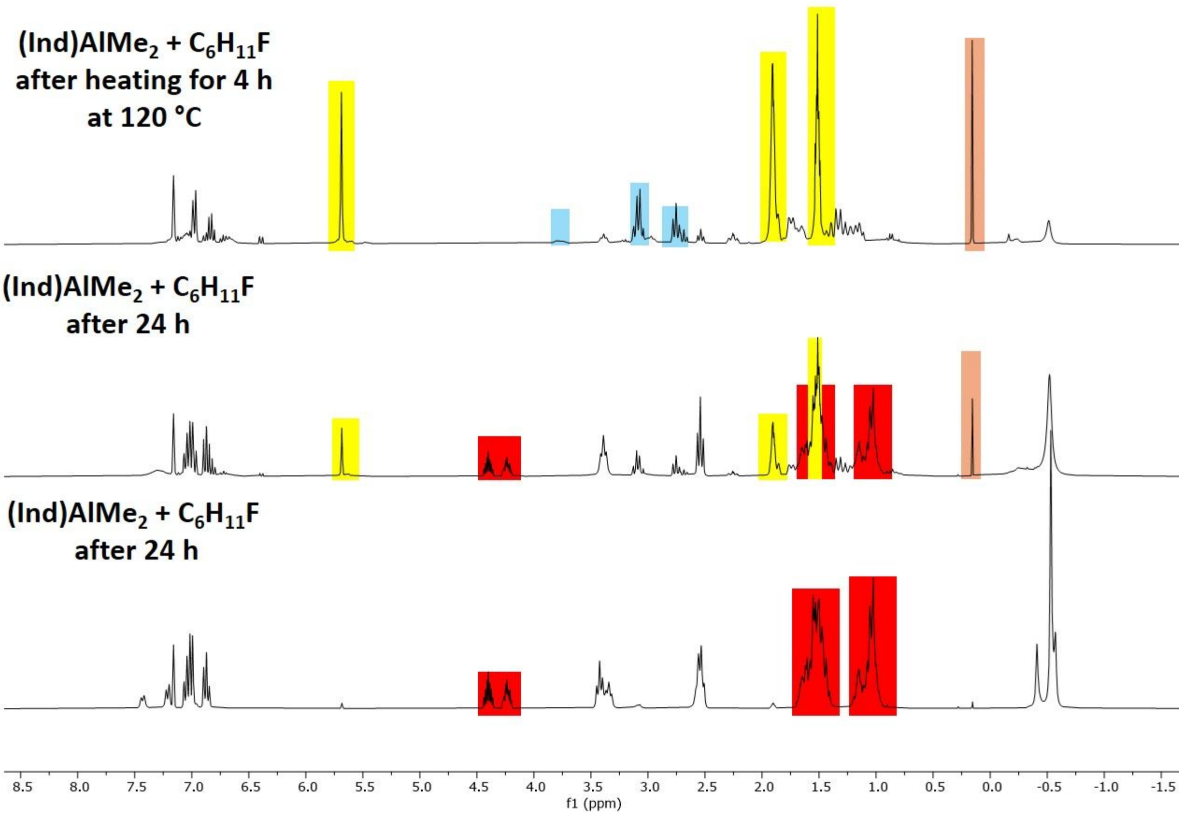

Figure S61. ^{1}H NMR stack plot (300 MHz, C_6D_6, 298 K) for the DHF of fluorocyclohexane mediated by **3** (red label = fluorocyclohexane, yellow label = cyclohexene, blue label = **(Ind)H**, green label = CH_4).

5.4. DHF mediated by AlMe$_3$

Full consumption of fluorocyclohexane was observed within 30 min after addition of $AlMe_3$. The ^{1}H and ^{19}F NMR spectra indicated complete conversion of the substrate to cyclohexene. Additionally, ^{1}H and ^{19}F NMR spectra revealed the formation of methane as well as $AlMe_{3-x}F_x$.[8] After complete conversion of the substrate, $AlMe_3$ was still detected in the reaction mixture. This observation indicates that $AlMe_3$ does not react in a 1:1 ratio with the substrate and that the resulting $AlMe_{3-x}F_x$ species can also mediate DHF.

Note: $AlMe_3$ was used as a 2 M stock solution in toluene. Therefore the ^{1}H NMR spectrum also displays the signals of solvate.

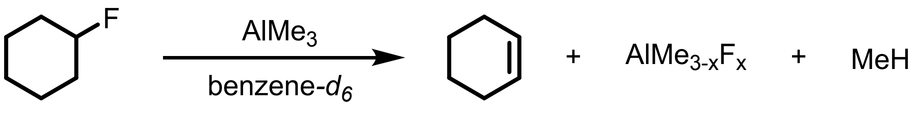

Scheme S9: DHF of fluorocyclohexane mediated by **AlMe₃**.

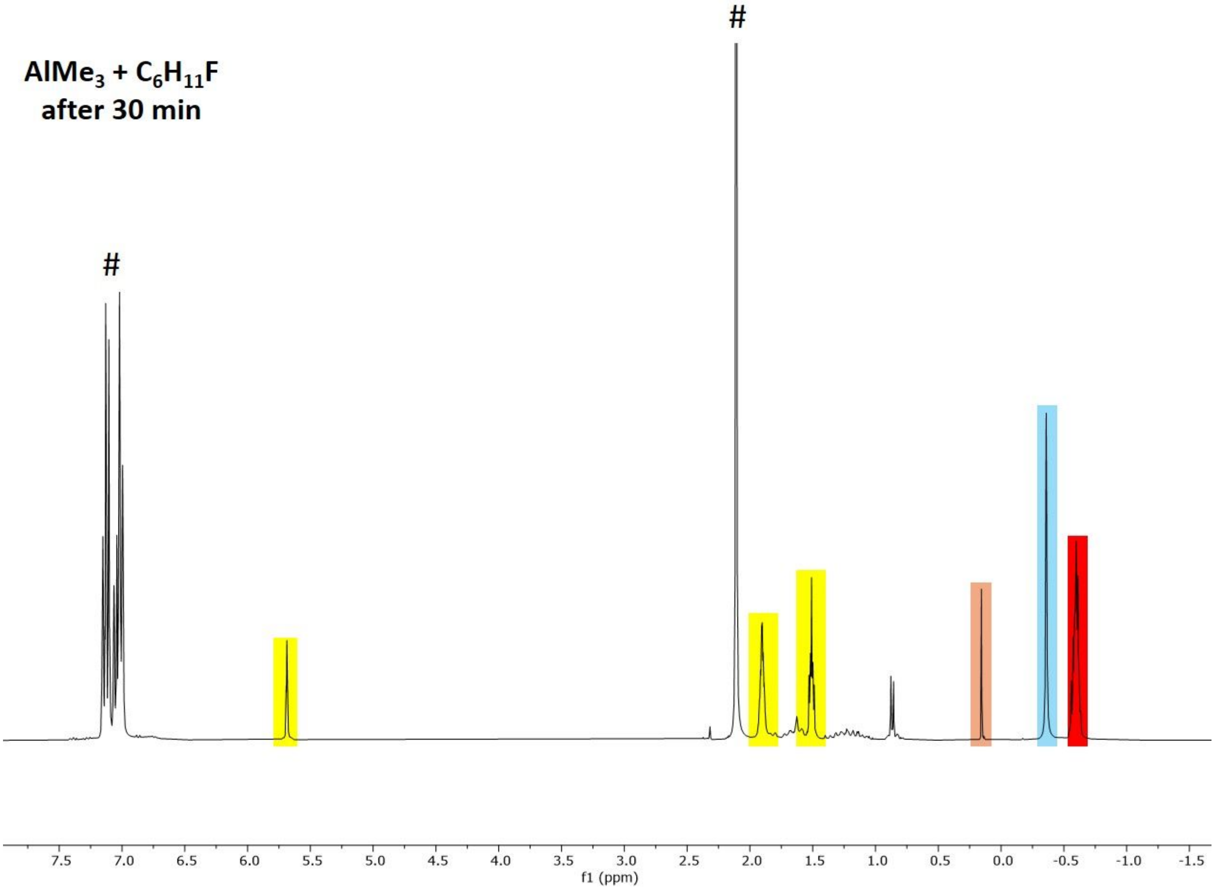

Figure S62. ^{1}H NMR spectrum (300 MHz, C_6D_6, 298 K) for the DHF of fluorocyclohexane mediated by **$AlMe_3$** (yellow label = cyclohexene, blue label **= $AlMe_3$**, green label = CH_4, red label = $AlMe_{3-x}F_x$, # = toluene).

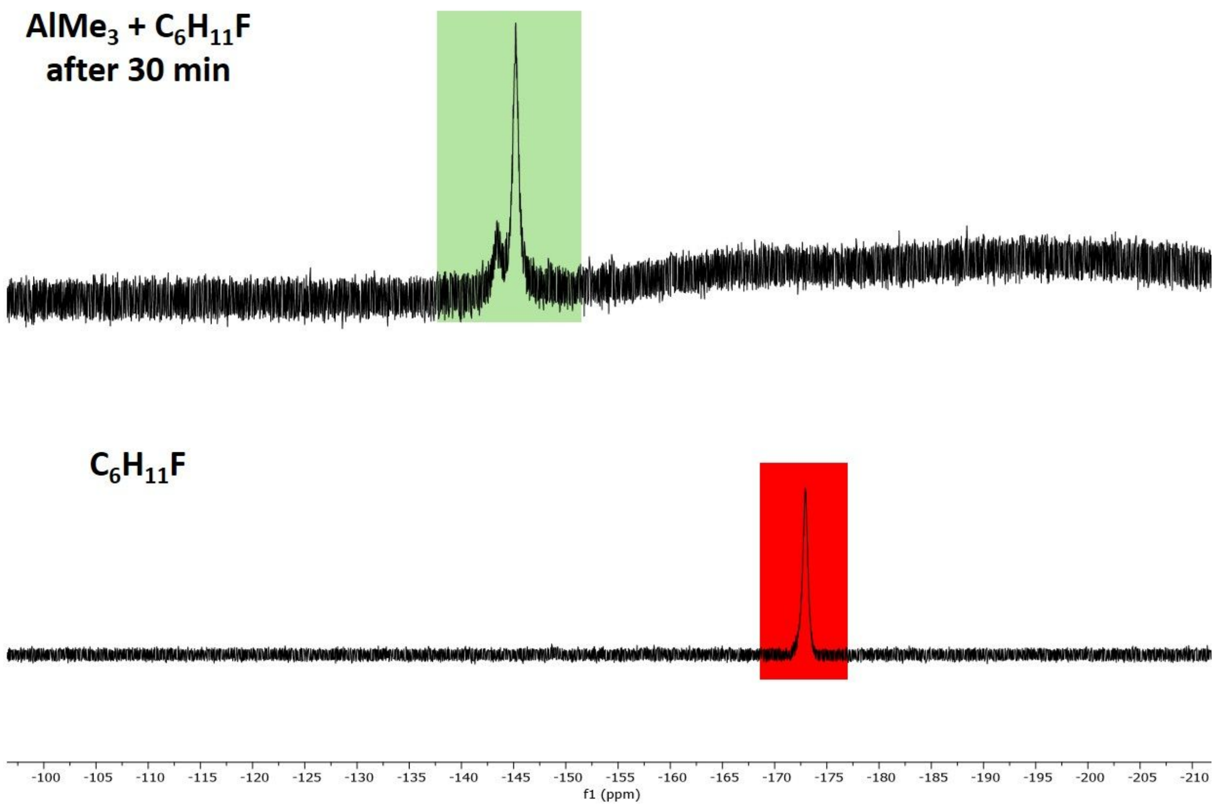

Figure S63. ^{19}F NMR stack plot (282 MHz, C_6D_6, 298 K) for the DHF of fluorocyclohexane mediated by $AlMe_3$ (green label = $AlMe_{3-x}F_x$, red label = $C_6H_{11}F$).

6. Crystallographic Data

The data collections were performed with a BRUKER D8 VENTURE area detector with Mo-*K*α radiation (λ = 0.71073 Å) at 100 K. Multi-scan absorption corrections implemented in SADABS[11] were applied to the data. The structures were solved by intrinsic phasing method (SHELXT-2013)[12] and refined by full matrix least square procedures based on *F*2 with all measured reflections (SHELXL-2014[13] the graphical user interfaces SHELXle[14]) with anisotropic temperature factors for all non-hydrogen atoms, except phenyl and methyl substituents. All hydrogen atoms were added geometrically and refined by using a riding model. The SQUEEZE[15] function implemented in PLATON was used for compound **1**.

CCDC numbers 2539165 – 2539170, 2539178 – 2539183 and 2539215 contain the supplementary crystallographic data for this paper. These data can be obtained free of charge from The Cambridge Crystallographic Data Centre via www.ccdc.cam.ac.uk/data_request/cif.

Comments to check-CIF alerts:

For compound **1’-THF** a large max. (positive) residual density of 1.72 eA^{-3} is reported. This is due to a poorly ordered THF solvate that has been modelled to a satisfactory level.

For compound **3** the value of R_{int} is greater than 0.18. This is because the crystals diffracted weakly, resulting in a high R_{int} value. Despite this, all data were consistent with the presented model.

For compound **4-CO_2** low-angle reflections were masked by the beamstop. The structure was refined with a satisfactory completeness, and the missing reflections do not affect the molecular model.

For compound **4’** an insufficient number of scans was collected during the X-ray diffraction measurement, resulting in limited high-angle data. Subsequent attempts to recrystallize the compound were undertaken; however, no crystalline material of sufficient quality for a complete X-ray analysis could be obtained.

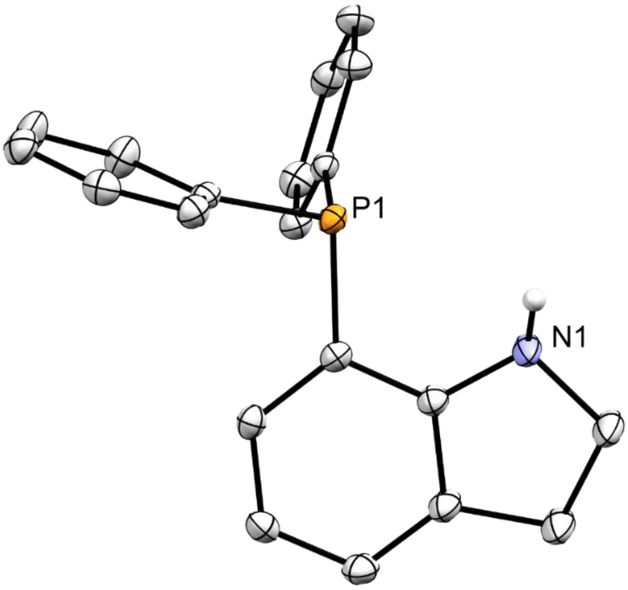

Figure S64. Molecular structure of **$Ph_2P(Ind)H$** as determined by X-ray diffraction analysis. H atoms omitted for clarity; thermal ellipsoids drawn at the 50% probability level.

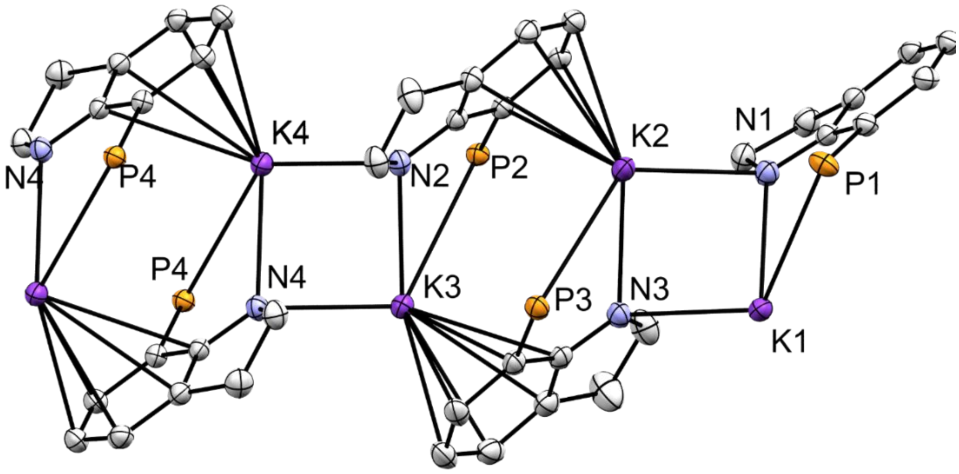

Figure S65. Structure of **$Ph_2P(Ind)K$** • 5(C_6H_6) as determined by X-ray diffraction analysis (forms a polymeric chain, only asymmetric unit is displayed). H atoms, co-crystallized solvents, PPh_2 substituents are omitted for clarity; thermal ellipsoids drawn at the 50% probability level.

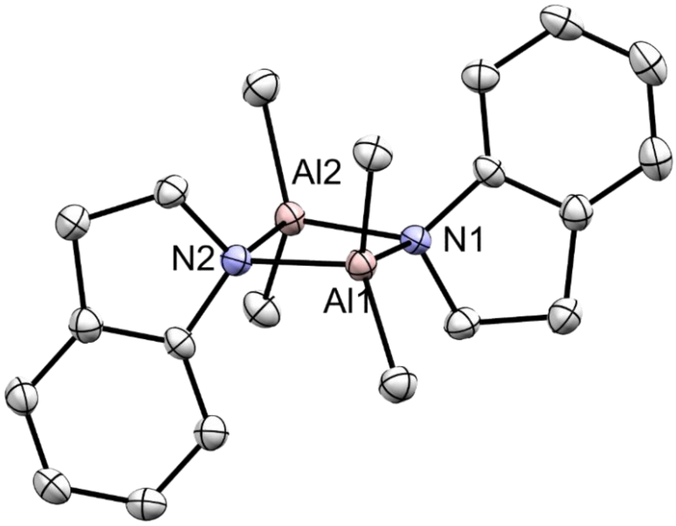

Figure S66. Molecular structure of **(Ind)AlMe$_2$** as determined by X-ray diffraction analysis. H atoms are omitted for clarity; thermal ellipsoids drawn at the 50% probability level.

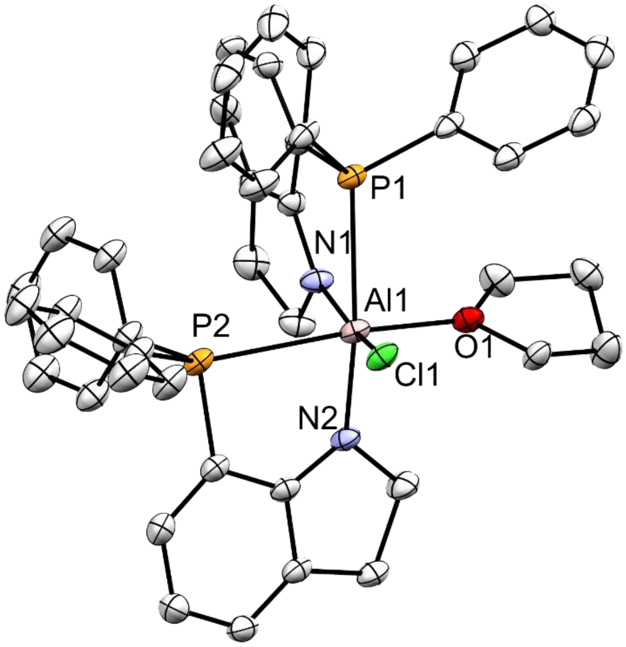

Figure S67. Molecular structure of **1'** • 2(C_4H_8O) as determined by X-ray diffraction analysis. H atoms and co-crystallized solvents are omitted for clarity; thermal ellipsoids drawn at the 50% probability level.

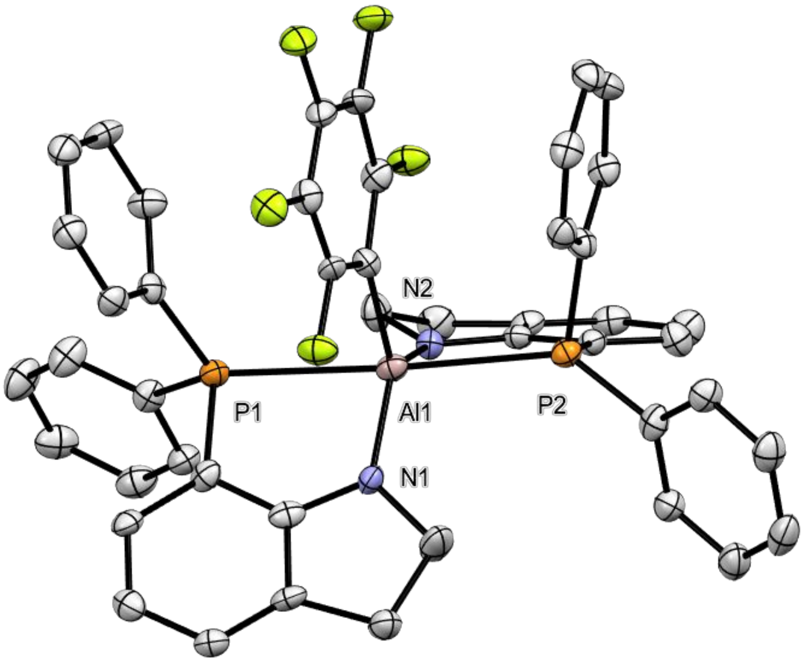

Figure S68. Molecular structure of **4’** as determined by X-ray diffraction analysis. H atoms are omitted for clarity; thermal ellipsoids drawn at the 50% probability level.

**Crystal structure**	**$Ph_2P(Ind)H$**	**$Ph_2P(Ind)K$**	**$(Ind)AlMe_2$**
CCDC Depositon No.	2539170	2539183	2539178
Chemical formula	$C_{20}H_{18}NP$	$(C_{20}H_{17}KNP)_4 \bullet 5(C_6H_6)$	$C_{20}H_{28}Al_2N_2$
M_r/g*mol^{-1}	303.34	1756.20	350.40
Temperature/K	100	100	100
Wave length/Å	0.71073	0.71073	0.71073
Crystal system	triclinic	triclinic	orthorhombic
Space group	*P*-1	*P*-1	*P*bca
a/Å	8.3757(4)	16.758(2)	7.3060(2)
b/Å	9.1606(5)	17.396(2)	17.6072(5)
c/Å	11.8788(6)	18.655(2)	30.4549(8)
α/°	76.341(2)	99.347(5)	90
β/°	71.440(2)	116.262(4)	90
γ/°	68.963(2)	101.476(5)	90
Cell volume/ Å^3	798.74(7)	4581.3(11)	3917.66(19)
Z	2	2	8
P_{calc}/g*cm^{-1}	1.261	1.273	1.188
μ($MoK_α$)mm^{-1}	0.168	0.316	0.152
F(0 0 0)	320.0	1844.0	1504.0
Crystal size/mm	0.330 x 0.200 x 0.170	0.150 x 0.130 x 0.030	0.140 x 0.050 x 0.020
Crystal color / shape	colorless fragment	yellow plate	colorless needle
2θ area/°	2.405 to 28.500	2.017 to 28.352	2.313 to 26.441
Measured reflexes	46416	227960	92838
Independent reflexes	4030	22846	4017
Parameters/ restrains	202/0	1170/300	221/0
R_{int}	0.0386	0.0382	0.0340
R_1 (I>2σ(I))	0.0327	0.0439	0.0419
wR_2 (all data)	0.0917	0.1187	0.1090
GooF (all data)	1.033	1.020	1.054
Flack-parameter	-	-	-
Max. u. min $Δρ_{elect.}$ /e Å^{-3}	0.332/-0.330	0.897/ -0.791	0.509/-0.318
Absorption correction	Multi-scan	Multi-scan	Multi-scan
Max/min. transmission	0.746/0.711	0.746/0.723	0.745/0.658

**Crystal structure**	**$[Ph_2P(Ind)AlCl_2]_2$, 1**	**$[Ph_2P(Ind)]_2AlCl(THF)$, 1'-THF**	**$[Ph_2P(Ind)AlI_2]_2$, 2**
CCDC Depositon Nr	2539179	2539180	2539165
Chemical formula	$C_{40}H_{34}Al_2Cl_4N_2P_2$	$C_{44}H_{42}AlClN_2OP_2$ • $2(C_4H_8O)$	$C_{40}H_{34}Al_2I_4N_2P_2$ •$3(C_6H_6)$
M_r/g*mol^{-1}	800.39	883.37	1.718
Temperature/K	100	100	100
Wave length/Å	0.71073	0.71073	0.71073
Crystal system	monoclinic	triclinic	monoclinic
Space group	*C*2/c	*P*-1	*P*2_1/n
a/Å	26.2194(18)	11.6273(5)	11.7253(10)
b/Å	14.6308(8)	11.9960(6)	35.310(4)
c/Å	24.6700(17)	17.2994(7)	13.5225(12)
α/°	90	74.522(2)	90
β/°	109.805(2)	72.113(2)	104.741(3)
γ/°	90	82.501(2)	90
Cell volume/ Å^3	8903.9(10)	2209.93(17)	5414.3(9)
Z	8	2	4
P_{calc}/g*cm^{-1}	1.194	1.328	1.718
μ($MoK_α$)mm^{-1}	0.405	0.226	2.433
F(0 0 0)	3296.0	936.0	2728.0
Crystal size/mm	0.210 x 0.160 x 0.040	0.170 × 0.160 × 0.010	0.140 x 0.040 x 0.040
Crystal color / shape	colorless plate	yellow block	colorless block
2θ area/°	1.960 to 26.842	2.317 to 26.427	2.135 to 28.306
Measured reflexes	137681	87598	37293
Independent reflexes	9516	9072	7361
Parameters/ restrains	451/169	595/1412	613/993
R_{int}	0.0320	0.074	0.1350
R_1 (I>2σ(I))	0.0337	0.0531	0.0464
wR_2 (all data)	0.0818	0.1481	0.0905
GooF (all data)	1.026	1.035	0.821
Flack-parameter	-	-	-
Max. u. min $Δρ_{elect.}$ /e Å^{-3}	0.287/-0.263	1.72/−1.36	0.969/-1.991
Absorption correction	Multi-scan	Multi-scan	Multi-scan
Max/min. transmission	0.745/0.670	0.745/0.686	0.746/0.645

**Crystal structure**	**$Ph_2P(Ind)AlMe_2$, 3**	**$Ph_2P(Ind)Al(C_6F_5)_2$, 4**	**$[Ph_2P(Ind)]_2Al(C_6F_5)$, 4'**
CCDC Depositon Nr	2539166	2539169	2539215
Chemical formula	$C_{22}H_{23}AlNP$	$C_{32}H_{17}AlF_{10}NP$	$C_{46}H_{34}AlF_5N_2P_2$
M_r/g*mol^{-1}	359.39	663,43	798.67
Temperature/K	100	115	100
Wave length/Å	0.71073	0.71073	0.71073
Crystal system	monoclinic	triclinic	monoclinic
Space group	*P*2$_1$/n	*P*-1	*P*2$_1$/n
a/Å	13.151(3)	10.6728(8)	14.963(2)
b/Å	13.986(3)	10.7894(9)	18.582(2)
c/Å	10.684(2)	13.7206(11)	15.200(2)
α/°	90	78.518(3)	90
β/°	93.228(6)	67.938(3)	118.048(4)
γ/°	90	73.507(3)	90
Cell volume/ Å^3	1962.1(7)	1396.4(2)	3730.1(9)
Z	4	2	4
P_{calc}/g*cm^{-1}	1.217	1.578	1.422
μ($MoK_α$)mm^{-1}	0.189	0.222	0.203
F(0 0 0)	760.0	668.0	1648.0
Crystal size/mm	0.240 x 0.040 x 0.030	0.420 x 0.310 x 0.240	0.120 × 0.100 × 0.050
Crystal color / shape	colorless needle	colorless block	yellow block
2θ area/°	2.127 to 24.993	1.979 to 33.283	1.892 to 24.082
Measured reflexes	29004	111088	19703
Independent reflexes	3462	10721	5909
Parameters/ restrains	229/0	406/0	505/0
R_{int}	0.1334	0.0200	0.129
R_1 (I>2σ(I))	0.0588	0.036	0.058
wR_2 (all data)	0.1903	0.1111	0.1576
GooF (all data)	0.906	0.998	0.081
Flack-parameter	-	-	-
Max. u. min $Δρ_{elect.}$ /e Å^{-3}	0.321/–0.367	0.539/–0.238	0.350/–0.350
Absorption correction	Multi-scan	Multi-scan	Multi-scan
Max/min. transmission	0.745/0.519	0.747/0.703	0.745/0.656

**Crystal structure**	**$Ph_2P(Ind)Al(Me)_2(Ph_2CN_2)$, 3-CNN**	**$Ph_2P(Ind)Al(C_6F_5)_2(Ph_2CN_2)$, 4-CNN**	**$Ph_2P(Ind)AlMe_2(CO_2)$, 3-CO_2**
CCDC Depositon Nr	2539167	2539181	2539168
Chemical formula	$C_{35}H_{33}AlN_3P$	$C_{45}H_{27}AlF_{10}N_3P$	$C_{46}H_{46}Al_2N_2O_4P_2$
M_r/g*mol^{-1}	553.59	857.64	806.75
Temperature/K	100	100	101
Wave length/Å	0.71073	0.71073	0.71073
Crystal system	triclinic	triclinic	monoclinic
Space group	*P*-1	*P*-1	$P2_1$/n
a/Å	10.000(2)	11.2993(10)	12.5345(5)
b/Å	11.449(3)	13.7800(12)	8.8622(5)
c/Å	14.068(3)	14.7782(12)	19.7554(11)
α/°	94.502(8)	76.682(3)	90
β/°	93.933(8)	68.650(3)	101.677(2)
γ/°	110.353(8)	66.441(3)	90
Cell volume/ Å^3	1497.5(5)	1954.8(3)	2149.08(19)
Z	2	2	2
P_{calc}/g*cm^{-1}	1.228	1.457	1.247
μ(MoK_α)mm^{-1}	0.150	0.179	0.187
F(0 0 0)	584.0	872.0	848.0
Crystal size/mm	0.280 x 0.140 x 0.070	0.250 x 0.080 x 0.020	0.180 x 0.160 x 0.160
Crystal color / shape	colorless fragment	colorless needle	colorless fragment
2θ area/°	2.184 to 30.708	2.065 to 25.518	2.137 to 30.628
Measured reflexes	46238	75648	75003
Independent reflexes	5269	5926	5271
Parameters/ restrains	364/0	541/574	255/0
R_{int}	0.1008	0.0647	0.0595
R_1 (I>2σ(I))	0.0654	0.0461	0.0479
wR_2 (all data)	0.1524	0.1239	0.1262
GooF (all data)	1.028	1.036	1.057
Flack-parameter	-	-	-
Max. u. min $\Delta\rho_{elect.}$ /e Å^{-3}	0.493/ -0.572	0.562/-0.410	0.376/-0.403
Absorption correction	Multi-scan	Multi-scan	Multi-scan
Max/min. transmission	0.746/0.613	0.745/0.673	0.746/0.669

**Crystal structure**	**$Ph_2P(Ind)Al(C_6F_5)_2(CO_2)$, 4-$CO_2$**	-	-
CCDC Depositon Nr	2539182		
Chemical formula	$[C_{66}H_{36}Al_2F_{20}N_2O_4P_2]_2$ • $7(C_6H_6)$		
M_r/g*mol^{-1}	3376.45		
Temperature/K	100		
Wave length/Å	0.71073		
Crystal system	triclinic		
Space group	*P*-1		
a/Å	14.6158(11)		
b/Å	20.8907(18)		
c/Å	26.672(2)		
α/°	108.218(3)		
β/°	94.911(3)		
γ/°	93.285(3)		
Cell volume/ Å^3	7676.9(11)		
Z	2		
P_{calc}/g*cm^{-1}	1.461		
μ($MoK_α$)mm^{-1}	0.183		
F(0 0 0)	3436.0		
Crystal size/mm	0.300 x 0.170 x 0.080		
Crystal color / shape	colorless block		
2θ area/°	1.927 to 26.446		
Measured reflexes	252356		
Independent reflexes	31555		
Parameters/ restrains	2162/3732		
R_{int}	0.0760		
R_1 (I>2σ(I))	0.0480		
wR_2 (all data)	0.1380		
GooF (all data)	1.030		
Flack-parameter	-		
Max. u. min $Δρ_{elect.}$ /e Å^{-3}	0.63/–0.43		
Absorption correction	Multi-scan		
Max/min. transmission	0.745/0.664		

7. References

[1] J. Dong, Q. Xia, X. Lv, C. Yan, H. Song, Y. Liu, Q. Wang, *Org. Lett.* **2018**, *20*, 5661-5665.
[2] Bochmann, M. J. Sarsfield, *Organometallics* **1998**, *17*, 5908-5912.
[3] S. Styra, M. Radius, E. Moos, A. Bihlmeier, F. Breher, *Chem. Eur. J.* **2016**, *22*, 9508-9512.
[4] M. I. Javed, M. Brewer, *Org. Synth.* **2008**, *85*, 189.
[5] S. Kundu, S. Sinhababu, M. M. Siddiqui, A. V. Luebben, B. Dittrich, T. Yang, G. Frenking, H. W. Roesky, *J. Am. Chem. Soc.* **2018**, *140*, 9409-9412.
[6] N. Bernd, Dissertation thesis, Ludwig-Maximilians-Universität München **2006**.
[7] a) U. Mayer, V. Gutmann, W. Gerger, *Monatsh. Chem.* **1975**, 106, 1235; b) M. A. Beckett, G. C. Strickland, J. R. Holland, K. S. Varma, *Polymer* **1996**, 37, 4629.
[8] N. A. Jenek, S. L. Brock, J. Mao, A. A. Fogh, A. Phanopoulos, M. R. Crimmin, *Nat. Chem.* **2026**.
[9] L. Oliva, P. Oliva, N. Galdi, C. Pellecchia, L. Sian, A. Macchioni, C. Zuccaccia, *Angew. Chem. Int. Ed.* **2017**, *56*, 14227-14231.
[10] C. Schnitter, K. Klimek, H. W. Roesky, T. Albers, H.-G. Schmidt, C. Röpken, E. Parisini, *Organometallics* **1998**, *17*, 2249-2257.
[11] G. M. Sheldrick, SADABS 1996, University of Göttingen, Germany.
[12] G. M. Sheldrick, *Acta Crystallogr. A* **2015**, 71, 3.
[13] G. M. Sheldrick, *Acta Crystallogr. C* **2015**, 71, 3.
[14] C. B. Hübschle, G. M. Sheldrick, B. Dittrich, *J. Appl. Cryst.* **2011**, 44, 1281-1284.
[15] A. L. Spek, Acta *Crystallogr. D, Biological crystallography* **2009**, 65, 148.
